# Can transparency and accountability programs improve health? Experimental evidence from Indonesia and Tanzania^☆^

**DOI:** 10.1016/j.worlddev.2020.105369

**Published:** 2021-06

**Authors:** Jean Arkedis, Jessica Creighton, Akshay Dixit, Archon Fung, Stephen Kosack, Dan Levy, Courtney Tolmie

**Affiliations:** a Results for Development, 1111 19th Street NW, Suite 700, Washington DC 20036, USA; b Harvard University, Harvard Kennedy School, 79 JFK Street, Cambridge, MA 02138, USA; c University of Washington, Evans School of Public Policy and Governance, 4105 George Washington Lane Northeast, Seattle, WA 98105, USA

**Keywords:** Transparency, Accountability, Community participation, Maternal and newborn health, Indonesia, Tanzania

## Abstract

We assess the impact of a transparency and accountability program designed to improve maternal and newborn health (MNH) outcomes in Indonesia and Tanzania. Co-designed with local partner organizations to be community-led and non-prescriptive, the program sought to encourage community participation to address local barriers in access to high quality care for pregnant women and infants. We evaluate the impact of this program through randomized controlled trials (RCTs), involving 100 treatment and 100 control communities in each country. We find that on average, this program did not have a statistically significant impact on the use or content of maternal and newborn health services, nor on perceptions of civic efficacy or civic participation among recent mothers in the communities where it was offered. These findings hold in both countries and in a set of prespecified subgroups. To identify reasons for the lack of impacts, we use a mixed-method approach combining interviews, observations, surveys, focus groups, and ethnographic studies that together provide an in-depth assessment of the complex causal paths linking participation in the program to improvements in MNH outcomes. Although participation in program meetings was substantial and sustained in most communities, and most attempted at least some of what they had planned, only a minority achieved tangible improvements, and fewer still saw more than one such success. In our assessment, the main explanation for the lack of impact is that few communities were able to traverse the complex causal paths from planning actions to accomplishing tangible improvements in their access to quality health care.

## Highlights

• We assess the impact of a transparency & accountability program on health outcomes.
• We use randomized controlled trials involving 400 villages across Indonesia and Tanzania.
• On average, no evidence of a treatment effect on maternal and newborn healthcare.
• The causal paths from planning actions to tangibly improving healthcare were complex.
• Overall, few communities were able to traverse these complex causal paths.

## 1 Introduction

In 2017, over 4 million children died within the first 28 days of life (Dicker et al., 2018), most from diseases and complications that are readily preventable or treatable with proven, cost-effective healthcare services (You et al., 2015). In recent years, citizen and community scorecards, social audits, and other programs that try to increase transparency and accountability have been widely explored as tools to improve access to and quality of public services. One premise of these programs is that information can empower citizens to improve the responsiveness, accountability, and ultimately the effectiveness of their public services (Fox, 2007, Glennerster, 2005, McGee and Gaventa, 2011, Molina et al., 2017). This premise has been validated by several studies, notably Björkman and Svensson (2009), who find that a community scorecard program reduced infant mortality by a third in one year.^1^ However, other studies show little or no effect of these programs.^2^ The overall picture that emerges from the empirical literature is mixed (Fox, 2015, Joshi and Houtzager, 2012, Olken and Pande, 2011, Joshi, 2010, McGee and Gaventa, 2011, Kosack and Fung, 2014).

In this paper, we present results from randomized controlled trials (RCTs) from the Transparency for Development (T4D) project of a non-prescriptive, community-led transparency and accountability program designed to encourage civic participation to improve access to high quality maternal and newborn healthcare. The T4D program was co-designed and piloted over a two-year period with local partner organizations^3^ and then offered in one hundred communities each in Indonesia and Tanzania, two contexts that differ substantially in terms of access to quality healthcare, choice among health service providers, as well as general levels of economic development. We leverage a mixed-methods approach combining the RCTs with focus group discussions, ethnographic studies, interviews, and systematic observations to evaluate the effects of the program on health outcomes and civic participation in these communities.

At a high level, the T4D program involved a trained facilitator from the partner organizations identifying a group of interested community members, informing them of MNH-related problems in their community, and helping them develop a plan of social actions to overcome problems with access to quality maternal and newborn health care in their communities. We focus on the impact of this program on the quality^4^ and use of maternal and newborn health care services in communities in which the program was offered, as well as infant health outcomes, and civic participation and perceptions of civic efficacy among recently pregnant women in the broader community. For these pre-registered primary outcomes, we find that the T4D program had no significant average effects.

We use the interviews, surveys, focus groups, systematic observations, ethnographic studies, and other qualitative data to help explain these null average observed effects.^5^ As others have noted, the causal chains linking transparency and accountability programs to improvements in public services are lengthy and complex.^6^ We propose a framework of causal paths that may link participation in the T4D program to improvements in maternal and newborn healthcare services, health, and civic empowerment, and assess the program’s effects on each step of that framework.

Overall, we find that community participation in both countries was substantial and sustained, and that the meetings created space for participants to leverage local knowledge and collectively plan diverse courses of action for improving their maternal and newborn health care. In most communities, those who participated in the meetings subsequently tried at least some of the actions they had planned. Many tried to educate their neighbors about the importance of delivering in a facility and seeking antenatal and postnatal care as well as to improve their health care services in other ways. In 87% of communities, participants in the final program meeting described having completed at least one of these activities. Yet interviews at the time with participants and those with whom they had planned to engage suggest that the proportion of communities in which participants succeeded at their goal was closer to 45%. When participants were asked to reflect on the program approximately a year and a half later, they could recall a specific, tangible improvement from their efforts in only 35% of communities (41% in Indonesia; 30% in Tanzania). The proportion of communities in which participants were able to recall at least two tangible improvements from their efforts—progress that might indicate broader rather than one-off improvements in access to quality care—was still lower: only 14% of communities in Indonesia and 4% in Tanzania.

In short, we do not find evidence that participants were able to achieve measurable improvements to maternal and newborn health care in the average community offered the program, and our main explanation for these findings is that participants in few communities were able to traverse the complete paths from planning actions to realizing improvements in their maternal and newborn health care.^7^

To our knowledge, our paper is the first to explore the experimentally identified effects of a similar transparency and accountability program across two diverse countries on two continents, and one of a relatively small number of papers to use RCTs to estimate the effects of a development program beyond a single country context. We also contribute an extensive analysis of qualitative data indicating from several perspectives similar reasons for the lack of significant effect we observe. Subsequent papers will describe extensive evidence of additional similarities: in participants’ responses, reflections on the efficacy of their efforts, as well as in the broader historical dynamics in these communities and countries that influenced their experiences and the effects of their efforts. Altogether these similarities between the two countries suggests that our findings in this paper may generalize, at least for similar programs in contemporary contexts similar to Indonesia and Tanzania.^8^

The rest of this paper is structured as follows. In 2, 3, we present an overview of the existing literature and the contexts in Indonesia and Tanzania where the program was offered. We describe the program and research design in 4, 5. In Section 6, we present our findings from the RCTs. We unpack and triangulate the findings in Section 7, and conclude with Section 8.

## 2 The Literature

The promise of transparency and accountability programs for improving the quality of public services or governance has been subjected to a growing number of empirical tests in recent years. Conclusions have been mixed. A prominent study in Uganda found remarkable improvements in maternal and newborn health soon after a community scorecard program encouraged bottom-up community monitoring (Björkman & Svensson, 2009). Banerjee, Hanna, Kyle, Olken, and Sumarto (2018) find that citizens in villages in Indonesia who had been informed of their rice subsidy entitlements received 26% more subsidy on net, and Andrabi, Das, and Khwaja (2017) find that providing information to citizens in Pakistan on test scores in education markets with multiple public and private providers improved subsequent education outcomes. Fiala and Premand (2017) find positive effects from grassroots monitoring of a community-driven development program on the quality of a range of community projects in Uganda. Reinikka and Svensson, 2005, Reinikka and Svensson, 2011 find that newspaper campaigns in Uganda, which provided information to communities about schooling funds that they were entitled to receive and that they actually received, reduced leakage of funds and significantly improved test scores.

However, a large number of other studies have found little or no effects of these programs. Banerjee et al., 2010, Lieberman et al., 2014 find no evidence of impact on education from offering parents information about the low quality of the education offered in their children’s schools in India and Kenya, respectively. Olken (2007) finds that a community monitoring intervention in Indonesia had little impact on corruption. In Benin, Keefer and Khemani (2014) find that community radio broadcasts improved education outcomes, but that these improvements were driven by parental investments in children’s education rather than government responsiveness or collective action. Raffler, Posner, and Parkerson (2019) find that even an information and mobilization intervention modeled closely after Björkman and Svensson (2009) and evaluated fifteen years after the original study in the same country, Uganda, had little detectable effect on healthcare utilization or outcomes.

In addition to the experimental literature, qualitative and mixed-methods research have also reached mixed conclusions, and provide insight into the mixed evidence related to transparency and accountability interventions in health. One study of a well-known accountability approach (Citizen Voice and Action) implemented in Zambia shows some evidence of improved governance and health service outcomes; however the mechanisms through which these changes happened required political buy-in and existing structures for interactions between citizens and government, limiting the scalability of these findings (Schaaf et al., 2017). Reviewing research on social accountability for maternal and newborn health in sub-Saharan Africa, Hilber et al. (2016) find that several factors related to the mechanisms and actors involved appear to be required for that accountability efforts to be associated with positive health outcomes.

Other reviews and meta-analyses have also failed to reach consensus: Holland and Schatz, 2016, Molina et al., 2017 find mostly positive effects, White, Menon, and Waddington (2018) find that very few have had much effect and that most struggled even to encourage much civic participation, and Kosack and Fung, 2014, Fox, 2015 find mixed effects. Communities are often able to improve their public services when they decide on their own to try (Mansuri & Rao, 2012). Yet overall, it remains debatable whether, how, and in what contexts transparency and accountability programs can encourage communities to measurably improve public services.

## 3 Contexts

Indonesia is a lower-middle income country in Southeast Asia with 2017 GDP per capita of USD 3846 (World Bank, 2017) that has experienced multiple peaceful transfers of power since the fall of an authoritarian regime in 1998 (Freedom House, 2018a). The T4D transparency and accountability program was offered to communities in two provinces on two of Indonesia’s eighteen thousand islands: Banten and South Sulawesi.

In a household survey conducted at baseline, most respondents in these provinces resided in sturdy dwellings with walls made of stone (48%) or wood (37%), and 99% households had electricity. Both also had relatively high levels of access to MNH care at baseline. The MNH service delivery system in Indonesia involves a diverse range of providers. The focal point of public services is usually a public health center that operates at the sub-district level, known as “puskesmas.” Puskesmas provide comprehensive basic health services, generally including delivery services. They are the lowest level public health service center overseen directly by the Indonesian government, and every village is assigned to the catchment area of a puskesmas. A baseline survey of facilities in sample areas indicated that the average puskesmas served 8.8 villages and had close to 57 staff, with nearly 14 in the maternal/delivery unit alone. Nearly all surveyed puskesmas had electricity and 95.5% used water from an improved source. A puskesmas often oversees and is supported by a network of smaller health centers, or “poskesdes.” Indonesia also has long had a nationwide community-based health program known as “posyandu.” Staffed by a midwife and local volunteers, posyandu offer monthly antenatal care services to pregnant women, and growth monitoring and vaccination programs for children under the age of five. At baseline, 99% of villages in our sample had a puskesmas, a smaller health center overseen by a puskesmas, a village midwife operating under the supervision of the puskesmas, a birthing clinic, or a private provider located within the village. Almost all baseline respondents (82%) reported having more than one provider or facility from which they could seek care.

Yet respondents in sample areas also described a mixed experience with the quality of their care. For example, nearly all respondents received some form of antenatal care, and close to 90% of respondents said they had completed the recommended four ANC visits during pregnancy (Table 1). But only 55% of respondents had given birth at a facility, and only 22% of baseline respondents reported receiving three fundamental components of ANC: having their blood pressure checked, having a urine sample drawn and receiving a report of the results, and having a blood sample drawn and receiving a report of the results.^9^

**Table 1:** Baseline sample means for MNH outcomes in Indonesia and Tanzania.^34^

Primary MNH Outcomes	Indonesia (% baseline respondents)	Tanzania (% baseline respondents)
Whether the respondent had a first antenatal care visit within the first trimester	69	19
Whether the respondent attended four or more antenatal care visits over the course of the pregnancy	87	43
Whether the respondent gave birth with a skilled provider	79	56
Whether the respondent gave birth at a health facility	55	56
Stunting – Whether the infant is below 2 standard deviations from the median WHO Child Growth Standards	16	27
Underweight – Whether the infant is below 2 standard deviations from the median WHO Child Growth Standards	16	9

34 Two additional primary outcomes (utilization of postpartum and postnatal care) are not included in this table because the associated questions were phrased differently at baseline and endline. At endline, respondents were asked about postpartum/postnatal care checks conducted *after* leaving the birth facility and within 7 days of giving birth. At baseline, however, respondents were asked about postpartum/postnatal care checks conducted within 7 days of giving birth, *irrespective* of whether they received this care before or after leaving the birth facility.

The context in Tanzania differs in several respects of potential relevance to our inquiry. Tanzania’s GDP per capita is only a quarter of that of Indonesia (World Bank, 2017), and although elections have been a regular feature of the political landscape since the 1990s, the ruling party has retained power for over half a century (Freedom House, 2018b). The T4D program was implemented in two regions, Dodoma and Tanga, in which the average household was resource poor relative to those in Indonesia. In a household survey in communities at baseline, most respondents lived in dwellings made of mud (71.3%), and only 12.5% had access to electricity.

The ecosystem of public provision of MNH care in Tanzania also involves fewer service providers than in Indonesia. The primary public health facility for most communities in Tanzania is a dispensary. Similar to puskesmas in Indonesia, every village is assigned to the catchment area of a dispensary. However, Tanzanian dispensaries tend to be much smaller than Indonesian puskesmas: at baseline, the average dispensary we surveyed in Tanga and Dodoma served 2.7 villages and had 6.4 staff members. Electricity from the grid was the primary power source for only about a quarter of dispensaries at baseline (21.6%). Nearly half (44.4%) reported not having a regular supply of water. Facilities in Tanzania were also less common than in Indonesia: less than two-thirds (62.5%) of villages we surveyed had a dispensary located within the village. Nearly three quarters of respondents to our household survey (73.5%) reported not being able to choose among more than one health facility.

Our baseline surveys also indicate that the quality of this care tended to be lower than in Indonesia. Antenatal care again offers a clear illustration: While the vast majority of women surveyed at baseline (98.4%) had received some form of antenatal care (ANC), less than half (43.4%) had completed the recommended minimum of four antenatal visits during their most recent pregnancy (compared with 90% in Indonesia; see Table 1). Similar to Indonesia, slightly over half of respondents (56%) reported having given birth at a facility. The quality of ANC was mixed, with less than a third (30.9%) receiving the three basic components of ANC described above.^10^

In short, relative to Indonesia, communities in Tanzania had fewer economic resources, a public health ecosystem with fewer providers in a lower resource setting offering less choice among providers for those seeking MNH care, and lower baseline outcomes in terms of utilization or quality of MNH care.

## 4 The T4D program

T4D was a transparency and accountability program developed over a two-year iterative co-design and piloting process with staff from partner organizations in each country. The initial design was based on the “community scorecard” approach that Björkman and Svensson (2009) found to have dramatically improved maternal and newborn healthcare in Uganda, and was subsequently defined, piloted, and iteratively refined over multiple rounds of design and feedback discussions.^11^ The design process followed several principles. First, every aspect was co-designed with in-country partners based on their local knowledge of what was appropriate in their respective context. Rather than necessarily fixing health service delivery, the focus was on improving health: in particular an area of health—the health of pregnant women and infants—of concern both in Indonesia and Tanzania as well as in the global health community. The program was designed to be non-prescriptive and locally relevant: offering space for communities in widely different circumstances to leverage local knowledge and collectively decide on context-appropriate activities for improving maternal and newborn health. In addition, the program was designed to be community-driven (to emphasize the importance of participants using their own knowledge and capacity to understand and fix problems) and offered no other material, financial, technical, or relational resources, so that participants would rely on their existing willingness and capacities to try to improve their maternal and newborn health. Finally, the program was designed to be relatively light-touch and scalable, so that it could be consistently implemented across a large number of diverse communities. To maximize comparability, the program’s core components were similar across the two countries. The specifics of the approach were defined, iteratively refined, and validated over the two-year co-design process through a round of “pre-piloting” (quick tests of individual components) in Indonesia and complete pilots (the entire program from start to finish) in both Indonesia and Tanzania.

Broadly, the resulting program was a series of six meetings between a facilitator employed by the partner organizations and a group of citizens from a village, organized over a period of approximately three months. The purpose of these meetings was to offer information and facilitated discussion that would encourage citizens to try to alleviate problems with their maternal and newborn health care that affected them and their neighbors. The program began with two day-long meetings of a small group of 15–16 people from the community whom they had identified over the previous weeks as interested in participating in the meetings as community representatives (CRs).^12^ At these meetings, the facilitator first shared information they had gathered over the previous weeks on three key health indicators in the community: antenatal care visits of pregnant women (in Tanzania) or those with birth preparedness plans (in Indonesia), and how often women gave birth in health facilities and sought postnatal care services. As participants discussed why these indicators were not higher, the facilitator also shared information they had gathered from surveys in the community on a range of barriers that might be preventing more pregnant women in the community from delivering at a facility or seeking antenatal or postnatal care, as well as stories illustrating approaches other communities in the area had taken to improve their services. Finally, the facilitator helped those attending the meetings formulate plans of activities that they could undertake to address the problems with maternal and newborn health in their community.

Immediately following the first two meetings was an open meeting, where those still interested in pursuing their planned activities presented them to the broader community. The last three meetings occurred at approximately 30-day intervals. At each, the facilitator met with the participants to learn about their progress and to help them reflect on challenges they may have faced. At the sixth and final meeting, the facilitator also encouraged them to plan for how they would continue to meet and sustain their progress after the facilitator was no longer organizing meetings.

No additional resources or help were provided to participants in these meetings.^13^ Rather, throughout the program it was entirely up to the participants to decide what, if anything, to do to try to improve maternal and newborn health care in their particular community context, using only their existing resources and capacities as members of their communities and citizens of Indonesia or Tanzania.

**Fig. 1:**
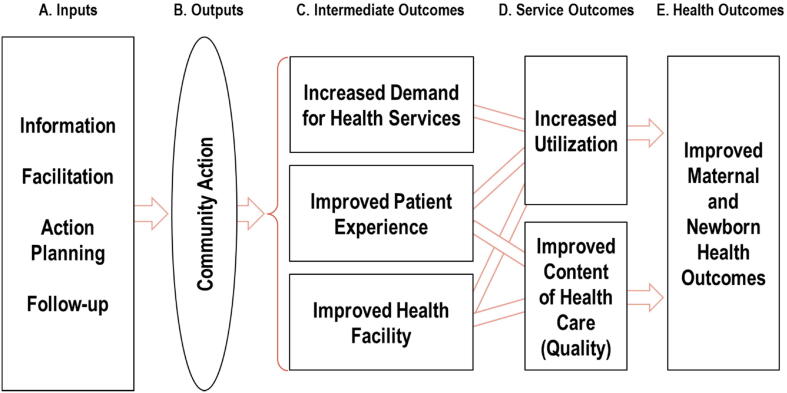
Framework linking participation in the T4D program to MNH outcomes.

As noted above, the causal chains linking such programs to improvements in public services are complex. In Fig. 1, we present a framework summarizing several possible causal chains that may link participation in the program to improvements in maternal and newborn health as, first, inputs, leading to outputs, and then to intermediate outcomes, service outcomes, and finally health outcomes. This framework will enable us to trace the varied causal paths from participation to impact, which we shall return to in Section 7. Column A represents the T4D program: the “inputs” offered to each community in the treatment group. Column B represents any activities that participants in the program may try to improve access to high quality maternal and newborn health care services in their communities. Column C represents intermediate improvements resulting from Column B: in aspects of the facility, in patients’ experiences when seeking care, and in increased demand among the community for care. The intended outcomes on the use of higher quality health care services are represented in Column D, which in turn may lead to healthier mothers and infants (Column E).

## 5 Research design and data

In this paper, we assess the hypothesis that participating in this program would empower people across diverse environments to act in ways that would improve the access and quality of the maternal and newborn healthcare services available to pregnant women and small children. The observable implication of this hypothesis is that across diverse contexts, the quality and use of their care would be significantly higher than in other communities who were not offered the program. We leverage an experimental design as well as focus group discussions, interviews, surveys, systematic observations, ethnographic studies, and other qualitative data to present a comprehensive assessment of this observable implication. In particular, we examine both *how* the program worked as well as *whether* it was effective at improving MNH outcomes in these two contexts.

The study consisted of RCTs in both Indonesia and Tanzania, with a “treatment” group of 100 villages and a “control” group of 100 villages in each country. In Indonesia, 85 of the 200 villages were in Banten and 115 in South Sulawesi, and in Tanzania 77 villages were in Dodoma and 123 in Tanga. This section describes the following aspects of the research design: (a) randomization into treatment and control groups, (b) baseline equivalence between treatment and control groups, (c) key outcomes measured, (d) data collection, and (e) estimation strategy.

### 5.1 Randomization

Our unit of randomization was the health facility: puskesmas in Indonesia and dispensaries in Tanzania. Our sample included 200 facilities in Indonesia and 153 facilities in Tanzania. Prior to randomization, we randomly selected one village in the catchment area served by each sample facility.^14^ In Tanzania, we also randomly selected a second village in the catchment area of 47 sample dispensaries, for a total of 200 villages. We then randomly assigned each health facility, and the one or two communities previously selected from its catchment area, to the treatment or control group.

Random assignment to the treatment group was stratified on a few key variables. In Indonesia, we stratified on province (Banten or South Sulawesi) and the proportion of women in the village who had delivered in a health facility (above or below the sample median). In Tanzania, we stratified on three binary characteristics: region (Dodoma or Tanga), the proportion of women in the village who had delivered in a health facility at baseline (above or below the sample median), and whether there were one or two sample villages in the catchment area of the health facility.

The T4D program was then offered in the randomly selected villages in the catchment areas of facilities assigned to the treatment group; the selected villages in the catchment area of facilities assigned to the control group constitute the counterfactual.^15^

### 5.2 Baseline equivalence

Before the program began, we verified balance between the treatment and control groups on variables used in stratifying as well as a host of other baseline characteristics. In Indonesia, the difference between the treatment and control group was statistically significant at the 5% significance level for only five of the 96 baseline variables tested (including key outcomes),^16^ which falls within the expected bounds of random or naturally occurring sample variation. Similarly, in Tanzania, the difference between treatment and control groups was statistically significant at the 5% significance level for only six of 112 variables.^17^ (For details, see Appendix A1.)

### 5.3 Outcomes

We focus on a set of primary outcomes pre-registered prior to endline data collection^18^: 1) utilization of maternal and newborn health services, 2) content of these health services, 3) child health outcomes, and 4) civic participation and perceptions of civic empowerment among communities. We also analyze a set of secondary outcomes that are relevant for understanding impact on the primary outcomes but are not used to assess the overall impact of the program^19^ (see Appendix A2 for outcome definitions).

With multiple outcomes, there is increased risk of over-rejecting the null hypothesis of no effects. We address this risk in two ways. First, following Casey, Glennerster, and Miguel (2012), we limit the number of outcomes by grouping related outcomes for content of care and civic participation into unweighted mean effects indices. In addition, we control the False Discovery Rate (FDR) using the two-stage linear step-up procedure in Benjamini, Krieger, and Yekutieli (2006) as implemented in Dupas, Huillery, and Seban (2018), which limits the expected proportion of rejections that are Type I errors.

Additionally, we analyze a range of intermediate outcomes to explore the mechanisms by which the activities participants planned in the meetings might have influenced their access to quality maternal and newborn health care service, and thereby influenced the primary outcomes. In particular, we explore a comprehensive set of characteristics spanning eleven dimensions of healthcare access and provision that might have been influenced by the types of activities participants in the meetings planned, and that correspond to the types of actions planned by the CRs (Table 2; see Appendix A3 for the definitions of the associated intermediate outcomes). We also explore whether women who recently gave birth were aware of more activities to improve access to quality maternal and newborn health care in villages in the treatment group.

**Table 2:** Activities planned by participants.

*Proportion of communities in which participants planned activities to:*	Both	Indonesia	Tanzania
Increase awareness, knowledge & improved community attitudes	93.5%	92.0%	95.0%
Improve facility access	71.0%	79.0%	63.0%
Increase ability to pay (including demand-side cost solutions)	45.0%	44.0%	46.0%
Improve information transparency (cost, opening hours, etc.)	39.0%	42.0%	36.0%
Improve attitude, effort, or trust of provider	36.0%	41.0%	31.0%
Pass by-laws, develop partnerships with traditional providers, or other approaches aimed at health service uptake	35.0%	16.0%	54.0%
Increase availability of drugs, supplies, other inputs	28.0%	45.0%	11.0%
Improve facility infrastructure	28.0%	32.0%	24.0%
Increase staff (midwife, doctor, etc.)	17.5%	16.0%	19.0%
Improve facility cleanliness	6.0%	10.0%	2.0%
Improve provider knowledge	1.0%	2.0%	0.0%
Other community improvements – e.g. general hygiene or cleaning campaigns, planting medicinal gardens, or digging community wells	9.0%	18.0%	0.0%

*Note:* Proportions are based on the activities participants planned across the program meetings.

Finally, we triangulate our analysis of these intermediate outcomes with qualitative interviews, observations and ethnographic studies, described in Section 6.

### 5.4 Data

We rely on seven data sources. The first is a survey of women who had given birth in the last year in sample villages at baseline and endline, which included questions related to our primary, secondary, and intermediate outcomes. At baseline, survey firms in Indonesia and Tanzania interviewed 5398 recent mothers (2398 in Indonesia and 3000 in Tanzania). A second sample of 6001 recent mothers in Indonesia and 6008 in Tanzania were interviewed for the endline survey, which was conducted a year and a half after the program ended and three years after the baseline. Respondents in each village were randomly sampled. Prior to data collection, survey teams conducted an extensive listing exercise with village and hamlet leaders, formal and informal healthcare providers, as well as other informants, to prepare a list of all women who had lived for at least six months in the village and had given birth in the prior 12 months.^20^ The listing and sampling process is described in Appendix A4.

We complement these household data with a survey of healthcare providers in the sample health facilities, using these data mainly to explore intermediate outcomes related to the quality and use of maternal and newborn health care services. To explore how the activities of participants might have influenced the quality and use of these services, we rely as well on qualitative data. First, we analyze the plans of activities that participants developed to try to improve their access to quality maternal and newborn health care.^21^ A year and a half after the last program meeting, former participants in all villages were invited to focus group discussions in which they were asked to recall the activities they planned and tried, challenges they encountered, and what if anything they thought had improved because of their efforts. An average of eight former participants in Indonesia and nine in Tanzania participated in these focus groups in each village.

In a sub-sample of villages in the treatment group—41 in Indonesia and 40 in Tanzania^22^—trained observers attended three of the program meetings and answered a series of questions about engagement, discussion, and decision-making in those meetings. In 41 in Indonesia and 24 in Tanzania, three participants were individually interviewed about how their activities went, as were the facilitator, the village head, the village midwife or someone from the health facility, several others with whom the three participants said they had engaged, and several other people randomly chosen in each community to verify what occurred and understand their perspective. These qualitative interviews, observations, and focus groups are further described in Appendix A4.

Finally, we rely on ethnographic studies by four scholars who lived in or near eight communities—two each in Banten, South Sulawesi, Tanga and Dodoma—for 6–9 months spanning the period before, during, and after the intervention, and observed health care in a third nearby community in the control group.^23^

### 5.5 Estimation strategy

Given that villages were randomly assigned to treatment and control groups, our basic method of estimating program impacts consists of comparing mean outcomes for the treatment and control groups of communities at endline, a year and a half after the program ended. We estimate the following regression equation:

(1) $$Y_{\mathrm{ijk}}=\beta_{0}+\beta_{1}{\mathrm{TREAT}}_{\mathrm{jk}}+{\beta_{2}STRATA}_{k}+\varepsilon_{\mathrm{ijk}},$$

where $Y_{\mathrm{ijk}}$ is the outcome of interest for mother/child *i* in village *j* in catchment area *k*; ${\mathrm{STRATA}}_{k}$ is a vector of dummy variables that indicate the randomization strata; and ${\mathrm{TREAT}}_{\mathrm{jk}}$ denotes treatment assignment. We cluster the error term ε at the health facility level, which is the level of treatment assignment. The coefficient $\beta_{1}$ represents the impact estimate.

For robustness, we also conduct a second set of regressions controlling for village-level averages in outcome variables at baseline ($Y_{j}^{0}$):^24^

(2) $$Y_{\mathrm{ijk}}=\beta_{0}+\beta_{1}{\mathrm{TREAT}}_{\mathrm{jk}}+{\beta_{2}STRATA}_{k}+\beta_{3}Y_{j}^{0}+\varepsilon_{\mathrm{ijk}}$$

Finally, we estimate heterogeneity of impact on the primary outcomes for three subgroups specified in the pre-analysis plan: province or region within each country; time since the program ended (6 months or less); and the existing quality of the health care system. We use Eq. (3) to estimate heterogeneity in impacts for the first two, region and time:

(3) $$Y_{\mathrm{ijk}}=\beta_{0}+\beta_{1}{\mathrm{TREAT}}_{\mathrm{jk}}+{\beta_{2}STRATA}_{k}+\beta_{4}{\mathrm{GROUP}}_{\mathrm{ij}}+\beta_{5}{\mathrm{TREAT}}_{\mathrm{jk}}\times {\mathrm{GROUP}}_{\mathrm{ij}}+\varepsilon_{\mathrm{ijk},}$$

where ${\mathrm{GROUP}}_{\mathrm{ij}}$ is a dummy variable denoting the subgroup of respondent *i* in village *j* (either province or region, or whether the respondent gave birth between 0 and 6 months prior to being interviewed at endline). ${\mathrm{TREAT}}_{\mathrm{jk}}\times {\mathrm{GROUP}}_{j}$ is an interaction between this dummy and the treatment dummy variable. To examine impact heterogeneity by variability in the quality of the existing health care system, we use a similar approach but include group dummy variables for baseline health care quality.^25^

## 6 Findings

In both Indonesia and Tanzania, we find that the core components of the T4D program were implemented in villages in the treatment group largely as designed, and that on average, the program had no statistically significant impacts on any pre-specified primary or secondary outcomes in either Indonesia or Tanzania. This section describes findings related to (a) implementation, (b) participation in the program, (c) impacts on primary and secondary outcomes, (d) impacts on intermediate outcomes, and (e) impacts on sub-groups.

### 6.1 Implementation

The program in Indonesia was rolled out in two asynchronous waves over seven months in 2015–2016. In Tanzania the program was administered in four overlapping waves over ten months in 2015–2016 (for details, see Transparency for Development Project Team, 2016b). The core components of the program appear to have been implemented with a high degree of fidelity to random assignment and to the design of planned meetings and other activities.^26^ In particular, program officers from partner organizations observed a selection of meetings directly and reviewed reports compiled by facilitators on each meeting, including information on who participated, which meetings they attended, and the activities they planned. In addition, external firms were hired to monitor the program in a third of the communities who were assigned to the treatment group and phone calls were made to their village heads to verify that the program had occurred there.

### 6.2 Participation in the program

Although participants in the program were unpaid and offered no additional resources with which to try to improve their access to quality maternal and newborn health care,^27^ our data suggest that program meetings occurred largely as planned in all villages in the treatment group and that participants generally stayed engaged throughout the program. In Indonesia, facilitators reported that an average of 12 of the 15–16 people whom they had invited attended the first meeting; meeting observations in a sample of communities in the treatment group suggest similar participation. (The most poorly attended had three participants, but in several communities more people wanted to attend than the facilitator had invited; the largest meeting had 21). The final meeting had an average of nine participants, ranging from one to 17. In Tanzania, attendance at the first meeting averaged 15, ranging from 12 to 16. At the final meeting the average number of participants was 12; the most poorly attended had three participants, and the most well-attended had 28 participants. Meeting observations also suggest that most participants engaged in the discussions rather than sitting quietly, and that in the majority of communities, participants frequently told local stories and offered examples of the more general topics under discussion, and generally conducted discussions and made decisions themselves rather than relying on the facilitator. In both countries, the majority of those who participated in the discussions were women.

Participants in these meetings planned a large number of activities to try to improve their maternal and newborn health care. Participants in the average community in Indonesia planned between three and 17 activities, with a median of seven; in Tanzania, they planned between two and eight actions, with a median of four. In total, participants in the program planned 1139 activities across the three months of meetings, 715 in Indonesia and 424 in Tanzania. In both countries, these activities were designed to alleviate problems with a wide range of issues, from cost of service to health facility accessibility to expectant mothers’ awareness of recommendations and best practices in seeking care. For example, some planned to visit pregnant women to talk to them about the importance of giving birth at a health facility; others to meet with health facility staff to discuss the availability of medicine, supplies, and the high cost of delivery; others to create lists of community members whose cars could be used to transport patients to health facilities for deliveries and treatment of other illnesses or injuries; and others to work with their community to repair roads to allow easier access to the health facility. Table 2 groups planned activities into 12 broad categories.

At the final program meeting, when facilitators asked participants which of their planned activities they had completed, they described 58% as complete (53% in Indonesia; 65% in Tanzania) and another 29% as ongoing (31% in Indonesia; 24% in Tanzania). Interviews in a sub-sample of communities suggest that the percentage of planned activities that were actually completed by the final program meeting may have been lower (36% in Indonesia; 52% in Tanzania). Nonetheless, both participants’ plans and interviews with participants and others in their community with whom they tried to engage suggest that participants attempted many of their planned activities.

### 6.3 Impact on primary and secondary outcomes

**Fig. 2:**
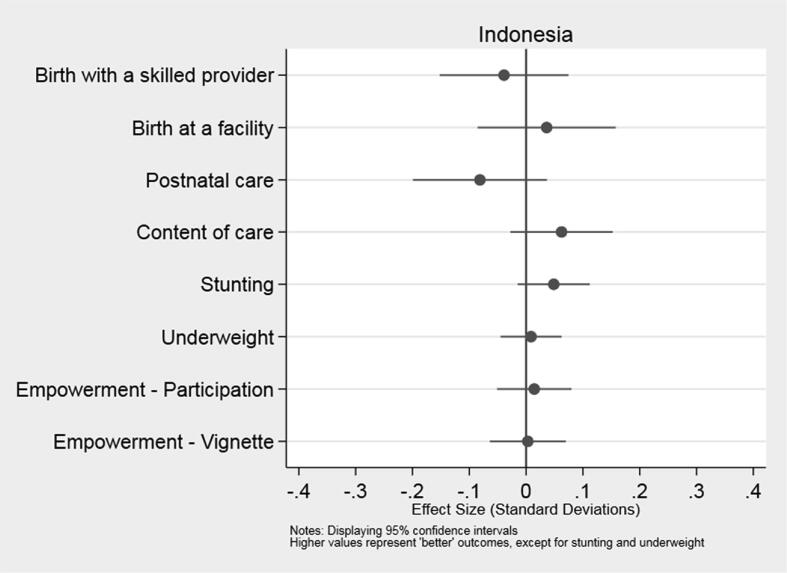
Impact of the T4D Program on Primary Outcomes in Indonesia.

**Fig. 3:**
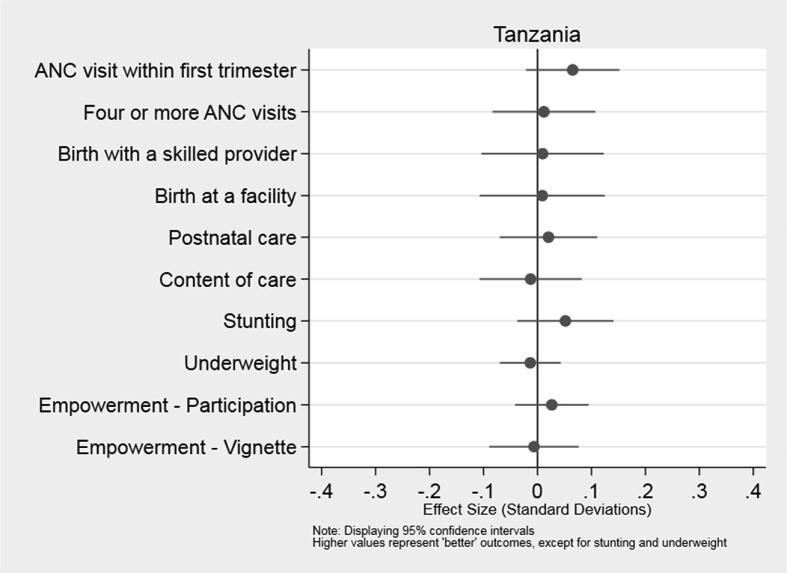
Impact of the T4D Program on Primary Outcomes in Tanzania.

In Fig. 2, Fig. 3, we present a summary of the T4D program’s impact on pre-specified primary outcomes in Indonesia and Tanzania, respectively. Estimates of effect sizes fall short of 0.1 standard deviations across all outcomes, indicating that the differences between the treatment and control groups were small in both countries. Moreover, the 95% confidence intervals include zero in every case. Hence, we cannot reject the null hypothesis of no effect for any of the primary outcomes: utilization and content of MNH services, infant height-for-age and weight-for-age, or civic participation and perceptions of empowerment among recently pregnant women in communities who were offered the program. Similarly, we find no statistically significant differences between the average village in treatment and control groups on any of the secondary outcomes in Indonesia and Tanzania (see Table 3). These conclusions remain unchanged when we estimate Eq. (2), controlling for the village average of the relevant outcome at baseline; see Appendix A5.

**Table 3:** Impact of T4D on Secondary Outcomes in Indonesia and Tanzania.

Indonesia						
	(1)	(2)	(3)	(4)	(5)	(6)
	Low birthweight	Maternal depression (K6 score)	Birth preparedness	Four or more ANC visits	First ANC visit within the first trimester	Content of Antenatal Care
Treatment	0.00449	−0.0882	0.0157	0.00587	−0.00931	−0.0161
	(0.00773)	(0.151)	(0.0727)	(0.0167)	(0.0175)	(0.0688)
Constant	0.0896***	18.28***	5.202***	0.875***	0.745***	5.979***
	(0.00820)	(0.151)	(0.0728)	(0.0177)	(0.0177)	(0.0657)
Observations	5423	5971	6001	5994	5911	6001

Control Mean	0.08	18.32	4.91	0.83	0.73	5.73
Tanzania						
Treatment	−0.00584	0.0752	−0.00235			
	(0.00611)	(0.182)	(0.0818)			
Constant	0.0521***	18.72***	5.661***			
	(0.00572)	(0.243)	(0.0849)			

Observations	6006	5859	6008			
Control Mean	0.05	18.11	5.04			

*Notes:* Robust standard errors clustered at the facility-level in parentheses. All regressions include strata-specific binary variables. Outcomes in columns (4)–(6) were included as part of the primary outcomes in Tanzania, and hence their impacts are not reported in this table. *** p < 0.01, ** p < 0.05, * p < 0.1

### 6.4 Impacts on Intermediate outcomes

We also use Eq. (1) to estimate the program’s impact on the intermediate outcomes. Overall, we conclude that the program did not affect intermediate outcomes in the average village in the treatment group in either Indonesia or Tanzania. Using the household and facility survey data, we analyze 106 intermediate outcomes in Indonesia, and find statistically significant differences at the 95% confidence level between treatment and control groups for nine (see Appendix A6 for detailed results), which is similar to what we would expect to see purely by chance. Indeed, when we control the false discovery rate, the adjusted p-values for these outcomes do not meet the conventional thresholds for statistical significance.

Our conclusions in Tanzania are similar. We analyze 126 intermediate outcomes from surveys of recently pregnant women and surveys and observations in health dispensaries, and find statistically significant differences at the 95% confidence level between treatment and control groups for eight (see Appendix A7 for detailed results). With 126 outcomes, six could be significant purely by chance, and as in Indonesia, the observed statistical significance of these eight outcomes in Tanzania does not survive correction for multiple hypotheses testing.

### 6.5 Sub-group analysis

As described in Section 5, we estimate heterogeneity of impact on the primary outcomes for three subgroups specified in the pre-analysis plan – province or region within each country; time since the program ended; and the existing quality of the health care system. Overall, we see no systematic discernible variation of impacts within any of these three sub-groups (see Appendix A8 for details).

## 7 Unpacking the lack of impacts

In this section, we use qualitative data on the program and participants’ response to it to explore possible explanations for the lack of statistically significant impacts. We first explore methodological factors that could explain the lack of impacts and conclude that these are unlikely. We then return to the framework we proposed in Fig. 1, using it along with qualitative evidence from interviews, focus groups and observations to trace the causal paths from participation to impacts. This helps us discard some explanations for the lack of average impacts and to offer evidence suggesting others that are more likely.

### 7.1 Methodological explanations

One possibility is that the analysis was statistically underpowered. The 95% confidence intervals of the impact estimates presented above suggest that this is unlikely. In Indonesia, these intervals imply an ability to rule out impacts of 0.1 standard deviations or more for five of the eight outcomes and impacts of 0.2 standard deviations or more for the remaining three. In Tanzania, the confidence intervals imply an ability to rule out impacts of 0.1 standard deviations or more for four of the 10 outcomes, impacts of 0.15 standard deviations or more for another four outcomes, and impacts of 0.2 standard deviations or more for the remaining two. In Appendix A9, we list the differences in primary outcomes that were detectable given our experimental design and sample size, and the findings of this more formal power analysis are also consistent with our conclusion that the analysis was sufficiently powered to detect relatively small effects for most outcomes in both Indonesia and Tanzania.

A second possibility is that too much or too little time had elapsed between the conclusion of the program and the endline survey a year and a half later. If participants made noticeable improvements to their access to quality care during or immediately after the program but these improvements faded over time, perhaps the care for a cohort of infants conceived closer to the program’s conclusion might have been more improved than the care for later cohorts. Our analysis suggests this is unlikely. If it were the case, we would have expected the sub-group analysis to indicate impacts of the program that were different for mothers who gave birth closer to the date of the survey (i.e. between 0 and 6 months) than those who gave birth later (i.e. between 6 and 12 months), but we observed no significant differences between these sub-groups. An analogous concern is that *too little* time had elapsed between the program and the endline surveys. Although we cannot rule out this possibility, endline surveys in both Indonesia and Tanzania commenced approximately 21 months after the completion of the first two program meetings, a period long enough for the birth of a new cohort of infants and to see most short- and medium-term effects of participants’ planned activities.

Overall, given the above evidence and identification strategy, we conclude that it is highly improbable that we failed to detect an average causal impact of the program for methodological reasons.

### 7.2 Exploring the causal paths from participation to impact

To further explore the lack of average effects from the T4D program, we use qualitative data to explore the causal pathways in the framework in Fig. 1. Section 6 (a)-(b) above describe observational and interview evidence that strongly suggests that in the average community, there were some people who were willing to participate in the meetings and to try at least some of the plans of activities that they had designed. In particular, the evidence suggests that the programs were implemented largely as designed in both Indonesia and Tanzania, and that participants in the program meetings planned a large number of activities (Column A of Fig. 1). Focus groups, interviews, observations of meetings, and ethnographic studies all indicate that many did in fact try out some of what they had planned (Column B). The remaining evidence described in Section 6 also suggests that these efforts did not improve maternal and newborn health care and outcomes in the average community in the treatment group (Columns C-E). The remainder of this section explores these missing links between columns B and C in the framework.

One possible reason is that there were significant secular improvements in maternal and newborn healthcare and outcomes in the years between the program and the endline surveys, which obscured the improvements that participants in the program were able to achieve. Section 4 noted that improving the health of pregnant women and infants was a priority concern both for the governments of Indonesia and Tanzania as well as for the international community during the time of the program. Overall, access to quality health care did improve in both treatment and control communities on several indicators.^28^ However, an environment of steady improvements is not a complete or satisfactory explanation of our findings. First, the improvements were not observed across every indicator (e.g., the proportion of underweight sample infants in Indonesia was quite similar at baseline and endline: 14% and 13%, respectively) and overall communities in Tanzania saw much less improvement than communities in Indonesia. Second, in nearly all communities where the program was offered, there were still people who thought their care could be even better and who were willing to try civic activities to improve it.

Instead, the evidence overall suggests that a more likely explanation for the lack of difference in intermediate outcomes between communities in the treatment and control groups (Column C) is that participants’ activities only rarely added measurably to efforts to improve maternal and newborn health care and outcomes that most communities were experiencing anyway.

First, in nearly all (93.5%) communities, participants’ planned activities included efforts to educate, inform, or “socialize” others in their community, suggesting that they believed that lack of knowledge was preventing more pregnant women from seeking care. In both Indonesia and Tanzania, these efforts typically focused on women of childbearing age and young families, and aimed to improve awareness of the importance of seeking maternal and newborn health services in health facilities before, during, and after birth: for example, the importance of seeking antenatal care and birth preparedness planning, the importance of giving birth at a facility or of seeking postnatal care services, or of men about accompanying their spouses to the health facility. Some went door-to-door to convey messages; others organized educational events involving providers from the health facility to speak with women of childbearing age; others asked midwives to integrate education into their monthly outreach services.

Yet these kinds of efforts were already common in both Indonesia and Tanzania. Despite their prevalence among planned activities, we find little evidence to suggest that these efforts added significantly to the large number of existing efforts in most communities to improve knowledge of, or attitudes toward, maternal and newborn health services and standard practices.^29^ In Indonesia, about two-thirds of expecting mothers were aware of an education or socialization campaign in their village in both the treatment and the control groups (row (2) of Table 4). In Tanzania, 44% of respondents in the treatment group were aware of these kinds of campaigns, only about 5.6 percentage points (0.1 standard deviations) more than respondents in the control group (row (2) of Table 5).

**Table 4:** Indonesia: Community awareness regarding potential health activities in their village.

Potential health activities	Treatment Mean	Control Mean	Difference	p-value	Effect Size	Sample Size
Total number of potential health activities	5.716	5.570	0.146	0.398	0.046	5999
*Proportion of respondents aware of –*						
Socialization campaign aimed at encouraging women to visit health facility	0.652	0.642	0.010	0.597	0.021	5811
Request for a new ambulance	0.272	0.239	0.033	0.230	0.078	5454
Attempts to improve the stock of drugs/equipment at the health facility	0.487	0.460	0.027	0.242	0.054	5064
Attempts to improve the attitude or performance of health facility staff	0.530	0.492	0.039*	0.056	0.077	5312
Public posting of the cost of service at the health facility	0.188	0.185	0.003	0.852	0.008	5755
Community members building or requesting a new health facility	0.238	0.251	−0.012	0.471	−0.029	5466
Attempts to improve health facility infrastructure	0.483	0.474	0.009	0.680	0.018	5518
Improvement to the road leading to the health facility	0.627	0.629	−0.003	0.910	−0.005	5811
Attempts to reduce the cost of mother and child health services	0.307	0.309	−0.002	0.919	−0.004	5600
Creation of a community savings group	0.069	0.044	0.0246*	0.072	0.120	5666
Improvements to the posyandu	0.678	0.652	0.026	0.225	0.054	5664
Community organized transportation to a health facility	0.085	0.087	−0.002	0.911	−0.005	5754
Hygiene or cleaning campaign	0.482	0.445	0.0374*	0.086	0.075	5769
Partnership between midwives and baby dukun	0.622	0.658	−0.035	0.153	−0.075	5577
Additional staff allocated to this village or the health facility	0.471	0.466	0.005	0.837	0.011	4959

Number of Respondents	3016	2985				
Number of villages	100	100				

*Notes:* Treatment means are regression adjusted. *** p < 0.01, ** p < 0.05, * p < 0.1.

**Table 5:** Tanzania: Community awareness regarding potential health activities in their village.

Potential health activities	Treatment Mean	Control Mean	Difference	p-value	Effect Size	Sample Size
Total number of potential health activities that respondent was aware of (ranging from 0 to 15)	4.679	4.247	0.432***	0.010	0.141	6003
*Proportion of respondents aware of –*						
Socialization campaign aimed at encouraging women to visit health facility	0.438	0.382	0.056**	0.010	0.114	5838
Creation of a new bylaw relating to mother and baby health	0.358	0.323	0.036	0.179	0.076	5820
Attempts to improve the stock of drugs/equipment at the health facility	0.289	0.286	0.003	0.877	0.007	5430
Attempts to improve the attitude or performance of health facility staff	0.275	0.265	0.010	0.612	0.022	5359
New complaint or suggestion box at the health facility	0.416	0.348	0.068**	0.025	0.143	5408
Community members building or requesting a new health facility	0.425	0.362	0.064*	0.054	0.132	5764
Attempts to improve health facility infrastructure	0.455	0.432	0.023	0.338	0.047	5778
Improvement to the road leading to the health facility	0.398	0.365	0.033	0.195	0.068	5880
New mobile clinic or other outreach services from the health facility	0.285	0.262	0.023	0.425	0.052	5910
Creation of a community savings group	0.185	0.159	0.025	0.159	0.069	5883
Construction of a placenta pit	0.474	0.440	0.033	0.241	0.067	5391
Registry of men who do not support their wives in accessing health services	0.062	0.054	0.009	0.304	0.038	5696
Creation of a maternity home for women to wait near the health facility	0.209	0.183	0.025	0.120	0.066	5877
Campaigns aimed at educating TBAs	0.382	0.356	0.026	0.239	0.055	5761
Additional staff allocated to the dispensary or health center	0.269	0.274	−0.005	0.807	−0.012	5772

Number of Respondents	2971	3037				
Number of villages	100	100				

*Notes:* Treatment means are regression adjusted. *** p < 0.01, ** p < 0.05, * p < 0.1.

Two-thirds of the activities that participants planned were not focused on education; they instead sought other improvements in their access to quality maternal and newborn health care services. But household survey data indicate that recently pregnant women in the treatment communities were by and large no more aware of these other kinds of activities in their communities than recently pregnant women in the control communities (Table 4, Table 5).

Moreover, the evidence suggests that most of these other kinds of activities did not succeed in achieving their goals. Ethnographic studies and interviews in a sample of villages in the treatment group with participants, those with whom they tried to engage as they pursued their activities, and other members of their communities, suggest that these activities were successful at achieving their goals in only about two-fifths of villages. In fewer still were participants later able to recall any tangible improvements in their access to quality maternal and newborn health care as a result of their efforts. During the endline focus group discussions in all villages in the treatment group (a year and a half after the program ended), participants were asked if they thought their activities had improved the health care available to mothers and infants in their communities. Participants in 41% of communities in Indonesia and 30% in Tanzania described achieving at least one tangible outcome from their efforts (Table 6): for example, a new ambulance, a generator, or other equipment for the health facility, a new health facility or easier access to an existing one, or healthcare staff who had come to reside in the village.^30^ The percentage of communities in which participants recalled achieving tangible improvements from at least two of their efforts—suggesting that their efforts may have led to broader improvements in their care rather than one-off successes—is still lower: 14% in Indonesia, and 4% in Tanzania.

**Table 6:** From plans to outcomes.

	(1)	(2)	(3)	(4)	(5)	(6)	(7)
	% villages that designed at least one non-education action	% villages that attempted at least one non-education action*	% of villages that completed at least one non-education action	% villages that completed at least one non-education action*	% villages where at least one non-education action was successful*	% villages where CRs recalled at least one tangible outcome 1.5 years later	% villages where CRs recalled at least two tangible outcomes 1.5 years later
Indonesia	*100%*	*93%*	*84%*	*59%*	*44%*	*41%*	*14%*
Tanzania	*100%*	*100%*	*91%*	*83%*	*46%*	*30%*	*4%*

*Notes:* * Estimates based on a sample of villages in the treatment group. Sources as follows: (1) Based on participants’ plans from all villages (columns 1 and 3); key informant interviews and ethnographic studies in 41 villages in Indonesia and 24 in Tanzania (columns 2, 4, and 5); and responses in endline focus group discussions with participants in all villages in the treatment group to the question “In the end, what was the outcome from this activity?” (columns 6 and 7). Minor discrepancies in interviews about planned activities led to dropping 11 activities from these proportions (5 in Tanzania, 6 in Indonesia).

Why did participants’ efforts not generate more of an impact on health outcomes? The diversity of villages and actions included in our sample makes it unlikely that there is a single explanation. Here we consider several factors that may jointly help to explain the disconnect.

It is possible that in many communities, participants or others in their communities simply did not think their maternal and newborn health care was sufficiently underperforming to justify more intensive efforts on their part to improve it. As noted, the general context turned out to be one in which some maternal and newborn health care indicators were improving substantially already, even without the efforts of participants in this program. Baseline surveys with recently pregnant women also suggest that the context was one in which maternal and newborn health care services were perceived by many to be performing well in many communities even before the program began. In 79% of the communities in the treatment group in Indonesia, more than three quarters of recently pregnant women told interviewers at baseline that the respect their provider had shown them during their most recent pregnancy was good or excellent; 68% said that the availability of drugs and equipment was good or excellent; and 79% that the overall quality of the care they had received was good or excellent. Experiences with quality care seemed less common in Tanzania, though in 67 of the 100 communities in the treatment group more than three quarters of recently pregnant women told interviewers that the respect the provider had showed them during their most recent pregnancy was good or excellent. In 23, more than three quarters of recently pregnant women said that the availability of drugs was good or excellent, and in 39 that the overall quality of care they had received was good or excellent.

Yet perceptions that maternal and newborn health care did not need further improvement are also not likely to be a sufficient explanation for our findings. Overall, the evidence suggests that in most communities, many people thought that their access to quality care could be improved further. At least three quarters of recently pregnant women told interviewers that *all* three of these aspects of their recent care—respect, availability of drugs and equipment, and overall quality of care—were good or excellent in only half of communities in Indonesia and 13 communities in Tanzania. The surveys also indicate that although recently pregnant women in most communities highly valued maternal and newborn health care, in most there were still some who had doubts about the importance of receiving care in a facility unless the mother was experiencing complications.^31^ Finally, as noted, in nearly all communities where the program was offered, there were still several who thought their care could be even better and were willing to participate in meetings to try to improve it. Indeed, observations of meetings in 81 communities in the treatment groups suggest that at least some participants seemed skeptical of the importance of improving their maternal and newborn health in only 10 (5 in Indonesia and 5 in Tanzania), and that in only 2, participants discussed no stories of local examples of problems with maternal and newborn health or the need to improve it.

Rather than simply a lack of perceived need for further improvement, we propose three further interrelated possibilities for why participants in most communities were not able to achieve more measurable average improvements in their access to high quality care.

First, several of the activities that participants tried would have had a weak, indirect, or no effect at all on health outcomes even if they were perfectly executed. For example, some planned activities involved cosmetic changes at the health facility, such as planting a garden or cleaning the premises, rather than more systemic changes with the potential to lead to measurable improvements in outcomes. Some activities were vague, one-off requests or complaints unlikely to lead anywhere without follow-up; many participants may have lacked the connections or means of approaching people in positions of authority, who might have helped them make more measurable or concrete improvements. Finally, in endline surveys of recently pregnant women, there is little correlation between many of the other aspects of care that participants in the T4D program tried to improve—such as whether staff were present or reachable, and the delivery room was clean—and whether or not mothers and children were healthier or used the facility more around birth.

A second possibility, strongly supported by the ethnographic studies, is the role of the history of other programs and projects in these places. Part of the similarity between treatment and control groups in community awareness of the various activities described in Table 4, Table 5 above may reflect this role of history: instead of coming up with new approaches tailored to their current circumstances, communities may have attempted activities with which they were perhaps already familiar. Memories of past programs may also have led people in these communities to have certain expectations of how these projects work – for instance, expectations of payment in return for participating – that led at least some to go along with the meetings the facilitator held and with initial attempts at the activities that they had designed, but then to “wait and see” before following through further.^32^

A final set of factors relate to the design of the program. A few key design features are worth reiterating. The program was designed to be: (i) non-prescriptive (it provided information to communities about maternal and newborn health care without suggesting any particular course of action to improve it), (ii) community-driven (it emphasized the importance of the community using their existing knowledge and capacities to understand and fix problems) and, (iii) offered no external resources (materials, funding, technical support, or relational resources). Finally, the program was designed to be relatively light-touch and scalable so that it could be offered consistently in diverse communities across large regions of two countries with varied economies, politics, and health systems. As our assessment of Columns A and B above reveals, a program designed with these goals did create the space for many communities to leverage local knowledge and collectively plan widely varied courses of action for improving their access to quality maternal and newborn health care services. However, the activities that participants in this limited program tried also appear in most cases to have had indirect or weak links to health outcomes or to have been insufficient to overcome some of the contextual factors cited above, at least over and above the improvements in health care that were already happening.

## 8 Conclusion

In recent years, transparency and accountability programs have been used increasingly in attempts to improve the responsiveness and effectiveness of public services in developing countries. In this paper, we evaluate the impact of a non-prescriptive, community-driven, health-focused transparency and accountability program in Indonesia and Tanzania. We find no evidence of average impact on healthcare, health, or perceptions of empowerment among recently pregnant women in communities who were offered this program.

Notwithstanding the many differences between the two countries where this program was offered – notably in terms of resource levels and healthcare provider choice – we also find substantial similarities in the reasons why the program did not have an average impact. Using qualitative evidence to trace a simple framework representing potential causal links between the program and health outcomes, we observe that the missing links in these causal chains were similar in both countries. We find that in both countries, participation in the program was substantial and sustained in most communities, and that participants planned and tried a wide variety of civic activities to try to improve their maternal and newborn health care. However, we also find that the efforts at informing and educating neighbors, which were common in both countries, were often small, localized or one-off community events that were insufficient to significantly increase knowledge or use of healthcare among pregnant women. Participants in only one-third of communities later recalled seeing their efforts translate into a tangible improvement, such as an ambulance or a new or improved facility or pharmacy, new staff, or more privacy. Future research from the mixed method Transparency for Development research initiative will explore several differences in the political and economic characteristics of communities that may partly explain differences in the experiences of participants and the efficacy of their efforts. We will detail evidence of several important additional similarities between the two countries in how people in communities offered the program responded, in whether they found the experience useful for improving their health care, in what else was important to them about participating, as well as in the broader historical dynamics in these communities and countries that influenced their experiences.

As reviewed in Section 2, our null findings fall along similar lines to what some other recent studies in the field have found (Raffler et al., 2019, Lieberman et al., 2014). However, they are in stark contrast to transparency and accountability studies that found significant improvements in outcomes, notably Björkman and Svensson (2009). While we can only speculate about the reasons underpinning the differences in the findings, we note two broad sets of factors that may be of interest for future research. The first is contextual differences in the times and places of the studies. These may include differences in level of development and the broader social, economic, and political environments that affected both the problems that led to low availability and use of high quality health care services as well as the potential for a transparency and accountability program to offer opportunities for measurable improvements. The second is differences in program design, among them differences in the length or intensity of each program, in the information or facilitation as well as relational, material, financial, or other resources or support each program offered, in the degree of standardization or adaptability in the program across communities, or in the selection of communities or participants who were offered it.

Overall, our findings and assessment in this paper highlight the complexity of the paths linking transparency and accountability programs to health outcomes, and lead us to be skeptical that one like the T4D program—a non-prescriptive, community-driven program that provided no additional resources—is sufficient, on average, to empower communities to measurably improve health outcomes across diverse contexts. It is possible that a transparency and accountability program that involved communities or participants differently, or that offered them more or more varied resources, facilitation, or support of other kinds to plan and realize improvements, would have made more of a difference in their capacities to navigate the paths from planning to achieving improvements in health outcomes. Future research into improving well-being or public services through community-led transparency and accountability programs would benefit from exploring these possibilities further.

## CRediT authorship contribution statement

**Jean Arkedis:** Conceptualization, Methodology, Formal analysis, Investigation, Writing - original draft, Writing - review & editing, Project administration, Funding acquisition. **Jessica Creighton:** Conceptualization, Methodology, Formal analysis, Investigation, Writing - original draft, Writing - review & editing, Project administration, Funding acquisition. **Akshay Dixit:** Conceptualization, Methodology, Formal analysis, Investigation, Writing - original draft, Writing - review & editing, Project administration, Funding acquisition. **Archon Fung:** Conceptualization, Methodology, Formal analysis, Investigation, Writing - original draft, Writing - review & editing, Project administration, Funding acquisition. **Stephen Kosack:** Conceptualization, Methodology, Formal analysis, Investigation, Writing - original draft, Writing - review & editing, Project administration, Funding acquisition. **Dan Levy:** Conceptualization, Methodology, Formal analysis, Investigation, Writing - original draft, Writing - review & editing, Project administration, Funding acquisition. **Courtney Tolmie:** Conceptualization, Methodology, Formal analysis, Investigation, Writing - original draft, Writing - review & editing, Project administration, Funding acquisition.

## Declaration of Competing Interest

The authors declare that they have no known competing financial interests or personal relationships that could have appeared to influence the work reported in this paper.

## Footnotes

1 Other experimental studies that have reported positive impacts include Banerjee et al. (2018), Andrabi et al. (2017), Fiala and Premand (2017), and Molina et al. (2017). See Section 2.

2 For example, Olken (2007), Banerjee et al. (2010) and Lieberman et al. (2014), Raffler et al. (2019). See Section 2.

3 The partner in Tanzania was the Tanzania country office for the Clinton Health Access Initiative (CHAI), an organization focused on improving health service delivery in a number of domains. The partner in Indonesia, PATTIRO, is a research and policy advocacy organization, which focuses on regional and local governance issues in a number of sectors.

4 Within the high-quality health-system framework proposed by The Lancet Global Health Commission (Kruk et al., 2018), our metrics relate to “Processes of care” and within that “Competent care and systems”.

5 Drawing on a wide variety of data sources provides important insights into the key processes that led to the observed null effects, addressing the black-box critique of impact evaluations (Rao & Woolcock, 2003; White, 2011; Deaton & Cartwright, 2018).

6 Fox, 2015; Olken & Pande, 2011; Joshi & Houtzager, 2012; McGee & Gaventa, 2011; Lieberman et al., 2014; Kosack & Fung, 2014.

7 Both contexts were marked by noteworthy secular improvements in healthcare over the course of the evaluation period. We discuss this further in Section 7.

8 In Section 7 we speculate on contextual conditions that may partly explain the effects we observe, and further explore these in forthcoming work.

9 For detailed baseline statistics, please refer to the baseline report (Transparency for Development Project Team, 2016a)

10 For detailed baseline statistics, please refer to the baseline report (Transparency for Development Project Team, 2016a)

11 The design process was modeled on “crawling the design space” in Pritchett, Samji, and Hammer (2013). See the Intervention Design report for details and differences from other transparency and accountability programs (Transparency for Development Project Team, 2016b).

12 This is a smaller group than in the community scorecards evaluated in Björkman and Svensson (2009) and Raffler et al. (2019), both of which involved an average of 100 invited participants. The group was invited through consultation with village leadership and other community members, the facilitator recruited community representatives who had an interest in working to improve their community’s health care and/or experience with difficulties accessing quality care, and to represent a broad swath of the village, particularly non-elites, including men, women, a range of ages, and those with a varying degree of previous leadership experience.

13 The only exception to this is that participants in Tanzania were offered compensation for attending the first two meetings, though not for any of the subsequent meetings.

14 This random selection was done after dropping urban communities (in Indonesia) and villages with a population of more than 10,000 or <1000 (in Tanzania). For the detailed village selection protocol, please refer to the baseline report (Transparency for Development Project Team, 2016a).

15 Note that the program was implemented at the village level, in the sampled village(s) associated with treatment health facilities.

16 The variables in Indonesia that were statistically significantly different between treatment and control villages were: 1) whether the mother received urine sample results during their ANC check; 2) whether the mother had ever had an ANC visit because of a complication; 3) the proportion of mothers paying for post-natal care; 4) whether government officials or political leaders had listened to and taken seriously a community proposal mothers recalled; 5) whether mothers or anyone in their households had participated in an information or election campaign in the past year.

17 The variables in Tanzania that were statistically significantly different between treatment and control villages were: 1) whether anyone in the household owned a bicycle; 2) a dummy variable for whether or not the mother felt they were properly informed of what was happening during her most recent visit to the health facility; 3) whether the mother gave birth in a private hospital; 4) whether the mother took a bicycle to the facility for delivery; 5) whether the mother had taken public transportation to the facility for delivery; 6) and the proportion of children who were underweight (by weight-for age ratios).

18 The Pre-Analysis Plan (AEA RCT #655) is available at https://www.socialscienceregistry.org/trials/655/.

19 Although the list of outcomes we explore in the two countries is generally similar, two differences are noteworthy. First, we analyze utilization and content of antenatal care as primary outcomes in Tanzania, but secondary outcomes in Indonesia. This is because baseline findings indicated that antenatal care outcomes in Indonesia were relatively high, and thus information on antenatal care was not included among the indicators that facilitators shared with community members during the first two meetings. Second, the components used to measure content of care (delivery, postpartum and postnatal) differed in the two countries based on their respective healthcare standards.

20 Note that because the sampling design involves interviewing women who had given birth in the 12 months prior to the surveys (baseline and endline), the two waves of data collection constitute repeated cross-sections, not a panel.

21 The program was designed for participants to make four plans: one each in the second program (planning) meeting and in the 30-, 60-, and 90-day follow up meetings. Facilitators were responsible for recording a copy of the social action plans developed during each of these meetings.

22 These were randomly selected, stratified on the same characteristics as those described in Section 5.

23 The eight villages were selected purposively from within the same national and regional stratifications as those offered the program as part of the broader RCT so that they were geographically close to each other to allow a single ethnographer to cover three villages.

24 Some primary outcome variables were not measured in the baseline survey. In these cases, the baseline control is either omitted, or we substitute a similar proxy variable.

25 We measure quality at baseline with a set of 12 indicators of minimum service delivery capacities and infrastructure that a health facility should have to be able to effectively provide maternal and newborn healthcare (including whether the facility has a specific room for deliveries; whether the delivery room has a clean bed; a clean floor; whether any dust/mold is observed in the room; whether the room has some form of auditory and/or visual privacy; whether the delivery room is well-ventilated or air-conditioned; whether biological/medical waste disposal is available; whether a separate bin for placenta disposal is available; whether water is available to flush in the toilet for patients who come to the facility to deliver; whether water is available to wash hands in the toilet; whether soap is available in the toilet; and whether MNH staff are present/reachable at all times). Given the observed variation in the data, we classified baseline quality of care into three categories in Indonesia (high, medium and low) and two categories in Tanzania (high and low). Regressions in Tanzania included a dummy for high relative to low quality; regressions in Indonesia included two group dummies (and their interactions with the treatment variable) for distinguishing high and medium from low quality.

26 We explore how the program was perceived and understood in a forthcoming book based on the ethnographic studies of the experiences of eight of the 200 communities who were offered the program.

27 As noted above, the only exception to this is that participants in Tanzania were offered compensation for attending the first two meetings, but not for any subsequent meeting.

28 For example, birth at a facility increased from baseline to endline substantially in the overall samples for both countries. It increased from 55% to 74% in Indonesia, and from 56% to 67% in Tanzania. Some of the other key primary outcomes also increased from baseline to endline, but the increases were much less substantial.

29 The ethnographic studies suggest that in some places, participants’ efforts may have had some effect on *how* education or socialization activities were run. In particular, their activities seemed to be more participatory, and less prescriptive in terms of telling community members what to do than the typical efforts led by others.

30 Considering that the broader environment was one of steadily improving maternal and newborn health care, perhaps some of these improvements would have happened anyway, without the efforts of the participants.

31 In almost all communities in both countries, a majority of recently pregnant women told interviewers that they thought it was important to seek regular maternal and newborn care in health clinics during pregnancy and birth. But in many communities there were still some recently pregnant women who expressed doubts. In Indonesia, in 51% of communities, more than a quarter told interviewers that it was not important for a woman who was experiencing no complications with a pregnancy and who had already had a baby without complications to seek antenatal care. In 52% of communities, more than a quarter told interviewers that it is fine for a woman in labor to wait to go to the health facility until she is having complications, and in 46% more than a quarter told interviewers that it is just as safe to deliver at home with a traditional birth attendant as to deliver in a facility. At least one recently pregnant woman offered these views to interviewers in 92%, 93%, and 86% of communities respectively.

32 A forthcoming book of ethnographic studies explores the role of history and memory in participants’ experiences of this program.

## References

[b0005] Andrabi T., Das J., Khwaja A.I. (2017). Report cards: The impact of providing school and child test scores on educational markets. American Economic Review.

[b0010] Banerjee A.V., Banerji R., Duflo E., Glennerster R., Khemani S. (2010). Pitfalls of participatory programs: Evidence from a randomized evaluation in education in India. American Economic Journal: Economic Policy.

[b0015] Banerjee A., Hanna R., Kyle J., Olken B.A., Sumarto S. (2018). Tangible information and citizen empowerment: Identification cards and food subsidy programs in Indonesia. Journal of Political Economy.

[b0020] Benjamini Y., Krieger A.M., Yekutieli D. (2006). Adaptive linear step-up procedures that control the false discovery rate. Biometrika.

[b0025] Björkman M., Svensson J. (2009). Power to the people: Evidence from a randomized field experiment on community-based monitoring in Uganda. The Quarterly Journal of Economics.

[b0030] Casey K., Glennerster R., Miguel E. (2012). Reshaping institutions: Evidence on aid impacts using a preanalysis plan. The Quarterly Journal of Economics.

[b0035] Deaton A., Cartwright N. (2018). Understanding and misunderstanding randomized controlled trials. Social Science & Medicine.

[b0040] Dicker D., Nguyen G., Abate D., Abate K.H., Abay S.M., Abbafati C., Abdelalim A. (2018). Global, regional, and national age-sex-specific mortality and life expectancy, 1950–2017: A systematic analysis for the Global Burden of Disease Study 2017. The Lancet.

[b0045] Dupas P., Huillery E., Seban J. (2018). Risk information, risk salience, and adolescent sexual behavior: Experimental evidence from Cameroon. Journal of Economic Behavior & Organization.

[b0050] Fiala, N., & Premand, P. (2017). Social accountability and service delivery: Evidence from a large-scale experiment in Uganda. Mimeo.

[b0055] Fox J.A. (2007). Accountability politics: Power and voice in rural Mexico.

[b0060] Fox J.A. (2015). Social accountability: What does the evidence really say?. World Development.

[b0065] Freedom House (2018a). Freedom in the World 2018 – Indonesia Country Report. Retrieved from: <https://freedomhouse.org/country/indonesia/freedom-world/2018>. (Accessed January 21, 2019).

[b0070] Freedom House (2018b). Freedom in the World 2018 – Tanzania Country Report. Retrieved from: <https://freedomhouse.org/country/tanzania/freedom-world/2018>. (Accessed January 21, 2019).

[b0075] Glennerster, R. (2005). Can information catalyze reform? Sierra Leone and rural India. Abdul Latif Jameel Poverty Action Lab, MIT.

[b0080] Hilber A., Blake C., Bohle L., Bandali S., Agbon E., Hulton L. (2016). Strengthening accountability for improved maternal and newborn health: A mapping of studies in Sub-Saharan Africa. International Journal of Gynecology & Obstetrics.

[b0085] Holland, J., & Schatz, F. (2016). Macro evaluation of DFID’s policy frame for empowerment and accountability. Retrieved from: <https://itad.com/wp-content/uploads/2017/06/EA-Macro-Evaluation-Technical-report-Dec16-FINAL.pdf>. (Accessed February 6, 2018).

[b0090] Joshi A. (2010). Review of impact and effectiveness of transparency and accountability initiatives: Annex 1 service delivery. Transparency and Accountability Initiative.

[b0095] Joshi A., Houtzager P.P. (2012). Widgets or watchdogs? Conceptual explorations in social accountability. Public Management Review.

[b0100] Keefer P., Khemani S. (2014). Mass media and public education: The effects of access to community radio in Benin. Journal of Development Economics.

[b0105] Kosack S., Fung A. (2014). Does transparency improve governance?. Annual Review of Political Science.

[b0110] Kruk M.E., Gage A.D., Arsenault C., Jordan K., Leslie H.H., Roder-DeWan S., English M. (2018). High-quality health systems in the Sustainable Development Goals era: Time for a revolution. The Lancet Global Health.

[b0115] Lieberman E.S., Posner D.N., Tsai L.L. (2014). Does information lead to more active citizenship? Evidence from an education intervention in rural Kenya. World Development.

[b0120] Mansuri G., Rao V. (2012). Localizing development: Does participation work?.

[b0125] McGee R., Gaventa J. (2011). Shifting power? Assessing the impact of transparency and accountability initiatives. IDS Working Papers.

[b0130] Molina E., Carella L., Pacheco A., Cruces G., Gasparini L. (2017). Community monitoring interventions to curb corruption and increase access and quality in service delivery: A systematic review. Journal of Development Effectiveness.

[b0135] Olken B.A. (2007). Monitoring corruption: Evidence from a field experiment in Indonesia. Journal of Political Economy.

[b0140] Olken, B., & Pande, R. (2011). Governance review paper: JPAL governance initiative. Abdul Latif Jameel Poverty Action Lab, MIT.

[b0145] Pritchett, L., Samji, S., & Hammer, J. S. (2013). It's all about MeE: Using Structured Experiential Learning ('e') to crawl the design space. Center for Global Development working paper (322).

[b0150] Raffler P., Posner D.N., Parkerson D. (2019). The weakness of bottom-up accountability: Experimental evidence from the Ugandan health sector.

[b0155] Rao, V., & Woolcock, M. (2003). Integrating qualitative and quantitative approaches in program evaluation. The impact of economic policies on poverty and income distribution: Evaluation techniques and tools (pp. 65–190).

[b0160] Reinikka R., Svensson J. (2005). Fighting corruption to improve schooling: Evidence from a newspaper campaign in Uganda. Journal of the European Economic Association.

[b0165] Reinikka R., Svensson J. (2011). The power of information in public services: Evidence from education in Uganda. Journal of Public Economics.

[b0170] Schaaf M., Topp S., Ngulube M. (2017). From favours to entitlements: Community voice and action and health service quality in Zambia. Health Policy and Planning.

[b0175] Transparency for Development Project Team (2016a). Baseline Report. Retrieved from: <https://ash.harvard.edu/files/ash/files/baseline_report.pdf>. (Accessed January 10, 2019).

[b0180] Transparency for Development Project Team (2016b). Transparency for Development Intervention Design. Retrieved from: <https://ash.harvard.edu/files/ash/files/intervention_design_0.pdf>. (Accessed January 10, 2019).

[b0185] White H. (2011). Achieving high-quality impact evaluation design through mixed methods: The case of infrastructure. Journal of Development Effectiveness.

[b0190] White, H., Menon, R., & Waddington, H. (2018). Community-driven development: Does it build social cohesion or infrastructure? A mixed-method evidence synthesis.World Bank (2017). GDP per capita (current US$) from World Bank national accounts data, and OECD National Accounts data files. Retrieved from: <https://data.worldbank.org/indicator/NY.GDP.PCAP.CD>. (Accessed January 9, 2019).

[b0195] You D., Hug L., Ejdemyr S., Idele P., Hogan D., Mathers C., Alkema L. (2015). Global, regional, and national levels and trends in under-5 mortality between 1990 and 2015, with scenario-based projections to 2030: A systematic analysis by the UN Inter-agency Group for Child Mortality Estimation. The Lancet.

