# Supplementary material for: Can transparency and accountability programs improve health? Experimental evidence from Indonesia and Tanzania
Source: World Dev. 2021 Jun;142:105369. doi: 10.1016/j.worlddev.2020.105369 (PMC8085768; doi:10.1016/j.worlddev.2020.105369)
Supplement: Supplementary data 1 [file mmc1.docx]

**Appendix A1. Balance Tables**

**Table A1.1. Indonesia**

| Balance Tables for Baseline Variables (Indonesia) | | |
| --- | --- | --- |
| Indicator | **Treatment Group** | **Control Group** |
| **Antenatal Care** | | |
| Care During Pregnancy |  |  |
| Had at least one ANC visit | 0.988 | 0.984 |
| Had ANC visit within 3 months of pregnancy^[[1]](#footnote-1)^ | 0.698 | 0.697 |
| Had 4 or more ANC visits ^[[2]](#footnote-2)^ | 0.869 | 0.873 |
| Woman visited multiple facilities to receive ANC | 0.614 | 0.636 |
| Woman visited clinic due to complications (%) | 0.201 | 0.264*** |
| ANC Content/quality index: |  |  |
| Average number of Iron tablets taken (for women who were given tablets, even if 0 taken) | 68.367 | 64.736 |
| Received Iron Tablets/ Syrup (not necessarily during ANC) | 0.951 | 0.932 |
| Blood sample taken | 0.362 | 0.380 |
| Received results of blood test | 0.878 | 0.881 |
| Urine sample taken | 0.502 | 0.520 |
| Received result of urine test | 0.818 | 0.877** |
| Blood pressure measured | 0.981 | 0.985 |
| ANC Care |  |  |
| Mother has ANC card | 0.783 | 0.794 |
| **Delivery Care** | | |
| Delivered baby at: |  |  |
| Pustu | 0.033 | 0.037 |
| Puskesmas | 0.143 | 0.171 |
| Pokesdes/Polindes/TKD | 0.045 | 0.054 |
| Hospital (Public) | 0.162 | 0.149 |
| Hospital (Private) | 0.023 | 0.029 |
| Doctor private practice | 0.003 | 0.002 |
| Midwife Private Practice | 0.123 | 0.102 |
| Birth Clinic | 0.012 | 0.010 |
| Own House/ Other's home | 0.449 | 0.443 |
| Assisted by village midwife | 0.008 | 0.003 |
| Other | 0.001 | 0.001 |
| Level of Care at Delivery |  |  |
| Delivered with skilled attendant (%)^[[3]](#footnote-3)^ | 0.778 | 0.796 |
| Highest skilled personnel assisting at birth: Assisted by Dukun | 0.168 | 0.144 |
| Respondent rated drugs available at delivery as excellent or good | 0.847 | 0.844 |
| Baby born by Caesarean | 0.121 | 0.103 |
| **Post-Partum Care** | | |
| Post-Partum Care - Mother |  |  |
| Received checks in physical and counselling categories, with a medical professional within 7 days | 0.538 | 0.548 |
| Received counselling on at least 2 topics from medical professional within 7 days | 0.583 | 0.575 |
| Received at least 2 physical checks for danger signs from medical professional within 7 days | 0.735 | 0.761 |
| Received at least one supplement from a medical professional within 7 days | 0.709 | 0.736 |
| Mothers reporting they stayed 24 hours or longer in facility after birth | 0.670 | 0.642 |
| Mother attended health facility for herself for any reason one or more times in past 3 months | 0.435 | 0.462 |
| Postnatal Care - Baby |  |  |
| Received at least 1 supplement with a skilled attendant within 7 days | 0.601 | 0.582 |
| Received required vaccinations with a skilled attendant within 7 days | 0.672 | 0.709 |
| Received 2 or more physical checks for danger signs with a skilled attendant within 7 days | 0.800 | 0.813 |
| Received a number of checks specified in all categories with a skilled attendant within 7 days^[[4]](#footnote-4)^ | 0.525 | 0.509 |
| Mother attended health facility for child for any reason one or more times in past 3 months | 0.632 | 0.637 |
| **Perceptions of quality from patient's experience and interaction with the provider** | | |
| Respondent's satisfaction with last visit to polindas |  |  |
| Visited polindas in last 3 months | 0.694 | 0.694 |
| Believes polindas health officer would try to make improvement in response to complaint | 0.754 | 0.764 |
| Overall experience of facility, “good” or “excellent” | 0.862 | 0.862 |
| Respect shown by provider during recent visit, “very satisfied” or “satisfied” | 0.875 | 0.875 |
| Trust in providers, “good” or “excellent” | 0.895 | 0.895 |
| Cleanliness of facility, “good” or “excellent” (recent general visit) | 0.485 | 0.485 |
| Availability of drugs, “good” or “excellent” (recent general visit) | 0.785 | 0.785 |
| Wait time during recent visit, “very satisfied” or “satisfied” | 0.833 | 0.833 |
| Respondent's satisfaction with last visit to puskesmas |  |  |
| Visited puskesmas in last 3 months | 0.667 | 0.667 |
| Believes puskesmas health officer would try to make improvement in response to complaint | 0.723 | 0.717 |
| Overall experience of facility, “good” or “excellent” | 0.875 | 0.875 |
| Respect shown by provider during recent visit, “very satisfied” or “satisfied” | 0.883 | 0.883 |
| Trust in providers, “good” or “excellent” | 0.912 | 0.912 |
| Cleanliness of facility, “good” or “excellent” (recent general visit) | 0.188 | 0.188* |
| Availability of drugs, “good” or “excellent” (recent general visit) | 0.798 | 0.798 |
| Wait time during recent visit, “very satisfied” or “satisfied” | 0.564 | 0.564 |
| Mother agrees that there is more than one facility available when care is needed | 0.828 | 0.805 |
| **Infant and Maternal Health Outcome in Sample Villages** | | |
| Infant mortality among most recent births^[[5]](#footnote-5)^ | 8.056 | 5.890 |
| Neonatal mortality among most recent births^[[6]](#footnote-6)^ | 1.691 | 1.378 |
| Baby Measurements |  |  |
| Baby was underweight (< 2.5kg)^[[7]](#footnote-7)^ | 0.066 | 0.068 |
| Weight for age (proportion >2 SD below mean)^[[8]](#footnote-8)^ | 0.148 | 0.173 |
| Length for age (proportion >2 SD below mean)^[[9]](#footnote-9)^ | 0.157 | 0.166 |
| **Issues Impacting Demand for MNH** | | |
| Existence of Barriers to Health Care Access |  |  |
| During most recent pregnancy, ever wanted to receive ANC care but not able to because of access difficulty (%) | 0.196 | 0.208 |
| Did not deliver in a facility because of access difficulty (%) (only those who didn't deliver in facility) | 0.288 | 0.255 |
| Form of transport taken to facility for delivery: |  |  |
| Ambulance | 0.081 | 0.076 |
| Public bus/angkot (Indonesia) | 0.044 | 0.038 |
| Hired private vehicle/motorcycle/ojek | 0.222 | 0.209 |
| Personal car/motorcycle | 0.368 | 0.355 |
| Relatives'/neighbors' vehicle | 0.202 | 0.227 |
| On foot | 0.029 | 0.053* |
| Other | 0.025 | 0.011 |
| Proportion of women travelling one hour or more for facility delivery | 0.202 | 0.232 |
| **Costs associated with MNH** | | |
| Costs associated with delivery of care |  |  |
| All mothers: Proportion of women paying for delivery | 0.781 | 0.798 |
| Women that paid any voluntary fee (%) (among those who paid) | 0.981 | 0.989 |
| Costs associated with post-partum care |  |  |
| Proportion of women paying for PNC (for mother & baby) (cash only) | 0.476 | 0.410** |
| Women that showed insurance document (%) | 0.311 | 0.346 |
| **Levels of Citizen Empowerment and Participation** | | |
| Respondent beliefs about government responsiveness: |  |  |
| Local Government Official 'often' or 'always' are responsive to citizens | 0.319 | 0.305 |
| Members of Parliament 'often' or 'always' are responsive to citizens | 0.113 | 0.099 |
| Empowerment and political action |  |  |
| Community has got together to petition government a few or many times in past year | 0.233 | 0.258 |
| Respondent was satisfied with Government response to latest petition | 0.636 | 0.761 |
| In past year, respondent or anyone in the household has: |  |  |
| Attended village/neighborhood council meeting, public hearing, discussion group | 0.364 | 0.417 |
| Alerted newspaper, radio or TV to a local problem | 0.012 | 0.009 |
| Met with a politician, called him/her, or sent a letter | 0.048 | 0.058 |
| Participated in a protest or demonstration | 0.029 | 0.024 |
| Participated in an information or election campaign | 0.125 | 0.167** |
| Notified police or court about a local problem | 0.013 | 0.019 |
| In most recent effort government officials/political leaders listened to, and took seriously, proposal | 0.654 | 0.836** |
| **Household Characteristics and Asset Index** | | |
| Proportion reporting ownership of: |  |  |
| Proportion reporting ownership of bicycle | 0.108 | 0.099 |
| Proportion reporting ownership of a telephone/mobile phone | 0.914 | 0.912 |
| Proportion reporting ownership of a motorcycle | 0.784 | 0.780 |
| Proportion reporting ownership of television | 0.862 | 0.844 |
| Large animals/ livestock | 0.186 | 0.179 |
| Mother’s highest level of school attended |  |  |
| Some or all of elementary school | 0.393 | 0.398 |
| Some or all of high school | 0.218 | 0.207 |
| Some or all of college (includes “academy”) | 0.110 | 0.117 |
| Reported woman is head of household (%) | 0.023 | 0.019 |
| House floor made out of marble/ceramic/brick/granite/cement | 0.608 | 0.662 |
| House roof made out of concrete/tiles/shingles/iron sheets | 0.842 | 0.866 |
| Households using electricity (%) | 0.988 | 0.985 |
| */**/***: Difference statistically significant at the 10%/5%/1% level. Control means are regression adjusted using the strata dummy variables as regressors. | | |

**Table A1.2. Tanzania**

| Balance Tables for Baseline Variables (Tanzania) | | |
| --- | --- | --- |
| **Indicator** | **Treatment Group** | **Control Group** |
| **Antenatal Care** | | |
| Care During Pregnancy |  |  |
| Had ANC visit within 3 months of pregnancy | 0.186 | 0.184 |
| Had 4 or more ANC visits ^[[10]](#footnote-10)^ | 0.439 | 0.428 |
| ANC content of care index | 0.717 | 0.741 |
| ANC Content/quality index: |  |  |
| Tested for HIV | 0.854 | 0.869 |
| Received Iron Tablets/ Syrup | 0.877 | 0.889 |
| Received malaria prevention drugs | 0.708 | 0.758* |
| Received drug for intestinal worms | 0.546 | 0.573 |
| Blood sample taken | 0.795 | 0.796 |
| Received results of blood test | 0.885 | 0.886 |
| Urine sample taken | 0.456 | 0.465 |
| Received result of urine test | 0.858 | 0.825 |
| Blood pressure measured | 0.638 | 0.634 |
| ANC Care |  |  |
| Mother has ANC card | 0.710 | 0.711 |
| Birth Planning |  |  |
| Planned where would have the baby before birth | 0.400 | 0.458 |
| Made a comprehensive birth plan^[[11]](#footnote-11)^ | 0.227 | 0.251 |
| **Delivery Care** | | |
| Delivered baby at: |  |  |
| Dispensary - Faith-based | 0.009 | 0.010 |
| Dispensary - Non-profit | 0.000 | 0.001 |
| Dispensary - Private | 0.007 | 0.005 |
| Dispensary - Public | 0.282 | 0.268 |
| Health Center - Faith-based | 0.002 | 0.003 |
| Health Center - Private | 0.000 | 0.001 |
| Health Center - Public | 0.072 | 0.070 |
| Hospital - Faith-based | 0.011 | 0.015 |
| Hospital - Private | 0.001 | 0.007** |
| Hospital - Public | 0.173 | 0.189 |
| Level of Care at Delivery |  |  |
| Delivered at facility (%) | 0.558 | 0.568 |
| Delivered with skilled attendant (%) | 0.559 | 0.569 |
| Highest skilled personnel assisting at birth: Assisted by TBA | 0.243 | 0.261 |
| Baby born by Caesarean | 0.043 | 0.053 |
| **Post-Partum Care** | | |
| Post-Partum Care - Mother |  |  |
| Received checks in physical and counselling categories, with a medical professional within 7 days | 0.101 | 0.096 |
| Received counselling on at least 2 topics from medical professional within 7 days | 0.159 | 0.175 |
| Received at least 2 physical checks for danger signs from medical professional within 7 days | 0.143 | 0.142 |
| Received at least one supplement from a medical professional within 7 days | 0.132 | 0.126 |
| Mother received any post-partum care with a skilled attendant within 7 days | 0.309 | 0.339 |
| Newborn received any post-partum care with a skilled attendant within 7 days | 0.619 | 0.635 |
| Received number of check specified in all three listed above (comprehensive) (skilled provider but not time bound) | 0.072 | 0.069 |
| Mothers reporting they stayed 24 hours or longer in facility after birth | 0.508 | 0.516 |
| Postnatal Care - Baby |  |  |
| Received required vaccinations with a skilled attendant within 7 days^[[12]](#footnote-12)^ | 0.181 | 0.204 |
| Received 2 or more physical checks for danger signs with a skilled attendant within 7 days^[[13]](#footnote-13)^ | 0.354 | 0.393 |
| Received number of checks specified in all categories with a skilled attendant within 7 days^[[14]](#footnote-14)^ | 0.126 | 0.147 |
| Received number of checks specified in all categories with a skilled attendant (not time bound)^[[15]](#footnote-15)^ | 0.346 | 0.377 |
| **Perceptions of quality from patient's experience and interaction with the provider** | | |
| Respondent's perception of how they are received at facility |  |  |
| Staff used bad language | 0.095 | 0.092 |
| Greeted or attended to promptly | 0.582 | 0.587 |
| Insufficient seating | 0.168 | 0.187 |
| Kept properly informed of what was happening | 0.112 | 0.147** |
| Not given appropriate options for treatment | 0.092 | 0.083 |
| Staff made sure they understood reason for visit | 0.291 | 0.314 |
| Respondent's satisfaction with last general visit to health facility |  |  |
| Cleanliness, good or excellent | 0.697 | 0.722 |
| Communication, good or excellent | 0.653 | 0.661 |
| Drug availability, good or excellent (during recent visit) | 0.496 | 0.498 |
| Waiting time, satisfactory/very satisfactory | 0.634 | 0.632 |
| Respect shown by provider, good or excellent | 0.713 | 0.741 |
| Degree of trust in medical staff, good or excellent^[[16]](#footnote-16)^ | 0.735 | 0.746 |
| Respondent rates overall quality of the facility during recent visit (not including delivery) as 'good' or 'excellent' | 0.607 | 0.609 |
| At least 4 (of 6) satisfaction measures good/excellent and overall satisfaction good/excellent | 0.602 | 0.607 |
| Mother agrees that there is more than one facility available when care is needed | 0.243 | 0.287 |
| **Infant and Maternal Health Outcome in Sample Villages** | | |
| Infant mortality among most recent births^[[17]](#footnote-17)^ | 8.844 | 5.006 |
| Neonatal mortality among most recent births^[[18]](#footnote-18)^ | 4.082 | 2.488 |
| Baby Measurements |  |  |
| Baby was underweight (< 2.5kg)^[[19]](#footnote-19)^ | 0.056 | 0.050 |
| Weight for age (proportion >2 SD below mean)^[[20]](#footnote-20)^ | 0.081 | 0.105** |
| Length for age (proportion >2 SD below mean)^[[21]](#footnote-21)^ | 0.266 | 0.263 |
| **Issues Impacting Demand for MNH** | | |
| Existence of Barriers to Health Care Access |  |  |
| During most recent pregnancy, ever wanted to receive ANC care but not able to because of access difficulty (%) | 0.078 | 0.079 |
| Did not deliver in a facility because of access difficulty (%) (only those who didn't deliver in facility) | 0.428 | 0.432 |
| During most recent pregnancy, ever wanted to receive PNC but not able to because of access difficulty (%) | 0.087 | 0.089 |
| Knowledge of mother |  |  |
| Beliefs about the appropriate minimum number of ANC visits: average | 2.726 | 2.907 |
| Believes pregnant woman should first seek ANC in the first trimester ^[[22]](#footnote-22)^ | 0.663 | 0.673 |
| Aware of need to seek care during delivery despite no complications | 0.889 | 0.914 |
| Believes it is not just as safe to deliver at home with TBA, as in a facility | 0.860 | 0.871 |
| Aware of need for ANC despite no complications with prior pregnancy | 0.885 | 0.914* |
| Number of women indicating 3 or more possible warning or danger signs that a woman is having a problem during pregnancy indicating that she should seek medical attention? | 0.388 | 0.422 |
| Form of transport taken to facility for delivery: |  |  |
| Ambulance | 0.007 | 0.008 |
| Bicycle | 0.004 | 0.016** |
| Hired car or motorbike | 0.419 | 0.395 |
| Ox Cart | 0.005 | 0.001 |
| Public Transport | 0.117 | 0.181** |
| Private car or motorbike | 0.098 | 0.100 |
| Truck | 0.001 | 0.003 |
| On foot | 0.369 | 0.332 |
| Distance to nearest health facility from village (km)^[[23]](#footnote-23)^ | 2.500 | 1.949 |
| Proportion of women travelling one hour or more for facility delivery | 0.479 | 0.465 |
| **Costs associated with MNH** | | |
| Costs associated with antenatal care |  |  |
| Women paid something for ANC visits at facility | 0.226 | 0.250 |
| Costs associated with delivery of care |  |  |
| Facility births: Proportion of women paying for delivery | 0.474 | 0.512 |
| TBA/Dukun Fees paid for: Bribe/gift/thanks | 0.268 | 0.258 |
| Costs associated with post-partum care |  |  |
| Proportion of women paying for PNC (for mother & baby) (cash only) | 0.172 | 0.158 |
| **Levels of Citizen Empowerment and Participation** | | |
| Respondent beliefs about ability to change life: |  |  |
| Totally or mostly able to change life | 0.664 | 0.666 |
| Respondent beliefs about responsiveness of health workers |  |  |
| Health officer would try to make improvement in response to complaint | 0.720 | 0.705 |
| Respondent Beliefs about Government Responsiveness: |  |  |
| Local Government Official 'often' or 'always' are responsive to citizens | 0.278 | 0.248 |
| Members of Parliament 'often' or 'always' are responsive to citizens | 0.065 | 0.063 |
| In past year, respondent or anyone in the household has: |  |  |
| Alerted newspaper, radio or TV to a local problem | 0.013 | 0.006* |
| Attended village/neighborhood council meeting, public hearing, discussion group | 0.580 | 0.580 |
| Met with a politician, called him/her, or sent a letter | 0.108 | 0.100 |
| Notified police or court about a local problem | 0.011 | 0.013 |
| Participated in an information or election campaign | 0.219 | 0.179* |
| Participated in a protest or demonstration | 0.122 | 0.094* |
| Participation Index | 0.413 | 0.416 |
| Empowerment and Political Action |  |  |
| In past year, respondent has participated in efforts to petition government a few or many times | 0.337 | 0.353 |
| In past year, community has got together to petition government a few or many times | 0.239 | 0.265 |
| Respondent was satisfied or very satisfied with Government response to latest petition | 0.406 | 0.390 |
| In most recent effort government officials/political leaders listened to, and took seriously, proposal | 0.542 | 0.518 |
| **Household Characteristics and Asset Index** | | |
| Proportion reporting ownership of: |  |  |
| Bicycle | 0.223 | 0.291** |
| Mobile telephone | 0.704 | 0.728 |
| Motorcycle | 0.094 | 0.100 |
| Television | 0.037 | 0.039 |
| Anyone in household owns large animals/livestock | 0.425 | 0.444 |
| Head of Household characteristics |  |  |
| Head of household attended any secondary school | 0.122 | 0.127 |
| Head of household completed any sort of degree/ diploma | 0.001 | 0.002 |
| Reported woman is head of household (%) | 0.126 | 0.135 |
| Reported Religion of mother: |  |  |
| Christianity | 0.479 | 0.412 |
| Islam | 0.516 | 0.582 |
| House floor made out of cement/concrete/cement blocks/stones/tiles/slates | 0.159 | 0.171 |
| House roof made out of metal | 0.582 | 0.617 |
| **Mental health of Mothers** | | |
| At or above 13 on K6 index (optimal cut point for US) | 0.149 | 0.153 |
| */**/***: Difference statistically significant at the 10%/5%/1% level. Control means are regression adjusted using the strata dummy variables as regressors. | | |

**Appendix A2. Primary and Secondary Outcomes**

**Table A2.1. Indonesia**

| **Category** | **Research Question** | **Outcome** | **Definition** |
| --- | --- | --- | --- |
| Primary | Utilization of MNH services | Delivery with a skilled birth attendant | Whether the respondent delivered with a skilled birth attendant. |
| Primary | Utilization of MNH services | Delivery at a health facility | Whether the respondent delivered at a health facility. |
| Primary | Utilization of MNH services | Post-partum care (mother) & Post-natal care (newborn) | Postpartum care – Whether the respondent received at least one post-partum check with a skilled attendant, after leaving the facility and within 7 days of giving birth.  Postnatal care – Whether the newborn received at least one post-natal check with a skilled attendant, after leaving the facility and within 7 days of birth.  Postpartum and postnatal care utilization combined by creating a single binary variable on whether **both** the mother and infant received checks. |
| The three content of care outcomes – delivery, postpartum, and postnatal content of care – were combined into unweighted mean effects index (as described in Section V of the paper), and treated as one single outcome on content of care. | | | |
| Primary | Content of MNH services | Delivery content of care | Number of delivery content of care components received by the respondent:   1. Initiation of breastfeeding within one hour of birth 2. Baby wrapped to mother (skin-to-skin contact) within 30 minutes of birth 3. Delayed bathing for 6 hours - deliveries at home only 4. [Oxytocin] injection right after delivery (after the baby but before the placenta) 5. Uterine massage after delivery of placenta - applicable for vaginal delivery only 6. Clean and dry cord care |
| Primary | Content of MNH services | Postpartum content of care (mother) | Number of postpartum content of care components received by the respondent:  Physical checks of the mother to treat complications that arise from delivery   1. Blood pressure 2. Checked breasts 3. Check for bleeding 4. Examine perineum   Advice to mothers on how to care for themselves and their children   1. Danger signs for newborns 2. Danger signs for mothers 3. Breastfeeding 4. Family planning/contraception   Provision of vitamins and supplements   1. Vitamin A |
| Primary | Content of MNH services | Postnatal content of care (newborn) | Number of postnatal content of care components received by the infant:  Physical checks of the newborn to treat complications that arise from delivery   1. Baby weighed 2. Body examined for danger signs (generally examined/looked at baby's body) 3. Checked cord   Provision of recommended vaccines   1. Polio 2. Hepatitis B (HB0) 3. BCG 4. DPT-HB   Provision of recommended vitamins or supplements   1. Vitamin K1 2. Eye cream |
| Primary | Health outcomes | Weight-for-age | Weight-for-age z-score. Whether the infant is below 2 standard deviations from the median WHO Child Growth Standards. |
| Primary | Health outcomes | Height-for-age | Height-for-age z-score. Whether the infant is below 2 standard deviations from the median WHO Child Growth Standards. |
| Primary | Civic participation | Participation | Index of activities associated with empowerment and efficacy. The following outcomes were combined into an unweighted mean effects index as described in Section V of the paper.  1) Whether the respondent reported that she (or a household member) participated in communal activities over the previous 12 months, in which people came together to work for the benefit of the community.  2) Whether the respondent reported that over the previous 12 months, people in her neighborhood or village had gotten together to petition government officials or political leaders for something benefiting the community.  3) Whether the respondent reported that she (or a household member) had done at least one of the following in the past 12 months –   - attended a village or neighborhood council meeting, public hearing, discussion group - met with a politician, called him/her, or sent a letter - participated in a protest or demonstration - participated in an information or election campaign - alerted newspaper, radio or TV to a local problem - notified police or court about a local problem - online activism (such as reporting problems on Facebook, Twitter, etc.) |
| Primary | Empowerment | Perceptions of empowerment | The perception of the respondent about her power to make important decisions and take actions that improve life in her village, for herself and others. This was assessed on a 4-point scale, where 1 means being totally unable to improve life in this village, and 4 means having full control to make important decisions and actions to improve life in this village. |
| Secondary | Utilization of MNH services | Four or more ANC visits | Whether the respondent attended four or more antenatal care visits with a skilled provider. |
| Secondary | Utilization of MNH services | First ANC visit within the first trimester | Whether the respondent had a first antenatal care visit within the first 13 weeks of pregnancy with a skilled provider. |
| Secondary | Utilization of MNH services | Birth preparedness | Number of birth preparedness activities conducted by the respondent:   1. Where to deliver the baby 2. Who will assist with the birth 3. Transportation to place of delivery 4. Payment for delivery 5. Identification of a compatible blood donor 6. Support to look after children while away (for women with childcare responsibilities only) 7. Support to look after the home while away |
| Secondary | Content of MNH services | Antenatal content of care | Number of antenatal content of care components received by the respondent during one or more antenatal care visits:   1. Iron tablets or syrup 2. Blood pressure measurement 3. Urine sample 4. Tetanus toxoid injection 5. Informed of signs of pregnancy complications 6. Counseled on birth preparedness planning 7. Counseled on nutrition |
| Secondary | Health outcomes | Birth weight | Whether the infant has a birth weight less than 2500g |
| Secondary | Health outcomes | Maternal depression | Respondent’s score on the Kessler Psychological Distress Scale (K6), measured by converting the K6 to a 0-24 scale (each of the six questions coded 0-4 and summed), with 13+ indicating SMI. |

**Table A2.2. Tanzania**

| **Category** | **Research Question** | **Outcome** | **Definition** |
| --- | --- | --- | --- |
| Primary | Utilization of MNH services | Four or more ANC visits | Whether the respondent attended four or more antenatal care visits with a skilled provider. |
| Primary | Utilization of MNH services | First ANC visit within the first trimester | Whether the respondent had a first antenatal care visit within the first 13 weeks of pregnancy with a skilled provider. |
| Primary | Utilization of MNH services | Delivery with a skilled birth attendant | Whether the respondent delivered with a skilled birth attendant. |
| Primary | Utilization of MNH services | Delivery at a health facility | Whether the respondent delivered at a health facility. |
| Primary | Utilization of MNH services | Post-partum care (mother) & Post-natal care (newborn) | Postpartum care – Whether the respondent received at least one post-partum check with a skilled attendant, after leaving the facility and within 7 days of giving birth.  Postnatal care – Whether the newborn received at least one post-natal check with a skilled attendant, after leaving the facility and within 7 days of birth.  Postpartum and postnatal care utilization combined by creating a single binary variable on whether **both** the mother and infant received checks. |
| The four content of care outcomes – antenatal, delivery, postpartum, and postnatal content of care – were combined into unweighted mean effects index (as described in Section V of the paper), and treated as one single outcome on content of care. | | | |
| Primary | Content of MNH services | Antenatal content of care | Number of antenatal content of care components received by the respondent during one or more antenatal care visits:   1. Blood pressure 2. Urine sample 3. Blood sample 4. HIV testing 5. Tetanus toxoid injection 6. Counseling on nutrition 7. Iron tablets/syrup 8. Medication for deworming 9. Medication to prevent malaria 10. Counseled on birth preparedness planning 11. Counseled on nutrition |
| Primary | Content of MNH services | Delivery content of care | Number of delivery content of care components received by the respondent:   1. Initiation of breastfeeding within one hour of birth 2. Baby wrapped to mother (skin-to-skin contact) within 30 minutes of birth 3. Delayed bathing for 6 hours - deliveries at home only 4. [Oxytocin] injection right after delivery (after the baby but before the placenta) 5. Uterine massage after delivery of placenta - applicable for vaginal delivery only 6. Clean and dry cord care |
| Primary | Content of MNH services | Postpartum content of care (mother) | Number of postpartum content of care components received by the respondent:  Physical checks of the mother to treat complications that arise from delivery   1. Blood pressure 2. Checked breasts 3. Check for bleeding 4. Examine perineum   Advice to mothers on how to care for themselves and their children   1. Danger signs for newborns 2. Danger signs for mothers 3. Breastfeeding 4. Family planning/contraception   Provision of vitamins and supplements   1. Vitamin A |
| Primary | Content of MNH services | Postnatal content of care (newborn) | Number of postnatal content of care components received by the infant:  Physical checks of the newborn to treat complications that arise from delivery   1. Baby weighed 2. Body examined for danger signs (generally examined/looked at baby's body) 3. Checked cord   Provision of recommended vaccines   1. Polio 2. BCG 3. DPT-HB |
| Primary | Health outcomes | Weight-for-age | Weight-for-age z-score. Whether the infant is below 2 standard deviations from the median WHO Child Growth Standards. |
| Primary | Health outcomes | Height-for-age | Height-for-age z-score. Whether the infant is below 2 standard deviations from the median WHO Child Growth Standards. |
| Primary | Civic participation | Participation | Index of activities associated with empowerment and efficacy. The following outcomes were combined into an unweighted mean effects index as described in Section V of the paper.  1) Whether the respondent reported that she (or a household member) participated in communal activities over the previous 12 months, in which people came together to work for the benefit of the community.  2) Whether the respondent reported that over the previous 12 months, people in her neighborhood or village had gotten together to petition government officials or political leaders for something benefiting the community.  3) Whether the respondent reported that she (or a household member) had done at least one of the following in the past 12 months –   - attended a village or neighborhood council meeting, public hearing, discussion group - met with a politician, called him/her, or sent a letter - participated in a protest or demonstration - participated in an information or election campaign - alerted newspaper, radio or TV to a local problem - notified police or court about a local problem - online activism (such as reporting problems on Facebook, Twitter, etc.) |
| Primary | Empowerment | Perceptions of empowerment | The perception of the respondent about her power to make important decisions and take actions that improve life in her village, for herself and others. This was assessed on a 4-point scale, where 1 means being totally unable to improve life in this village, and 4 means having full control to make important decisions and actions to improve life in this village. |
| Secondary | Utilization of MNH services | Birth preparedness | Number of birth preparedness activities conducted by the respondent:   1. Where to deliver the baby 2. Who will assist with the birth 3. Transportation to place of delivery 4. Payment for delivery 5. Identification of a compatible blood donor 6. Support to look after children while away (for women with childcare responsibilities only) 7. Support to look after the home while away |
| Secondary | Health outcomes | Birth weight | Whether the infant has a birth weight less than 2500g |
| Secondary | Health outcomes | Maternal depression | Respondent’s score on the Kessler Psychological Distress Scale (K6), measured by converting the K6 to a 0-24 scale (each of the six questions coded 0-4 and summed), with 13+ indicating SMI. |

**Appendix A3. Intermediate Outcomes**

**Table A3.1. Indonesia**

|  | **Outcome** | **Definition** |
| --- | --- | --- |
| **Increased awareness, knowledge and improved community attitudes** | | |
|  | Knowledge of ANC and facility birth | Number of “disagree” responses to the following statements –   1. If a pregnant woman has already had a baby and did not experience complications, she only needs to seek antenatal care if she has problems with her current pregnancy. 2. It is fine to stay at home during labor and wait until a woman begins having complications to go to a health facility. 3. It is just as safe to give birth at home with a baby dukun (TBA) as it is to give birth in the health facility. |
|  | Knowledge of pregnancy problems | Number of relevant problems mentioned by the respondent when asked, “From your knowledge, what problems in pregnancy might need medical treatment?” |
|  | Knowledge of birth preparedness | Number of relevant actions mentioned by the respondent when asked, “From your knowledge, what actions should a woman take for birth preparedness planning?” |
|  | Knowledge of postnatal complications | Number of relevant responses to the question, “What complications (immediately following childbirth) do you know of?” |
|  | Knowledge of T4D levers | Based on responses to the following questions on T4D health levers –   1. From your knowledge, when is it recommended for pregnant mothers to first seek antenatal care? (Appropriate answer: Within 13 weeks/3 months/1^st^ trimester) 2. From your knowledge, what is the total number of ANC visits a pregnant woman is supposed to receive during her pregnancy? (Appropriate answer: 4 or more) 3. After birth, does a baby need to be checked by health staff for postnatal care? (Appropriate answer: Yes) 4. Within how many days or weeks of birth should a baby see a health provider for postnatal care? (Appropriate answer: 7 days/1 week) |
|  | Attitudes on partner participation | Based on responses to the following questions on partner participation –   1. My husband/partner supported me throughout my pregnancy. (Appropriate answer: Strongly Agree/Agree) 2. Issues of pregnancy, birth, and infant care are ‘women’s issues’ and it is not important for the father of the child to be involved. (Appropriate answer: Strongly Disagree/Disagree) 3. In your opinion, who should be the most important decision maker in where a pregnant woman or mother seeks care for herself or her child? (Appropriate answer: The mother) 4. Was your husband/partner with you when you delivered [name of child]? (Appropriate answer: Yes) |
| **Improved facility access (transportation, new facility, longer facility hours, outreach services)** | | |
|  | Traveled to facility by means other than on foot | Based on response to the question, “What form of transport did you take to the facility for your delivery?” |
|  | Traveled to facility by ambulance | Based on response to the question, “What form of transport did you take to the facility for your delivery?” |
|  | Travel time (hours) | Based on response to the question, “How long did it take you to travel to the facility for your delivery using the transport mentioned above?” |
|  | Travel cost (IDR) | Based on response to the question, “In total, how much did you pay for transport to the clinic to give birth to [name of child]?” |
|  | Mobile clinic serves sample village | Whether a mobile clinic or other outreach service serves the sample village. |
|  | Mobile clinic serves sample village & setup within past 3 years | Whether a mobile clinic or other outreach service serves the sample village AND was setup within the past 3 years |
|  | Number of facilities built in the last 3 years | The number of facilities built in the puskesmas catchment area in the past 3 years |
|  | New posyandus have been setup in the last 3 years | Whether any posyandus have been established or reactivated in the sample village in the past 3 years |
|  | Number of posyandu located in sample village | Number of posyandu located in sample village |
|  | Facility has functional ambulance | Whether the puskesmas has a functional ambulance |
|  | Fuel for ambulance available today | Whether fuel for the ambulance was available on the day of the survey |
| **Improved information transparency (cost, opening hours, etc.) or complaint mechanisms** | | |
|  | Information on cost of delivery services displayed | Whether information on cost of delivery services is displayed publicly at the facility |
|  | Information on cost of other services displayed | Whether information on cost of other health services is displayed publicly at the facility |
|  | Information on facility hours displayed | Whether information on the facility’s operational hours are posted publicly at the facility |
|  | Presence of complaint management system | Whether the facility has a complaint management system (e.g. suggestion box, phone number, etc.) |
|  | Number of meetings with community members in the past year | Based on responses to the questions, “Does this health facility conduct routine meeting with community members in the service area to improve the service quality of this facility?” and “How often have these community meetings happened in the past one year?” |
| **Increased availability of drugs, supplies and other inputs** | | |
|  | Number of essential medicines for mothers in stock | Whether the following medicines were in stock at the time of the survey:   1. Oxytocin 2. Sodium Chloride (saline) or Ringers Lactate 3. Calcium gluconate injection 4. Magnesium sulfate 5. Ampicillin 6. Gentamicin 7. Metronidazole 8. Misoprostol 9. Azithromycin 10. Cefixime 11. Benzathine benzylpenicillin 12. Betamathasone or Dexamethasone 13. Nifedipine 14. Iron tablets 15. Folic acid tablets 16. Diazepam |
|  | Number of essential medicines for children in stock | Whether the following medicines were in stock at the time of the survey:   1. Amoxicillin 2. Ceftriaxone 3. Oxygen 4. Procaine benzylpenicillin 5. Oral Rehydration salts (ORS) 6. Zinc 7. Artemisinin combination therapy 8. Artesunate 9. Standard regimen for first-line anti-retroviral treatment (ARV) 10. Vitamin A 11. Morphine 12. Paracetamol 13. Antibiotic eye ointment for newborns 14. Albendazole/Melbendazole tablet |
|  | Number of essential vaccines in stock | Whether the following vaccines were in stock at the time of the survey:   1. Polio 2. BCG 3. HepB vaccine 4. DPT-Hib-HepB 5. Measles/Rubella 6. Tetanus Toxoid |
|  | Number of essential equipment available & functional | Whether the following equipment were available and reported as functional at the time of the survey:   1. Blood pressure equipment 2. Infant weight scale 3. Adult weight scale 4. Measuring tape (for head, arm, hip measurement, etc.) 5. Equipment to measure infant’s height/ length 6. Adult height scale 7. Growth chart 8. Examination light 9. Suction apparatus (mucus extractor) 10. Manual/electric vacuum extractor 11. Vacuum Aspirator or D&C kit 12. Partograph 13. Cold box/vaccine carrier with ice packs 14. Refrigerator 15. Sharps container |
|  | Number of essential supplies in stock | Whether the following supplies were in stock at the time of the survey:   1. Syringes (Disposable) 2. Sterile gloves 3. Skin disinfectant 4. Thermometer 5. Stethoscope 6. Light source 7. Delivery pack 8. Neonatal bag and mask |
| **Increased ability to pay** | | |
|  | Cost reported as a barrier to healthcare | Whether respondent mentioned healthcare costs in response to the question, “Why did you not deliver at a health facility?” |
|  | Cost of delivery | Cost of delivery as reported in response to the question, “Think about the total amount of fees (if any) during your delivery. This does not include the cost of transportation to/from the facility. Only include the cost of the visit for delivery, not any prenatal or postnatal visits. How much did you pay in total?” |
|  | Paid for delivery (binary) | Whether respondent reports paying for delivery. |
|  | Insurance enrollment | Whether respondent reports using any kind of insurance/health protection program for the healthcare costs associated with delivery. |
|  | Savings group membership for MNH care | Whether respondent reports being part of a woman’s savings group to help save for costs associated with pregnancy, delivery, or postnatal care. |
|  | Used community fund for MNH care | Whether respondent reports using a village community fund to help pay for costs associated with pregnancy, delivery, or postnatal care. |
| **Improved facility infrastructure** | | |
|  | Uninterrupted electricity during opening hours in last 7 days | Based on response to the question, “During the past 7 days, was electricity (excluding any back-up generator) available during the times when the facility was open for services, or was it ever interrupted for more than 2 hours at a time?” |
|  | Facility has other sources of electricity besides central grid | Whether the facility has sources of electricity besides the central grid (e.g. solar power, generator, etc.) |
|  | Facility generator is functional | Whether the facility generator was functional |
|  | Fuel available for generator | Availability of fuel for the generator on the day of the survey |
|  | Facility solar power system is functional | Whether the facility’s solar power system is functional. |
|  | Facility has functional telephone | Whether the facility has a functional telephone available to make phone calls at all times client services are offered |
|  | Facility has functional radio | Whether the facility has a functional short-wave radio |
|  | Facility has functional email | Whether the facility has access to email or internet via computer and/or mobile phone within the facility |
|  | Water outlet available onsite | Whether a water outlet is available on the facility premises |
|  | Routine water shortage | Whether there is routinely a time of year when the facility has a severe shortage or lack of water |
|  | Duration of water shortage (in days) | Duration of the routine water shortage |
|  | Facility has specific room for deliveries | Whether there is a specific room for deliveries at the facility. |
|  | Privacy level of delivery room (1-4 scale) | Privacy level of the delivery room, on a 1-4 scale (where 1=Private Room, 2=Semi private room with visual privacy but no auditory privacy, 3=Multiple beds in the room with some partition, 4=Multiple beds in a room with no partition) |
|  | Number of designated delivery beds | Total number of designated delivery beds available at the facility |
|  | Delivery patient toilet - Flushed to septic tank/through piped system | Whether the delivery patient toilet was a squatting latrine flushed through piped sewer system or septic tank, or a sitting closet/water closet flushed through piped sewer system or septic tank. |
|  | Delivery patient toilet - Water is available to flush | Whether water was available to flush in the delivery patient toilet on the day of the survey |
|  | Delivery patient toilet - Water to wash hands | Whether water was available to wash hands in the delivery patient toilet on the day of the survey |
|  | Delivery patient toilet - Soap to wash hands | Whether soap was available to wash hands in the delivery patient toilet on the day of the survey |
|  | Delivery patient toilet - Privacy (1-3 scale) | Privacy level of the delivery patient toilet, where 1=Has door that can be locked, 2=Has door that cannot be locked, 3=Has no door |
|  | Toilet inside/next to delivery room | Whether the delivery patient toilet was inside or next to the delivery room |
|  | Biological/medical waste disposal available | Whether biological/medical waste disposal was available |
| **Increased or improved facility staffing** | | |
|  | Total number of MNH staff & resident midwives | Total number of staff and residency program midwives in the MNH and birth unit |
|  | Number of vacancies for medical staff | Number of official staff vacancies for medical staff (midwives/nurses/doctors) in the MNH and birth unit at the time of the survey |
|  | Number of vacancies for non-medical staff | Number of official staff vacancies for other staff in the MNH and birth unit at the time of the survey |
|  | Skilled delivery staff present/on call 24*7 | Whether a person skilled in conducting deliveries is present at the facility or on call at all times (24 hours a day), including on weekends, to provide delivery care |
| **Bylaws, partnerships, or other interventions aimed at health system uptake** | | |
|  | Village has by-law on MNH services | Whether the village in which this facility is located, or any of the catchment area villages, have a by-law on maternal health services (cost, delivery facility, visit to health facility, etc.) |
|  | District Govt./Sub-district Govt./Puskesmas has by-law on MNH services | Whether the district, sub district or puskesmas have a by-law on maternal health services (cost, delivery facility, visit to health facility, etc.). |
|  | Province/National Govt. has by-law on MNH services | Whether the provincial or national governments have a by-law or regulation on maternal health (cost, delivery facility, visit to health facility, etc.) |
|  | Puskesmas has partnership with Baby Dukun | Whether the facility has a partnership with traditional birth attendants (Baby Dukun) to ensure they encourage women to deliver with a midwife |
| **Improved facility cleanliness**  (Recent visit: Pertains to the respondent’s most recent visit to any health facility within the past 12 months)  (Sample Puskesmas: Pertains to the respondent’s most recent to the sample puskesmas within the past 12 months) | | |
|  | Recent visit: Cleanliness (1-4 scale) | Based on response to the question, “How would you rate the cleanliness of the facility during this visit?” on a 1 to 4 scale (where 1=Excellent, 2=Good, 3=Fair, 4=Poor). |
|  | Sample Puskesmas: Cleanliness (1-4 scale) | Based on response to the question, “How would you rate the cleanliness of the facility during this visit?” on a 1 to 4 scale (where 1=Excellent, 2=Good, 3=Fair, 4=Poor). |
|  | Delivery bed is clean | Whether delivery bed was clean at the time of the survey (i.e. no blood, fluids, or dirt visible) |
|  | Delivery room floor is clean | Whether delivery room floor was clean at the time of the survey (i.e. no blood, fluids, or dirt visible) |
|  | Delivery room is well ventilated | Whether the delivery room was well-ventilated (windows open, good air circulation) or air-conditioned |
|  | No dust or mold observed in the room | Whether dust or mold was observed in the delivery room at the time of the survey |
|  | Delivery patient toilet - Cleanliness (1-3 scale) | Cleanliness of the delivery patient toilet measured on a 1-3 scale, where 1=Toilet is clean and tidy, 2=Toilet is somewhat clean, and 3=Toilet is dirty |
| **Improved (perceived) attitude, effort, trust of the provider**  (Recent visit: Pertains to the household respondent’s most recent visit to any health facility within the past 12 months)  (Sample puskesmas: Pertains to the household respondent’s most recent to the sample puskesmas within the past 12 months) | | |
|  | Recent visit: Satisfaction with waiting time (1-4 scale) | Based on response to the question, “In general, how satisfied are you with the amount of time you have to wait to see the nurse, midwife, or other health provider when you visit the facility?” on a 1 to 4 scale (where 1=Very satisfied, 2=Satisfied, 3=Unsatisfied, 4=Very unsatisfied). |
|  | Recent visit: Longest waiting time (hours) | Longest time the respondent reported having to wait for a health worker during recent visits. |
|  | Recent visit: Satisfaction with opening hours (1-4 scale) | Based on response to the question, “How satisfied are you with the hours which this facility is open?” on a 1 to 4 scale (where 1=Very satisfied, 2=Satisfied, 3=Unsatisfied, 4=Very unsatisfied). |
|  | Recent visit: Health worker not there (1-4 scale) | Based on response to the question, “Have you ever gone to the facility and found that the health worker was not there?” on a 1 to 4 scale (where 1=Yes, often, 2=Yes, sometimes, 3=Yes, once, 4=No). |
|  | Recent visit: Respectful treatment (1-4 scale) | Based on response to the question, “How would you rate the respect the providers show you at this facility?” on a 1 to 4 scale (where 1=Excellent, 2=Good, 3=Fair, 4=Poor). |
|  | Recent visit: Trust nurses/midwives/other staff (1-4 scale) | Based on response to the question, “Overall, to what degree do you trust nurses, midwives, or other staff at this facility?” on a 1 to 4 scale (where 1=Excellent, 2=Good, 3=Fair, 4=Poor). |
|  | Recent visit: Availability of drugs/supplies/equipment (1-4 scale) | Based on response to the question, “How would you rate the availability of drugs, supplies and medical equipment at this facility?” on a 1 to 4 scale (where 1=Excellent, 2=Good, 3=Fair, 4=Poor). |
|  | Recent visit: Quality of physical facility (1-4 scale) | Based on response to the question, “How would you rate the quality of the physical facility, including the building, furnishing, lights, water, etc.?” on a 1 to 4 scale (where 1=Excellent, 2=Good, 3=Fair, 4=Poor). |
|  | Recent visit: Overall quality of healthcare (1-4 scale) | Based on response to the question, “Overall, taking everything into account, how would you rate the quality of care you receive at this facility?” on a 1 to 4 scale (where 1=Excellent, 2=Good, 3=Fair, 4=Poor). |
|  | Recent visit: If people complained, would staff improve | Based on response to the question, “If members of this community had a complaint about [name of facility], and brought it to the attention of the health staff at [name of facility] facility, do you think s/he would try to make an improvement?” |
|  | Recent visit: Change in the last year (Better care/Worse/Same) | Based on response to the question, “In the last 12 months, do you think that the quality of care at this facility has gotten better, worse, or stayed about the same?” |
|  | Sample Puskesmas: Satisfaction with waiting time (1-4 scale) | Based on response to the question, “In general, how satisfied are you with the amount of time you have to wait to see the nurse, midwife, or other health provider when you visit the facility?” on a 1 to 4 scale (where 1=Very satisfied, 2=Satisfied, 3=Unsatisfied, 4=Very unsatisfied). |
|  | Sample Puskesmas: Longest waiting time (hours) | Longest time the respondent reported having to wait for a health worker during recent visits. |
|  | Sample Puskesmas: Satisfaction with opening hours (1-4 scale) | Based on response to the question, “How satisfied are you with the hours which this facility is open?” on a 1 to 4 scale (where 1=Very satisfied, 2=Satisfied, 3=Unsatisfied, 4=Very unsatisfied). |
|  | Sample Puskesmas: Health worker not there (1-4 scale) | Based on response to the question, “Have you ever gone to the facility and found that the health worker was not there?” on a 1 to 4 scale (where 1=Yes, often, 2=Yes, sometimes, 3=Yes, once, 4=No). |
|  | Sample Puskesmas: Respectful treatment (1-4 scale) | Based on response to the question, “How would you rate the respect the providers show you at this facility?” on a 1 to 4 scale (where 1=Excellent, 2=Good, 3=Fair, 4=Poor). |
|  | Sample Puskesmas: Trust nurses/midwives/other staff (1-4 scale) | Based on response to the question, “Overall, to what degree do you trust nurses, midwives, or other staff at this facility?” on a 1 to 4 scale (where 1=Excellent, 2=Good, 3=Fair, 4=Poor). |
|  | Sample Puskesmas: Availability of drugs/supplies/equipment (1-4 scale) | Based on response to the question, “How would you rate the availability of drugs, supplies and medical equipment at this facility?” on a 1 to 4 scale (where 1=Excellent, 2=Good, 3=Fair, 4=Poor). |
|  | Sample Puskesmas: Quality of physical facility (1-4 scale) | Based on response to the question, “How would you rate the quality of the physical facility, including the building, furnishing, lights, water, etc.?” on a 1 to 4 scale (where 1=Excellent, 2=Good, 3=Fair, 4=Poor). |
|  | Sample Puskesmas: Overall quality of healthcare (1-4 scale) | Based on response to the question, “Overall, taking everything into account, how would you rate the quality of care you receive at this facility?” on a 1 to 4 scale (where 1=Excellent, 2=Good, 3=Fair, 4=Poor). |
|  | Sample Puskesmas: If people complained, would staff improve | Based on response to the question, “If members of this community had a complaint about [name of facility] facility, and brought it to the attention of the health staff at [name of facility] facility, do you think s/he would try to make an improvement?” |
|  | Sample Puskesmas: Change in the last year (Better care/Worse/Same) | Based on response to the question, “In the last 12 months, do you think that the quality of care at this facility has gotten better, worse, or stayed about the same?” |
|  | Non-dignified care | Whether the respondent reported experiencing non-dignified care (health providers shouting at/scolding the patient, or withholding/threatening to withhold treatment because patient could not pay or did not have supplies) during her recent delivery at the facility. |
|  | Provider neglect | Whether the respondent reported experiencing neglect (health providers ignoring/abandoning the patient, or patient delivering without assistance from health providers) during her recent delivery at the facility. |
|  | Provider abuse | Whether the respondent reported experiencing abuse (health provider hitting/slapping/pushing/pinching/beating patient) during her recent delivery at the facility. |
|  | Healthcare provider perception: Women are well aware of the MNH services offered at [facility name] | Based on provider response to the statement, “Women are generally well aware of the maternal/new-born health services offered at [puskesmas name]” on 1-4 scale, where 1=Strongly agree, 2=Somewhat agree, 3=Somewhat disagree, 4=Strongly disagree |
|  | Healthcare provider perception: Women seeking MNH services understand the problems in running [facility name] | Based on provider response to the statement, “Women seeking maternal/new-born health services understand the constraints and problems that arise in running [puskesmas name]” on 1-4 scale, where 1=Strongly agree, 2=Somewhat agree, 3=Somewhat disagree, 4=Strongly disagree |
|  | Healthcare provider perception: When [facility name] is making a decision about MNH, community members are consulted | Based on provider response to the statement, “When [puskesmas name] is making a decision about something that affects the maternal/new-born health services the community will receive, community members are consulted about it” on 1-4 scale, where 1=Strongly agree, 2=Somewhat agree, 3=Somewhat disagree, 4=Strongly disagree |
|  | Healthcare provider perception: In general, maternal health patients do not have meaningful feedback | Based on provider response to the statement, “In general, maternal health patients do not have meaningful feedback on your services” on 1-4 scale, where 1=Strongly agree, 2=Somewhat agree, 3=Somewhat disagree, 4=Strongly disagree |
|  | Healthcare provider perception: Most patients don’t appreciate the effort put in by the staff | Based on provider response to the statement, “Most patients don’t appreciate the level of effort put in by the staff at [puskesmas name]” on 1-4 scale, where 1=Strongly agree, 2=Somewhat agree, 3=Somewhat disagree, 4=Strongly disagree |
|  | Healthcare provider perception: Patients communicate about their feedback on MNH services | Based on provider response to the statement, “Patients generally communicate with you about their feedback on maternal/new-born health services” on 1-4 scale, where 1=Strongly agree, 2=Somewhat agree, 3=Somewhat disagree, 4=Strongly disagree |
|  | Midwife assigned to sample village | Whether a village midwife has been assigned to the sample village |
|  | Assigned midwife lives in sample village | Whether the assigned midwife lives in the sample village |
|  | Midwife receives free housing in village | Whether the assigned midwife received free housing in the village |
| **Improved provider knowledge** | | |
|  | Provider responded correctly to ANC question | Whether the provider strongly disagrees with the statement, “If a pregnant mother has already had a baby and did not experience complications before, then the mother does not need to seek antenatal care for her current pregnancy.” |
|  | Provider responded correctly to delivery question | Whether the provider strongly disagrees with the statement, “It is fine to stay at home during labor and wait until a woman begins having complications to go to a health facility” |
|  | Number of reasons to refer woman to hospital for birth | Number of relevant reasons mentioned by the provider when asked, “For what reasons would you refer a woman to give birth in a puskesmas or hospital (rather than at a pustu/ polindes/ poskesdes/ other village-based health facility or midwife private practice)?” |

**Table A3.2. Tanzania**

|  | **Outcome** | **Definition** |
| --- | --- | --- |
| **Increased awareness, knowledge and improved community attitudes** | | |
|  | Knowledge of ANC and facility birth | Number of “disagree” responses to the following statements –   1. If a pregnant woman has already had a baby and did not experience complications, she only needs to seek antenatal care if she has problems with her current pregnancy. 2. It is fine to stay at home during labor and wait until a woman begins having complications to go to a health facility. 3. It is just as safe to give birth at home with a baby dukun (TBA) as it is to give birth in the health facility. |
|  | Knowledge of pregnancy problems | Number of relevant problems mentioned by the respondent when asked, “From your knowledge, what problems in pregnancy might need medical treatment?” |
|  | Knowledge of birth preparedness | Number of relevant actions mentioned by the respondent when asked, “From your knowledge, what actions should a woman take for birth preparedness planning?” |
|  | Knowledge of postnatal complications | Number of relevant responses to the question, “What complications (immediately following childbirth) do you know of?” |
|  | Knowledge of T4D levers | Based on responses to the following questions on T4D health levers –   1. From your knowledge, when is it recommended for pregnant mothers to first seek antenatal care? (Appropriate answer: Within 13 weeks/3 months/1^st^ trimester) 2. From your knowledge, what is the total number of ANC visits a pregnant woman is supposed to receive during her pregnancy? (Appropriate answer: 4 or more) 3. After birth, does a baby need to be checked by health staff for postnatal care? (Appropriate answer: Yes) 4. Within how many days or weeks of birth should a baby see a health provider for postnatal care? (Appropriate answer: 7 days/1 week) |
|  | Attitudes on partner participation | Based on responses to the following questions on partner participation –   1. My husband/partner supported me throughout my pregnancy. (Appropriate answer: Strongly Agree/Agree) 2. Issues of pregnancy, birth, and infant care are ‘women’s issues’ and it is not important for the father of the child to be involved. (Appropriate answer: Strongly Disagree/Disagree) 3. In your opinion, who should be the most important decision maker in where a pregnant woman or mother seeks care for herself or her child? (Appropriate answer: The mother) 4. Was your husband/partner with you when you delivered [name of child]? (Appropriate answer: Yes) |
| **Improved facility access** | | |
|  | Traveled to facility by means other than on foot | Based on response to the question, “What form of transport did you take to the facility for your delivery?” |
|  | Travel time (hours) | Based on response to the question, “How long did it take you to travel to the facility for your delivery using the transport mentioned above?” |
|  | Travel cost (TZS) | Based on response to the question, “In total, how much did you pay for transport to the clinic to give birth to [name of child]?” |
|  | Facility has functional ambulance | Whether the facility has a functional ambulance or other vehicle for emergency transportation for clients stationed at the facility. |
|  | Facility has access to vehicle for emergencies | Whether the facility has access to an ambulance, other vehicle for emergency transportation for clients stationed at another facility, or that operates from another facility. |
|  | Village served by mobile clinic | Whether a mobile clinic or other outreach service serves the sample village. |
|  | Village served by mobile clinic setup within past 3 years | Whether a mobile clinic or other outreach service serves the sample village AND was setup within the past 3 years |
|  | Village has health dispensary | Whether a dispensary is located in the sample village |
| **Improved information transparency (cost, opening hours, etc.) or complaint mechanisms** | | |
|  | Facility: Information on cost of delivery services displayed | Whether information on cost of delivery services is displayed publicly at the facility |
|  | Facility: Information on cost of other services displayed | Whether information on cost of other health services is displayed publicly at the facility |
|  | Facility: Information on facility hours displayed | Whether information on the facility’s operational hours are posted publicly at the facility |
|  | Facility: Presence of complaint management system | Whether the facility has a complaint management system (e.g. suggestion box, phone number, etc.) |
|  | Facility: Number of meetings with community members in the past year | Based on responses to the questions, “Does this health facility conduct routine meeting with community members in the service area to improve the service quality of this facility?” and “How often have these community meetings happened in the past one year?” |
| **Bylaws, partnerships, or other interventions aimed at health system uptake** | | |
|  | Facility: Health center/dispensary has by-law on MNH services | Whether the sample dispensary or the associated health center has a local regulation/by-law on maternal health services (cost, delivery at a facility, visit to health facility, etc.) |
|  | Facility: Dispensary has partnership with TBAs | Whether the dispensary has a partnership with TBAs to ensure they encourage women to deliver in the facility with a midwife |
|  | Facility: Presence of programs/campaigns to educate TBAs | Whether the respondent knows of any programs or campaigns aimed at educating TBAs in the area, or convincing them to not deliver babies at home |
|  | Village has by-law on MNH services | Whether the sample village has a local regulation/by-law on maternal health services (cost, delivery at a facility, visit to health facility, etc.) |
| **Increased ability to pay** | | |
|  | Cost reported as a barrier to healthcare | Whether respondent mentioned healthcare costs in response to the question, “Why did you not deliver at a health facility?” |
|  | Cost of delivery | Cost of delivery as reported in response to the question, “Think about the total amount of fees (if any) during your delivery. This does not include the cost of transportation to/from the facility. Only include the cost of the visit for delivery, not any prenatal or postnatal visits. How much did you pay in total?” |
|  | Paid for delivery (binary) | Whether respondent reported paying for delivery. |
|  | Insurance enrollment and usage | Whether respondent reported using any kind of insurance/health protection program for the healthcare costs associated with delivery. |
|  | Savings group membership for MNH care | Whether respondent reports being part of a woman’s savings group to help save for costs associated with pregnancy, delivery, or postnatal care. |
|  | Used community fund for MNH care | Whether respondent reports using a village community fund to help pay for costs associated with pregnancy, delivery, or postnatal care. |
| **Improved attitude, effort, trust of the provider**  (Recent visit: Pertains to the household respondent’s most recent visit to any health facility within the past 12 months)  (Sample dispensary: Pertains to the household respondent’s most recent to the sample dispensary within the past 12 months) | | |
|  | Recent visit: Satisfaction with waiting time (1-4 scale) | Based on response to the question, “In general, how satisfied are you with the amount of time you have to wait to see the nurse, midwife, or other health provider when you visit the facility?” on a 1 to 4 scale (where 4=Very satisfied, 3=Satisfied, 2=Unsatisfied, 1=Very unsatisfied). |
|  | Recent visit: Longest waiting time (hours) | Longest time the respondent reported having to wait for a health worker during recent visits. |
|  | Recent visit: Satisfaction with opening hours (1-4 scale) | Based on response to the question, “How satisfied are you with the hours which this facility is open?” on a 1 to 4 scale (where 4=Very satisfied, 3=Satisfied, 2=Unsatisfied, 1=Very unsatisfied). |
|  | Recent visit: Health worker not there (1-4 scale) | Based on response to the question, “Have you ever gone to the facility and found that the health worker was not there?” on a 1 to 4 scale (where 4=Yes, often, 3=Yes, sometimes, 2=Yes, once, 1=No). |
|  | Recent visit: Respectful treatment (1-4 scale) | Based on response to the question, “How would you rate the respect the providers show you at this facility?” on a 1 to 4 scale (where 4=Excellent, 3=Good, 2=Fair, 1=Poor). |
|  | Recent visit: Ever refused treatment (1-4 scale) | Based on response to the question, “Have you ever been turned away or refused treatment by someone at the health facility when you visited?” on a 1 to 4 scale (where 1=Yes, often, 2=Yes, sometimes, 3=Yes, once, 4=No). |
|  | Recent visit: Trust nurses/midwives/other staff (1-4 scale) | Based on response to the question, “Overall, to what degree do you trust nurses, midwives, or other staff at this facility?” on a 1 to 4 scale (where 4=Excellent, 3=Good, 2=Fair, 1=Poor). |
|  | Recent visit: Availability of drugs/supplies/equipment (1-4 scale) | Based on response to the question, “How would you rate the availability of drugs, supplies and medical equipment at this facility?” on a 1 to 4 scale (where 4=Excellent, 3=Good, 2=Fair, 1=Poor). |
|  | Recent visit: Staff communication skills (1-4 scale) | Based on response to the question, “How would you rate the communication skills of the providers at this facility? In other words, how well did they explain things to you?” on a 1 to 4 scale (where 4=Excellent, 3=Good, 2=Fair, 1=Poor). |
|  | Recent visit: Quality of physical facility (1-4 scale) | Based on response to the question, “How would you rate the quality of the physical facility, including the building, furnishing, lights, water, etc.?” on a 1 to 4 scale (where 4=Excellent, 3=Good, 2=Fair, 1=Poor). |
|  | Recent visit: Overall quality of healthcare (1-4 scale) | Based on response to the question, “Overall, taking everything into account, how would you rate the quality of care you receive at this facility?” on a 1 to 4 scale (where 1=Excellent, 2=Good, 3=Fair, 4=Poor). |
|  | Recent visit: If people complained, would staff improve | Based on response to the question, “If members of this community had a complaint about [name of facility] facility, and brought it to the attention of the health staff at [name of facility] facility, do you think s/he would try to make an improvement?” |
|  | Recent visit: Change in the last year (Better care/Same/Worse) | Based on response to the question, “In the last 12 months, do you think that the quality of care at this facility has gotten better, worse, or stayed about the same?” |
|  | Recent visit: Greeted promptly | Whether respondent reported being greeted/attended to promptly at the facility, based on responses to the question, “In general, how are you received at the facility?” |
|  | Recent visit: Insufficient seating | Whether respondent reported there being insufficient seating at the facility, based on responses to the question, “In general, how are you received at the facility?” |
|  | Recent visit: Staff understood reason for visit | Whether respondent reported that staff made sure they understood the reason for the visit, based on responses to the question, “In general, how are you received at the facility?” |
|  | Recent visit: Staff used bad language | Whether staff at the facility were using bad language, based on responses to the question, “In general, how are you received at the facility?” |
|  | Recent visit: Properly informed | Whether respondent was kept properly informed of what was happening during the visit, based on responses to the question, “In general, how are you received at the facility?” |
|  | Recent visit: Not given options for treatment | Whether respondent reported not being given appropriate options for treatment, based on responses to the question, “In general, how are you received at the facility?” |
|  | Sample Dispensary: Satisfaction with waiting time (1-4 scale) | Based on response to the question, “In general, how satisfied are you with the amount of time you have to wait to see the nurse, midwife, or other health provider when you visit the facility?” on a 1 to 4 scale (where 4=Very satisfied, 3=Satisfied, 2=Unsatisfied, 1=Very unsatisfied). |
|  | Sample Dispensary: Longest waiting time (hours) | Longest time the respondent reported having to wait for a health worker during recent visits. |
|  | Sample Dispensary: Satisfaction with opening hours (1-4 scale) | Based on response to the question, “How satisfied are you with the hours which this facility is open?” on a 1 to 4 scale (where 4=Very satisfied, 3=Satisfied, 2=Unsatisfied, 1=Very unsatisfied). |
|  | Sample Dispensary: Health worker not there (1-4 scale) | Based on response to the question, “Have you ever gone to the facility and found that the health worker was not there?” on a 1 to 4 scale (where 4=Yes, often, 3=Yes, sometimes, 2=Yes, once, 1=No). |
|  | Sample Dispensary: Respectful treatment (1-4 scale) | Based on response to the question, “How would you rate the respect the providers show you at this facility?” on a 1 to 4 scale (where 4=Excellent, 3=Good, 2=Fair, 1=Poor). |
|  | Sample Dispensary: Ever refused treatment (1-4 scale) | Based on response to the question, “Have you ever been turned away or refused treatment by someone at the health facility when you visited?” on a 1 to 4 scale (where 1=Yes, often, 2=Yes, sometimes, 3=Yes, once, 4=No). |
|  | Sample Dispensary: Trust nurses/midwives/other staff (1-4 scale) | Based on response to the question, “Overall, to what degree do you trust nurses, midwives, or other staff at this facility?” on a 1 to 4 scale (where 4=Excellent, 3=Good, 2=Fair, 1=Poor). |
|  | Sample Dispensary: Availability of drugs/supplies/equipment (1-4 scale) | Based on response to the question, “How would you rate the availability of drugs, supplies and medical equipment at this facility?” on a 1 to 4 scale (where 4=Excellent, 3=Good, 2=Fair, 1=Poor). |
|  | Sample Dispensary: Staff communication skills (1-4 scale) | Based on response to the question, “How would you rate the communication skills of the providers at this facility? In other words, how well did they explain things to you?” on a 1 to 4 scale (where 4=Excellent, 3=Good, 2=Fair, 1=Poor). |
|  | Sample Dispensary: Quality of physical facility (1-4 scale) | Based on response to the question, “How would you rate the quality of the physical facility, including the building, furnishing, lights, water, etc.?” on a 1 to 4 scale (where 4=Excellent, 3=Good, 2=Fair, 1=Poor). |
|  | Sample Dispensary: Overall quality of healthcare (1-4 scale) | Based on response to the question, “Overall, taking everything into account, how would you rate the quality of care you receive at this facility?” on a 1 to 4 scale (where 1=Excellent, 2=Good, 3=Fair, 4=Poor). |
|  | Sample Dispensary: If people complained, would staff improve | Based on response to the question, “If members of this community had a complaint about [name of facility] facility, and brought it to the attention of the health staff at [name of facility] facility, do you think s/he would try to make an improvement?” |
|  | Sample Dispensary: Change in the last year (Better care/Same/Worse) | Based on response to the question, “In the last 12 months, do you think that the quality of care at this facility has gotten better, worse, or stayed about the same?” |
|  | Sample Dispensary: Greeted promptly | Whether respondent reported being greeted/attended to promptly at the facility, based on responses to the question, “In general, how are you received at the facility?” |
|  | Sample Dispensary: Insufficient seating | Whether respondent reported there being insufficient seating at the facility, based on responses to the question, “In general, how are you received at the facility?” |
|  | Sample Dispensary: Staff understood reason for visit | Whether respondent reported that staff made sure they understood the reason for the visit, based on responses to the question, “In general, how are you received at the facility?” |
|  | Sample Dispensary: Staff used bad language | Whether staff at the facility were using bad language, based on responses to the question, “In general, how are you received at the facility?” |
|  | Sample Dispensary: Properly informed | Whether respondent was kept properly informed of what was happening during the visit, based on responses to the question, “In general, how are you received at the facility?” |
|  | Sample Dispensary: Not given options for treatment | Whether respondent reported not being given appropriate options for treatment, based on responses to the question, “In general, how are you received at the facility?” |
|  | Non-dignified care | Whether the respondent reported experiencing non-dignified care (health providers shouting at/scolding the patient, or withholding/threatening to withhold treatment because patient could not pay or did not have supplies) during her recent delivery at the facility. |
|  | Provider neglect | Whether the respondent reported experiencing neglect (health providers ignoring/abandoning the patient, or patient delivering without assistance from health providers) during her recent delivery at the facility. |
|  | Provider abuse | Whether the respondent reported experiencing abuse (health provider hitting/slapping/pushing/pinching/beating patient) during her recent delivery at the facility. |
|  | Facility (Healthcare provider perception): Women are well aware of the MNH services offered at [dispensary name] | Based on provider response to the statement, “Women are generally well aware of the maternal/new-born health services offered at [puskesmas name]” on 1-4 scale, where 1=Strongly agree, 2=Somewhat agree, 3=Somewhat disagree, 4=Strongly disagree |
|  | Facility (Healthcare provider perception): Women seeking MNH understand the problems in running [dispensary name] | Based on provider response to the statement, “Women seeking maternal/new-born health services understand the constraints and problems that arise in running [puskesmas name]” on 1-4 scale, where 1=Strongly agree, 2=Somewhat agree, 3=Somewhat disagree, 4=Strongly disagree |
|  | Facility (Healthcare provider perception): Community members consulted when [dispensary] makes a decision on MNH | Based on provider response to the statement, “When [puskesmas name] is making a decision about something that affects the maternal/new-born health services the community will receive, community members are consulted about it” on 1-4 scale, where 1=Strongly agree, 2=Somewhat agree, 3=Somewhat disagree, 4=Strongly disagree |
|  | Facility (Healthcare provider perception): In general, maternal health patients do not have meaningful feedback | Based on provider response to the statement, “In general, maternal health patients do not have meaningful feedback on your services” on 1-4 scale, where 1=Strongly agree, 2=Somewhat agree, 3=Somewhat disagree, 4=Strongly disagree |
|  | Facility (Healthcare provider perception): Most patients don’t appreciate the effort put in by the staff | Based on provider response to the statement, “Most patients don’t appreciate the level of effort put in by the staff at [puskesmas name]” on 1-4 scale, where 1=Strongly agree, 2=Somewhat agree, 3=Somewhat disagree, 4=Strongly disagree |
|  | Facility (Healthcare provider perception): Patients communicate about their feedback on MNH services | Based on provider response to the statement, “Patients generally communicate with you about their feedback on maternal/new-born health services” on 1-4 scale, where 1=Strongly agree, 2=Somewhat agree, 3=Somewhat disagree, 4=Strongly disagree |
|  | Village: Whether CHW assigned to sample village | Whether any community health workers/village health workers (CHW/VHWs) have been assigned to the sample village |
|  | Village: Number of CHWs assigned to sample village | Number of community health workers/village health workers (CHW/VHWs) assigned to the sample village |
|  | Village: Whether assigned CHWs live in village | Whether community health workers/village health workers (CHW/VHWs) assigned to the sample village live in the sample village |
| **Increased or improved facility staffing** | | |
|  | Number of paid medical staff supposed to work at facility | Total number of paid medical workers who are supposed to work at the facility |
|  | Number of paid medical staff who actually work at facility | Total number of paid medical workers who work at the facility |
|  | Number of volunteers who work at facility | Total number of volunteers who work at the facility |
|  | Whether there are any staff vacancies | Whether any positions for paid medical staff are unfilled (vacant) |
|  | Number of staff vacancies | Number of positions for paid medical staff which are unfilled (vacant) |
|  | Skilled delivery staff present/on call 24*7 | Whether a person skilled in conducting deliveries is present at the facility or on call at all times (24 hours a day), including weekends, to provide delivery care. |
|  | Facility staff can be contacted by cellphone | Whether the response to the question, “How would a pregnant mother contact health staff if the facility is closed or staff member not present at the time?” includes “mobile phone”. |
| **Improved facility infrastructure** | | |
|  | Facility is connected to the central electricity grid | Whether the facility is connected to the main electricity grid |
|  | Facility had uninterrupted electricity during opening hours in last 7 days | Based on response to the question, “During the past 7 days, was electricity (excluding any back-up generator) available during the times when the facility was open for services, or was it ever interrupted for more than 2 hours at a time?” |
|  | Facility has other sources of electricity besides central grid | Whether the facility has sources of electricity besides the central grid (e.g. solar power, generator, etc.) |
|  | Facility solar power system is functional | Whether the facility has a functional solar power system |
|  | Facility has functional telephone | Whether the facility has a functional telephone available to make phone calls at all times client services are offered |
|  | Facility has functional radio | Whether the facility has a functional short-wave radio |
|  | Facility has functional computer | Whether the facility has a functional computer |
|  | Facility has functional email | Whether the facility has access to email or internet via computer and/or mobile phone within the facility |
|  | Water outlet available onsite | Whether a water outlet is available on the facility premises |
|  | Routine water shortage | Whether there was routinely a time of year when the facility has a severe shortage or lack of water |
|  | Duration of water shortage (in days) | Duration of the routine water shortage |
|  | Facility has specific room for deliveries | Whether there was a specific room for deliveries at the facility. |
|  | Privacy level of delivery room (1-4 scale) | Privacy level of the delivery room, on a 1-4 scale (where 1=Private Room, 2=Semi private room with visual privacy but no auditory privacy, 3=Multiple beds in the room with some partition, 4=Multiple beds in a room with no partition) |
|  | Number of designated delivery beds | Total number of designated delivery beds available at the facility |
|  | Facility has maternity home or resting place for women | Whether the facility had a maternity home, or a resting place for women to wait near the health facility. |
|  | Facility has placenta pit | Whether there was a pit for placenta disposal outside of the facility |
|  | Delivery patient toilet: Flushed to septic tank/through piped system | Whether the type of toilet used by delivery patients has a flush to a piped sewer system or septic tank |
|  | Toilet inside/next to delivery room | Whether the delivery patient toilet was inside or next to the delivery room |
|  | Delivery patient toilet - Privacy (1-3 scale) | Privacy level of the delivery patient toilet, where 1=Has door that can be locked, 2=Has door that cannot be locked, 3=Has no door |
| **Improved facility cleanliness**  (Recent visit: Pertains to the household respondent’s most recent visit to any health facility within the past 12 months)  (Sample dispensary: Pertains to the household respondent’s most recent to the sample dispensary within the past 12 months) | | |
|  | Recent visit: Facility cleanliness (1-4 scale) | Based on response to the question, “How would you rate the cleanliness of the facility during this visit?” on a 1 to 4 scale (where 4=Excellent, 3=Good, 2=Fair, 1=Poor). |
|  | Sample Dispensary: Facility cleanliness (1-4 scale) | Based on response to the question, “How would you rate the cleanliness of the facility during this visit?” on a 1 to 4 scale (where 4=Excellent, 3=Good, 2=Fair, 1=Poor). |
|  | Delivery bed is clean | Whether delivery bed was clean at the time of the survey (i.e. no blood, fluids, or dirt visible) |
|  | Delivery room floor is clean | Whether delivery room floor was clean at the time of the survey (i.e. no blood, fluids, or dirt visible) |
|  | Delivery room is well ventilated | Whether the delivery room was well-ventilated (windows open, good air circulation) or air-conditioned |
|  | Biological/medical waste disposal available | Whether biological/medical waste disposal was available. |
|  | No dust or mold observed in the room | Whether dust or mold was observed in the delivery room at the time of the survey |
|  | Delivery patient toilet - Water is available to flush | Whether water was available to flush in the delivery patient toilet on the day of the survey |
|  | Delivery patient toilet - Water to wash hands | Whether water was available to wash hands in the delivery patient toilet on the day of the survey |
|  | Delivery patient toilet - Soap to wash hands | Whether soap was available to wash hands in the delivery patient toilet on the day of the survey |
|  | Delivery patient toilet - Cleanliness (1-3 scale) | Cleanliness of the delivery patient toilet measured on a 1-3 scale, where 1=Toilet is clean and tidy, 2=Toilet is somewhat clean, and 3=Toilet is dirty |
| **Increased availability of drugs, supplies and other inputs** | | |
|  | Number of essential medicines for mothers in stock | Whether the following medicines were in stock at the time of the survey:   1. Oxytocin 2. Sodium Chloride (saline) or ringers lactate 3. Calcium gluconate injection 4. Magnesium sulfate 5. Ampicillin 6. Gentamicin 7. Metronidazole 8. Misoprostol 9. Azithromycin 10. Cefixime 11. Benzathine benzylpenicillin 12. Betamathasone or Dexamethasone 13. Nifedipine 14. Iron tablets or syrup 15. Folic acid tablets 16. Diazepam |
|  | Number of essential medicines for children in stock | Whether the following medicines were in stock at the time of the survey:   1. Amoxicillin 2. Ceftriaxone 3. Oxygen 4. Procaine benzylpenicillin 5. Oral Rehydration salts (ORS) 6. Zinc 7. Artemisinin combination therapy 8. Artesunate 9. Standard regimen for first-line anti-retroviral treatment (ARV) 10. Vitamin A 11. Morphine 12. Paracetamol 13. Antibiotic eye ointment for newborns 14. Albendazole/Melbendazole tablet 15. Vitamin K |
|  | Number of essential vaccines in stock | Whether the following vaccines were in stock at the time of the survey:   1. Polio (OPV) 2. BCG 3. HepB vaccine 4. DPT-Hib-HepB 5. Measles/Rubella 6. Tetanus Toxoid 7. Rotavirus 8. Pneumococcal |
|  | Number of essential equipment available & functional | Whether the following equipment were available and reported as functional at the time of the survey:   1. Blood pressure equipment 2. Infant weight scale 3. Adult weight scale 4. Measuring tape (for head, arm, hip measurement, etc.) 5. Equipment to measure infant’s height/ length 6. Adult height scale 7. Growth chart 8. Examination light 9. Suction apparatus (mucus extractor) 10. Manual/electric vacuum extractor 11. Vacuum Aspirator or D&C kit 12. Partograph 13. Cold box/vaccine carrier with ice packs 14. Refrigerator 15. Sharps container |
|  | Number of essential supplies in stock | Whether the following supplies were in stock at the time of the survey:   1. Syringes (Disposable) 2. Sterile gloves 3. Skin disinfectant 4. Thermometer 5. Stethoscope 6. Light source 7. Delivery pack 8. Neonatal bag and mask 9. Pregnancy test |
| **Improved provider knowledge** | | |
|  | Provider responded correctly to question on whether to seek ANC | Whether the provider strongly disagrees with the statement, “If a pregnant mother has already had a baby and did not experience complications before, then the mother does not need to seek antenatal care for her current pregnancy.” |
|  | Provider responded correctly to question on ANC timing | Whether the provider responded correctly to the question, “At what point during the pregnancy do you recommend pregnant mothers first seek antenatal care?” where the correct response is within the first 3 months/12 weeks/first trimester |
|  | Provider responded correctly to delivery question | Whether the provider strongly disagrees with the statement, “It is fine to stay at home during labor and wait until a woman begins having complications to go to a health facility” |
|  | Number of reasons given to refer woman to hospital for birth | Number of relevant reasons mentioned by the provider when asked, “For what reasons would you refer a woman to give birth in a puskesmas or hospital (rather than at a pustu/ polindes/ poskesdes/ other village-based health facility or midwife private practice)?” |
|  | Number of vaccines named that an infant should be given | Number of relevant vaccines named by the provider when asked, “Which vaccinations should an infant under 1 year be given?” |

**Appendix A4. Sampling and Qualitative Data**

**Household sampling protocol for endline survey**

**Indonesia^[[24]](#footnote-24)^**

- Household survey respondents in any village were women who were residents of the village (i.e. had been living in the village for at least 6 months), and had given birth in the 12 months prior to the survey. They were randomly selected.
- To list all women who had given birth in the village in the past 12 months, the team interviewed key informants, asking for help with identifying such women. Key informants included:
  - Village midwife
  - Other midwives
  - Baby *dukun*
  - Village officer
  - Posyandu cadre
  - Any other village-specific informants who might have information about potential respondents
- The team first queried the village midwife and prepared a list of potential respondents to the best of the midwife’s knowledge. This list was then uploaded onto SurveyMETER’s data server.
- The team then went on to interview other key informants, preparing similar lists and uploading the information onto the server.
- Once all the key informants had been interviewed, the team manually checked the data to identify and reconcile duplicates (if any). A programmer/data manager then checked the data before randomly sampling respondents.
- The number of respondents sampled varied from any village varied between 18 and 60, depending on the size of the village and the number of women who had given birth in the village in the past 12 months.
- An additional number of households were selected as “replacement households”, i.e. households that would be approached in case any of the sampled households were unavailable for the survey.

**Tanzania^[[25]](#footnote-25)^**

- Key informants typically included the following:
  - - Health facility (dispensary) staff
    - Traditional Birth Attendants (TBAs)
    - Community health workers (CHWs) who lived in the village
    - *Kitongoji* (sub-village) chairpersons. In cases where a *kitongoji* chairperson couldn’t be located or was not available, the team contacted the chairperson’s spouse/sibling; ten-cell leaders (sub-villages in Tanzania were divided into groups of 10 households, called ten-cells, which had their own leader); *Boda-boda* drivers: Motorbike drivers, who served as a means of public transportation within villages; and T4D Community Representatives (CRs)
- The team contacted the Village Executive Officer (VEO) of each village in the sample and asked them for the contact information of the key informants in that village, and if there were any other key informants who should be consulted in the sampling process.
- The team began the listing process with the health facility staff. With the help of the health facility staff, the team mapped out the *kitongojis* in the village. The team then asked for the *Babies Register* (record of all women who gave birth, AND/OR sought antenatal/postnatal care at the facility) and *Birth Register* (record of women who gave birth at the facility). These two registers were used to prepare a preliminary list of women who gave birth in the village in the past 12 months.
- The team then met with all *kitongoji* chairpersons in the village, and with the help of the chairperson, listed all the women who had given birth in the past 12 months, including women who had a stillbirth or a baby that died after delivery).
- The team then showed the list compiled from the health facility records to the *kitongoji* chairperson, and matched the names from the two lists. Any names that were not in the facility list but were there in the *kitongoji* chairperson’s list were noted down. Duplicates (names repeated twice in the exact same way, or the same person listed twice under different names) were reconciled.
  - The team repeated the listing with other key informants (including TBAs, CHWs, etc.) in each *kitongoji* as identified with the help of the VEO.^[[26]](#footnote-26)^ Then, a final list was compiled and organized by *kitongoji*.
  - Once the listing was completed for all key informants, the team assigned a number to each household listed. The team then used a SurveyCTO form to draw a random set of numbers (generally 25, 30, or 35 numbers, depending on the size of the village and the number of women who had given birth in the village in the past 12 months). The households in the list corresponding to these numbers were included in the sample to be surveyed. An additional set of numbers were randomly drawn for “replacement households”, i.e. households that would be approached in case any of the sampled households were unavailable for the survey.
  - With the help of the key informants, the team located the sampled households and made an appointment with the respondent (i.e. the woman who gave birth in the previous 12 months).
  - Once the team had visited all households and scheduled appointments where respondents were available, they exchanged information on how many households they had been able to find. They identified if any of the sampled households was found ineligible for the survey (i.e. the woman on the list had given birth more than 12 months ago, or was not a resident of the village, or was unavailable for the survey for any reason). For each household found to be ineligible, a replacement household was contacted for the survey.

**Qualitative Data**

We rely in the paper on four sources of qualitative data in smaller subset groups of the 200 hundred communities where the program was offered^^[[27]](#footnote-27)^^:

1. In 81 communities of the 200 where the program was offered—41 in Indonesia and 40 in Tanzania—trained observers attended three of the meetings and answered a series of questions about engagement, discussion, and decision-making in those meetings: the first and second meetings, at which participants discussed the information provided and deliberated on activities to pursue, and the third and final follow-up meeting, at which participants discussed their progress over the previous three months and made plans to sustain their activities after the facilitator had left. In the paper, these meeting observations provide one perspective on participation in meeting discussions as well as suggestive evidence from those discussions of whether participants engaged outside the meetings in any of the activities they had planned.
2. In 65 of these communities (41 in Indonesia and 24 in Tanzania), interviewers asked follow-up questions of several participants as well as of those with whom they engaged as part of their activities: providers, officials, their neighbors, and others. In the paper, these interviews provide an additional perspective on whether the activities participants described in meetings occurred. Interviews in Indonesia were conducted after the program ended in all 41 communities where observers attended meetings; in Tanzania, interviews were conducted in 24 of the 40 communities where observers attended meetings after the program as well as two months later.
3. In 35 of these communities—16 in Indonesia and 19 in Tanzania—a second observer noted the frequency with which meeting participants made distinct contributions during the discussions or discussed activities they would attempt or had engaged in outside the meetings.^^[[28]](#footnote-28)^^
4. Four young ethnographic scholars—PhD students and junior scholars of anthropology, area studies, medical anthropology, and political science—lived for between six months and one year in eight of the 200 communities while they were offered this program before, during, and after it and attempted inductively, self-reflexively, and comparatively to understand the perspectives of participants as they experienced it, including efforts they undertook and any effects on community health care.

Communities where meetings were observed and participants interviewed were selected randomly from the same national and regional stratifications as those offered the program as part of the broader RCT. The eight villages where ethnographic studies were undertaken were selected purposefully from within these stratifications to be geographically close to each other to allow each ethnographer to cover three villages.

Finally, two years after the program, a trained interviewer invited participants in all 200 communities to a focus group in which they were asked to reflect on the program and any activities they remembered, including challenges they had faced and any changes they had seen as a result. They also asked whether participants thought that their efforts had improved their health care overall, whether they were still meeting and trying to improve their health care or their communities in other ways, any personal benefits or costs from participating, and overall whether or not they were glad that they had participated.

**Appendix A5. Impact on Primary and Secondary outcomes controlling for village-level baseline value of the outcome**

**Table A5.1. Indonesia – Primary Outcomes**

|  | (1) | (2) | (3) | (4) | (5) | (6) | (7) | (8) |
| --- | --- | --- | --- | --- | --- | --- | --- | --- |
|  | Birth with a skilled provider | Birth at a facility | Postnatal care | Content of care | Stunting | Underweight | Empowerment - Participation | Empowerment - Vignette |
|  |  |  |  |  |  |  |  |  |
| Treatment | -0.00213 | 0.0201 | -0.0282 | 0.0775* | 0.0182 | 0.00366 | 0.0142 | 0.00584 |
|  | (0.0171) | (0.0252) | (0.0286) | (0.0422) | (0.0117) | (0.00915) | (0.0331) | (0.0290) |
| Baseline village-level average | 0.330*** | 0.363*** | 0.341*** | 0.258*** | 0.0564 | 0.0262 | -0.00573 | 0.118** |
|  | (0.0557) | (0.0853) | (0.0787) | (0.0429) | (0.0533) | (0.0416) | (0.0847) | (0.0457) |
| Constant | 0.646*** | 0.582*** | 0.205** | 0.0570 | 0.197*** | 0.151*** | 0.0402 | 1.302*** |
|  | (0.0542) | (0.0738) | (0.0814) | (0.0422) | (0.0163) | (0.0112) | (0.0316) | (0.124) |
|  |  |  |  |  |  |  |  |  |
| Observations | 6,001 | 6,001 | 5,999 | 6,001 | 5,841 | 5,855 | 6,001 | 6,001 |
| R-squared | 0.095 | 0.134 | 0.028 | 0.064 | 0.017 | 0.004 | 0.005 | 0.013 |

Notes: There were differences in how some outcomes were measured at baseline and endline. Postnatal care (Column 3) at baseline included checks conducted within 7 days of giving birth, irrespective of whether that was before or after leaving the birth facility, whereas at endline included checks conducted *after* leaving the birth facility and within 7 days of giving birth. The baseline content of care index (Column 4) does not include delivery content of care, and the list of components for postpartum and postnatal content of care differs. The list of components used to construct the Empowerment – Participation index (Column 7) also differs between baseline and endline. While the endline measure for Empowerment – vignette (Column 8) is based on a vignette, the baseline measure is based on the response to a question about whether the respondent feels that she has the power to make important decisions that can change the course of her life. For details on the differences, please refer to the questionnaires. Robust standard errors, clustered at the facility-level, in parentheses. *** p<0.01, ** p<0.05, * p<0.1

**Table A5.2. Indonesia – Secondary Outcomes**

|  | (1) | (2) | (3) | (4) | (5) |
| --- | --- | --- | --- | --- | --- |
|  | First ANC visit within the first trimester | Four or more ANC visits | Low birthweight | Birth preparedness | Content of Antenatal Care |
|  |  |  |  |  |  |
| Treatment | -0.00969 | 0.00833 | 0.00450 | 0.0110 | -0.000365 |
|  | (0.0166) | (0.0157) | (0.00765) | (0.0724) | (0.0648) |
| Baseline village-level average | 0.312*** | 0.324*** | 0.0943** | 0.0788 | 0.362*** |
|  | (0.0660) | (0.0756) | (0.0460) | (0.0629) | (0.0800) |
| Constant | 0.527*** | 0.575*** | 0.0832*** | 4.931*** | 4.840*** |
|  | (0.0499) | (0.0750) | (0.00841) | (0.231) | (0.245) |
|  |  |  |  |  |  |
| Observations | 5,911 | 5,994 | 5,423 | 6,001 | 6,001 |
| R-squared | 0.019 | 0.025 | 0.004 | 0.015 | 0.027 |

Notes: There were differences in the components of antenatal content of care measured at baseline and endline. For details on the differences, please refer to the questionnaires. Robust standard errors, clustered at the facility-level, in parentheses. *** p<0.01, ** p<0.05, * p<0.1

**Table A5.3. Tanzania – Primary Outcomes**

|  | (1) | (2) | (3) | (4) | (5) | (6) |
| --- | --- | --- | --- | --- | --- | --- |
|  | ANC visit within first trimester | Four or more ANC visits | Birth with a skilled provider | Birth at a facility | Empowerment - Participation | Empowerment - Vignette |
|  |  |  |  |  |  |  |
| Treatment | 0.0245 | 0.00183 | 0.00847 | 0.00770 | 0.0199 | -0.00731 |
|  | (0.0166) | (0.0224) | (0.0243) | (0.0253) | (0.0351) | (0.0462) |
| Baseline village-level average | 0.412*** | 0.277*** | 0.476*** | 0.459*** | 0.138 | -0.0282 |
|  | (0.0818) | (0.0608) | (0.0744) | (0.0734) | (0.123) | (0.0632) |
| Constant | 0.126*** | 0.472*** | 0.494*** | 0.518*** | 0.00242 | 3.506*** |
|  | (0.0366) | (0.0454) | (0.0689) | (0.0684) | (0.0446) | (0.194) |
|  |  |  |  |  |  |  |
| Observations | 5,827 | 5,936 | 6,000 | 6,008 | 6,008 | 6,008 |
| R-squared | 0.031 | 0.017 | 0.127 | 0.134 | 0.008 | 0.019 |

Notes: There were differences in how some outcomes were measured at baseline and endline. The baseline numbers on antenatal care visits included visits with both skilled and unskilled providers, whereas the endline included visits with skilled providers only. The list of components used to construct the Empowerment – Participation index (Column 7) also differs between baseline and endline. While the endline measure for Empowerment – vignette (Column 8) is based on a vignette, the baseline measure is based on the response to a question about whether the respondent feels that she has the power to make important decisions that can change the course of her life. For details on the differences, please refer to the questionnaires. Robust standard errors, clustered at the facility level, in parentheses. *** p<0.01, ** p<0.05, * p<0.1

**Table A5.4. Tanzania – Secondary Outcomes**

|  | (1) | (2) | (3) |
| --- | --- | --- | --- |
|  | Low birthweight | Birth preparedness | Maternal depression (K6 score) |
|  |  |  |  |
| Treatment | -0.00552 | 0.00430 | 0.0775 |
|  | (0.00607) | (0.0814) | (0.181) |
| Baseline village-level average | 0.0400 | 0.0440 | 0.0618 |
|  | (0.0325) | (0.0442) | (0.0475) |
| Constant | 0.0509*** | 5.550*** | 17.65*** |
|  | (0.00589) | (0.146) | (0.846) |
|  |  |  |  |
| Observations | 5,886 | 6,008 | 5,859 |
| R-squared | 0.001 | 0.024 | 0.002 |

Notes: Robust standard errors, clustered at the facility-level, in parentheses. *** p<0.01, ** p<0.05, * p<0.1

**Appendix A6. Impact on Intermediate Outcomes – Indonesia**

The intermediate outcomes draw from both the household survey and the health facility survey. Outcomes from the former were analyzed for a sample of 6,001 households from 200 villages, while those from the latter were analyzed for 200 health facilities. These outcomes are grouped by the types of healthcare-related activities organized by T4D participants, as listed in Table 4. The definitions of these outcomes are in Appendix A3.

Note that all outcomes presented below were analyzed following Equation (1). In all the following tables, the treatment means are regression adjusted. In the “Impact” column, “***” denotes p<0.01, “**” denotes p<0.05, and “*” denotes p<0.1. The “Effect Size” column reports the coefficient on the treatment dummy divided by the standard deviation of the outcome for the control group.

1. Increased awareness, knowledge and improved community attitudes

|  | **Outcome** | **Treatment Mean** | **Control Mean** | **Impact** | **p-value** | **Effect Size** | **Sample Size** |
| --- | --- | --- | --- | --- | --- | --- | --- |
|  | Knowledge of ANC and facility birth | 2.204 | 2.105 | 0.099* | 0.067 | 0.096 | 5986 |
|  | Knowledge of pregnancy problems | 2.028 | 1.973 | 0.056 | 0.440 | 0.042 | 6001 |
|  | Knowledge of birth preparedness | 1.152 | 1.157 | -0.005 | 0.926 | -0.005 | 6001 |
|  | Knowledge of postnatal complications | 0.835 | 0.800 | 0.035 | 0.290 | 0.049 | 6001 |
|  | Knowledge of T4D levers | 3.048 | 3.045 | 0.003 | 0.918 | 0.004 | 5976 |
|  | Attitudes on partner participation | 3.387 | 3.296 | 0.092*** | 0.003 | 0.126 | 5613 |
|  |  |  |  |  |  |  |  |
|  | Number of Respondents | 3016 | 2985 |  |  |  |  |
|  | Number of villages | 100 | 100 |  |  |  |  |

1. Improved facility access (transportation, new facility, longer facility hours, outreach services)

|  | **Outcome** | **Treatment Mean** | **Control Mean** | **Impact** | **p-value** | **Effect Size** | **Sample Size** |
| --- | --- | --- | --- | --- | --- | --- | --- |
| **Household survey outcomes** | | | | | | | |
|  | Traveled to facility by means other than on foot | 0.924 | 0.914 | 0.009 | 0.494 | 0.033 | 4574 |
|  | Traveled to facility by ambulance | 0.113 | 0.115 | -0.003 | 0.841 | -0.008 | 6001 |
|  | Travel time (hours) | 0.557 | 0.607 | -0.050 | 0.108 | -0.076 | 4458 |
|  | Travel cost (IDR) | 43000.647 | 48038.444 | -5037.797 | 0.274 | -0.049 | 4403 |
|  |  |  |  |  |  |  |  |
|  | Number of Respondents | 3016 | 2985 |  |  |  |  |
|  | Number of villages | 100 | 100 |  |  |  |  |
|  |  |  |  |  |  |  |  |
| **Facility survey outcomes** | | | | | | | |
|  | Mobile clinic serves sample village | 0.699 | 0.677 | 0.022 | 0.737 | 0.047 | 199 |
|  | Mobile clinic serves sample village & setup within past 3 years | 0.200 | 0.152 | 0.049 | 0.374 | 0.135 | 199 |
|  | Number of facilities built in the last 3 years | 0.300 | 0.420 | -0.120 | 0.405 | -0.091 | 200 |
|  | New posyandus have been setup in the last 3 years | 0.198 | 0.270 | -0.072 | 0.229 | -0.161 | 200 |
|  | Number of posyandu located in sample village | 4.323 | 4.820 | -0.497 | 0.261 | -0.117 | 200 |
|  | Facility has functional ambulance | 0.991 | 0.960 | 0.031 | 0.166 | 0.156 | 200 |
|  | Fuel for ambulance available today | 1.001 | 0.969 | 0.032* | 0.082 | 0.182 | 195 |
|  |  |  |  |  |  |  |  |
|  | Number of Respondents | 100 | 100 |  |  |  |  |
|  | Number of villages | 100 | 100 |  |  |  |  |

1. Improved information transparency (cost, opening hours, etc.) or complaint mechanisms

|  | **Outcome** | **Treatment Mean** | **Control Mean** | **Impact** | **p-value** | **Effect Size** | **Sample Size** |
| --- | --- | --- | --- | --- | --- | --- | --- |
|  | Information on cost of delivery services displayed | 1.762 | 1.688 | 0.075 | 0.596 | 0.078 | 189 |
|  | Information on cost of other services displayed | 1.536 | 1.460 | 0.076 | 0.535 | 0.090 | 200 |
|  | Information on facility hours displayed | 1.259 | 1.180 | 0.079 | 0.377 | 0.138 | 200 |
|  | Presence of complaint management system | 1.140 | 1.180 | -0.040 | 0.607 | -0.069 | 200 |
|  | Number of meetings with community members in the past year | 14.497 | 21.860 | -7.363 | 0.314 | -0.098 | 200 |
|  |  |  |  |  |  |  |  |
|  | Number of Respondents | 100 | 100 |  |  |  |  |
|  | Number of villages | 100 | 100 |  |  |  |  |

1. Increased availability of drugs, supplies and other inputs

|  | **Outcome** | **Treatment Mean** | **Control Mean** | **Impact** | **p-value** | **Effect Size** | **Sample Size** |
| --- | --- | --- | --- | --- | --- | --- | --- |
|  | Number of essential medicines for mothers in stock | 10.486 | 10.670 | -0.184 | 0.462 | -0.079 | 200 |
|  | Number of essential medicines for children in stock | 8.084 | 8.130 | -0.046 | 0.758 | -0.047 | 200 |
|  | Number of essential vaccines in stock | 5.711 | 5.850 | -0.139 | 0.131 | -0.304 | 200 |
|  | Number of essential equipment available & functional | 7.871 | 7.900 | -0.029 | 0.547 | -0.081 | 200 |
|  | Number of essential supplies in stock | 13.089 | 13.220 | -0.131 | 0.397 | -0.122 | 200 |
|  |  |  |  |  |  |  |  |
|  | Number of Respondents | 100 | 100 |  |  |  |  |
|  | Number of villages | 100 | 100 |  |  |  |  |

1. Increased ability to pay

|  | **Outcome** | **Treatment Mean** | **Control Mean** | **Impact** | **p-value** | **Effect Size** | **Sample Size** |
| --- | --- | --- | --- | --- | --- | --- | --- |
|  | Cost reported as a barrier to healthcare | 0.061 | 0.065 | -0.004 | 0.729 | -0.018 | 6001 |
|  | Cost of delivery | 578269.053 | 709186.905 | -130917.853** | 0.033 | -0.072 | 5958 |
|  | Paid for delivery (binary) | 0.595 | 0.612 | -0.016 | 0.496 | -0.034 | 5958 |
|  | Insurance enrollment | 0.701 | 0.684 | 0.017 | 0.488 | 0.036 | 5818 |
|  | Savings group membership for MNH care | 0.013 | 0.009 | 0.003 | 0.562 | 0.037 | 5997 |
|  | Used community fund for MNH care | 0.005 | 0.005 | 0.001 | 0.769 | 0.010 | 5999 |
|  |  |  |  |  |  |  |  |
|  | Number of Respondents | 3016 | 2985 |  |  |  |  |
|  | Number of villages | 100 | 100 |  |  |  |  |

1. Improved facility infrastructure

|  | **Outcome** | **Treatment Mean** | **Control Mean** | **Impact** | **p-value** | **Effect Size** | **Sample Size** |
| --- | --- | --- | --- | --- | --- | --- | --- |
|  | Uninterrupted electricity during opening hours in last 7 days | 0.563 | 0.360 | 0.203*** | 0.003 | 0.421 | 200 |
|  | Facility has other sources of electricity besides central grid | 0.769 | 0.840 | -0.071 | 0.212 | -0.192 | 200 |
|  | Facility generator is functional | 0.908 | 0.893 | 0.015 | 0.755 | 0.048 | 160 |
|  | Fuel available for generator | 0.855 | 0.881 | -0.026 | 0.636 | -0.079 | 160 |
|  | Facility solar power system is functional | 1.229 | 0.800 | 0.429 | 0.335 | 0.958 | 7 |
|  | Facility has functional telephone | 0.708 | 0.760 | -0.052 | 0.394 | -0.121 | 200 |
|  | Facility has functional radio | 0.059 | 0.040 | 0.019 | 0.537 | 0.097 | 200 |
|  | Facility has functional email | 0.892 | 0.920 | -0.028 | 0.488 | -0.102 | 200 |
|  | Water outlet available onsite | 0.893 | 0.880 | 0.013 | 0.777 | 0.038 | 200 |
|  | Routine water shortage | 0.239 | 0.340 | -0.101 | 0.119 | -0.213 | 200 |
|  | Duration of water shortage (in days) | 21.775 | 12.676 | 9.099 | 0.259 | 0.289 | 58 |
|  | Facility has specific room for deliveries | 0.920 | 0.950 | -0.030 | 0.388 | -0.138 | 200 |
|  | Privacy level of delivery room (1-4 scale) | 2.479 | 2.760 | -0.282* | 0.059 | -0.273 | 189 |
|  | Number of designated delivery beds | 1.775 | 1.729 | 0.045 | 0.641 | 0.080 | 189 |
|  | Delivery patient toilet - Flushed to septic tank/through piped system | 0.978 | 1.000 | -0.022 | 0.140 |  | 189 |
|  | Delivery patient toilet - Water is available to flush | 0.960 | 0.927 | 0.033 | 0.340 | 0.125 | 189 |
|  | Delivery patient toilet - Water to wash hands | 0.856 | 0.833 | 0.023 | 0.655 | 0.062 | 189 |
|  | Delivery patient toilet - Soap to wash hands | 0.743 | 0.677 | 0.066 | 0.315 | 0.140 | 189 |
|  | Delivery patient toilet - Privacy (1-3 scale) | 1.193 | 1.375 | -0.182*** | 0.010 | -0.332 | 189 |
|  | Toilet inside/next to delivery room | 0.937 | 0.969 | -0.032 | 0.312 | -0.180 | 189 |
|  | Biological/medical waste disposal available | 0.965 | 0.896 | 0.069** | 0.050 | 0.224 | 189 |
|  |  |  |  |  |  |  |  |
|  | Number of Respondents | 100 | 100 |  |  |  |  |
|  | Number of villages | 100 | 100 |  |  |  |  |

1. Increased or improved facility staffing

|  | **Outcome** | **Treatment Mean** | **Control Mean** | **Impact** | **p-value** | **Effect Size** | **Sample Size** |
| --- | --- | --- | --- | --- | --- | --- | --- |
|  | Total number of MNH staff & resident midwives | 30.343 | 30.180 | 0.163 | 0.925 | 0.012 | 200 |
|  | Number of vacancies for medical staff | 1.199 | 1.380 | -0.181 | 0.599 | -0.067 | 200 |
|  | Number of vacancies for non-medical staff | 1.102 | 1.150 | -0.048 | 0.793 | -0.031 | 200 |
|  | Skilled delivery staff present/on call 24*7 | 0.924 | 0.990 | -0.065** | 0.030 | -0.639 | 189 |
|  |  |  |  |  |  |  |  |
|  | Number of Respondents | 100 | 100 |  |  |  |  |
|  | Number of villages | 100 | 100 |  |  |  |  |

1. Bylaws, partnerships, or other interventions aimed at health system uptake

|  | **Outcome** | **Treatment Mean** | **Control Mean** | **Impact** | **p-value** | **Effect Size** | **Sample Size** |
| --- | --- | --- | --- | --- | --- | --- | --- |
|  | Village has by-law on MNH services | 0.091 | 0.110 | -0.019 | 0.654 | -0.061 | 200 |
|  | District Govt./Sub-district Govt./Puskesmas has by-law on MNH services | 0.450 | 0.430 | 0.020 | 0.773 | 0.039 | 200 |
|  | Province/National Govt. has by-law on MNH services | 0.359 | 0.410 | -0.051 | 0.449 | -0.104 | 200 |
|  | Puskesmas has partnership with Baby Dukun | 0.816 | 0.917 | -0.101** | 0.042 | -0.362 | 189 |
|  |  |  |  |  |  |  |  |
|  | Number of Respondents | 100 | 100 |  |  |  |  |
|  | Number of villages | 100 | 100 |  |  |  |  |

1. Improved facility cleanliness

|  | **Outcome** | **Treatment Mean** | **Control Mean** | **Impact** | **p-value** | **Effect Size** | **Sample Size** |
| --- | --- | --- | --- | --- | --- | --- | --- |
| **Household survey outcomes** | | | | | | | |
|  | Recent visit: Cleanliness (1-4 scale) | 1.945 | 1.934 | 0.010 | 0.680 | 0.016 | 5794 |
|  | Sample Puskesmas: Cleanliness (1-4 scale) | 2.048 | 2.036 | 0.012 | 0.710 | 0.018 | 3817 |
|  |  |  |  |  |  |  |  |
|  | Number of Respondents | 3016 | 2985 |  |  |  |  |
|  | Number of villages | 100 | 100 |  |  |  |  |
|  |  |  |  |  |  |  |  |
| **Facility survey outcomes** | | | | | | | |
|  | Delivery bed is clean | 0.924 | 0.885 | 0.038 | 0.367 | 0.119 | 189 |
|  | Delivery room floor is clean | 0.868 | 0.875 | -0.007 | 0.881 | -0.022 | 189 |
|  | Delivery room is well ventilated | 0.912 | 0.948 | -0.036 | 0.342 | -0.160 | 189 |
|  | No dust or mold observed in the room | 0.663 | 0.719 | -0.055 | 0.411 | -0.122 | 189 |
|  | Delivery patient toilet - Cleanliness (1-3 scale) | 1.632 | 1.740 | -0.108 | 0.214 | -0.185 | 189 |
|  |  |  |  |  |  |  |  |
|  | Number of Respondents | 100 | 100 |  |  |  |  |
|  | Number of villages | 100 | 100 |  |  |  |  |

1. Improved (perceived) attitude, effort, trust of the provider

|  | **Outcome** | **Treatment Mean** | **Control Mean** | **Impact** | **p-value** | **Effect Size** | **Sample Size** |
| --- | --- | --- | --- | --- | --- | --- | --- |
| **Household survey outcomes** | | | | | | | |
|  | Recent visit: Satisfaction with waiting time (1-4 scale) | 1.859 | 1.859 | 0.000 | 0.981 | 0.001 | 5796 |
|  | Recent visit: Longest waiting time (hours) | 0.645 | 0.695 | -0.050 | 0.372 | -0.024 | 5796 |
|  | Recent visit: Satisfaction with opening hours (1-4 scale) | 1.805 | 1.814 | -0.009 | 0.680 | -0.015 | 5796 |
|  | Recent visit: Health worker not there (1-4 scale) | 3.602 | 3.514 | 0.0875*** | 0.006 | 0.103 | 5796 |
|  | Recent visit: Respectful treatment (1-4 scale) | 1.862 | 1.871 | -0.009 | 0.715 | -0.014 | 5796 |
|  | Recent visit: Trust nurses/midwives/other staff (1-4 scale) | 1.793 | 1.828 | -0.035 | 0.213 | -0.053 | 5795 |
|  | Recent visit: Availability of drugs/supplies/equipment (1-4 scale) | 2.012 | 2.019 | -0.007 | 0.784 | -0.011 | 5794 |
|  | Recent visit: Quality of physical facility (1-4 scale) | 2.033 | 2.037 | -0.004 | 0.877 | -0.006 | 5796 |
|  | Recent visit: Overall quality of healthcare (1-4 scale) | 1.926 | 1.927 | -0.001 | 0.965 | -0.002 | 5796 |
|  | Recent visit: If people complained, would staff improve | 2.406 | 2.088 | 0.318 | 0.559 | 0.034 | 5420 |
|  | Recent visit: Change in the last year (Better care/Worse/Same) | 1.488 | 1.523 | -0.036 | 0.225 | -0.041 | 5731 |
|  | Sample Puskesmas: Satisfaction with waiting time (1-4 scale) | 2.019 | 2.018 | 0.001 | 0.968 | 0.002 | 3817 |
|  | Sample Puskesmas: Longest waiting time (hours) | 0.912 | 0.949 | -0.037 | 0.710 | -0.012 | 3818 |
|  | Sample Puskesmas: Satisfaction with opening hours (1-4 scale) | 1.841 | 1.835 | 0.006 | 0.846 | 0.009 | 3818 |
|  | Sample Puskesmas: Health worker not there (1-4 scale) | 3.801 | 3.779 | 0.022 | 0.355 | 0.036 | 3818 |
|  | Sample Puskesmas: Respectful treatment (1-4 scale) | 1.978 | 2.035 | -0.057* | 0.051 | -0.084 | 3818 |
|  | Sample Puskesmas: Trust nurses/midwives/other staff (1-4 scale) | 1.892 | 1.930 | -0.037 | 0.230 | -0.056 | 3817 |
|  | Sample Puskesmas: Availability of drugs/supplies/equipment (1-4 scale) | 2.076 | 2.107 | -0.031 | 0.209 | -0.045 | 3817 |
|  | Sample Puskesmas: Quality of physical facility (1-4 scale) | 2.059 | 2.025 | 0.034 | 0.374 | 0.052 | 3818 |
|  | Sample Puskesmas: Overall quality of healthcare (1-4 scale) | 1.986 | 2.001 | -0.015 | 0.565 | -0.025 | 3818 |
|  | Sample Puskesmas: If people complained, would staff improve | 1.156 | 1.163 | -0.007 | 0.728 | -0.012 | 3610 |
|  | Sample Puskesmas: Change in the last year (Better care/Worse/Same) | 1.517 | 1.517 | 0.001 | 0.982 | 0.001 | 3792 |
|  | Non-dignified care | 0.043 | 0.054 | -0.011* | 0.085 | -0.051 | 4472 |
|  | Provider neglect | 0.040 | 0.050 | -0.010 | 0.160 | -0.047 | 4472 |
|  | Provider abuse | 0.000 | 0.001 | -0.001 | 0.149 | -0.029 | 4472 |
|  |  |  |  |  |  |  |  |
|  | Number of Respondents | 3016 | 2985 |  |  |  |  |
|  | Number of villages | 100 | 100 |  |  |  |  |
|  |  |  |  |  |  |  |  |
| **Facility survey outcomes** | | | | | | | |
|  | Healthcare provider perception: Women are well aware of the MNH services offered at [facility name] | 1.530 | 1.530 | 0.000 | 0.999 | 0.000 | 200 |
|  | Healthcare provider perception: Women seeking MNH services understand the problems in running [facility name] | 2.035 | 2.070 | -0.035 | 0.769 | -0.039 | 200 |
|  | Healthcare provider perception: When [facility name] is making a decision about MNH, community members are consulted | 1.291 | 1.460 | -0.169* | 0.078 | -0.219 | 199 |
|  | Healthcare provider perception: In general, maternal health patients do not have meaningful feedback | 3.184 | 3.110 | 0.074 | 0.539 | 0.087 | 200 |
|  | Healthcare provider perception: Most patients don’t appreciate the effort put in by the staff | 3.238 | 3.150 | 0.088 | 0.517 | 0.089 | 200 |
|  | Healthcare provider perception: Patients communicate about their feedback on MNH services | 1.390 | 1.490 | -0.100 | 0.328 | -0.123 | 200 |
|  | Midwife assigned to sample village | 0.980 | 0.960 | 0.020 | 0.401 | 0.103 | 200 |
|  | Assigned midwife lives in sample village | 0.817 | 0.885 | -0.068 | 0.179 | -0.213 | 194 |
|  | Midwife receives free housing in village | 0.393 | 0.542 | -0.148** | 0.025 | -0.296 | 194 |
|  |  |  |  |  |  |  |  |
|  | Number of Respondents | 100 | 100 |  |  |  |  |
|  | Number of villages | 100 | 100 |  |  |  |  |

1. Improved provider knowledge

|  | **Outcome** | **Treatment Mean** | **Control Mean** | **Impact** | **p-value** | **Effect Size** | **Sample Size** |
| --- | --- | --- | --- | --- | --- | --- | --- |
|  | Provider responded correctly to ANC question | 0.960 | 0.930 | 0.030 | 0.367 | 0.115 | 200 |
|  | Provider responded correctly to delivery question | 0.980 | 0.950 | 0.030 | 0.246 | 0.138 | 200 |
|  | Number of reasons to refer woman to hospital for birth | 1.398 | 1.400 | -0.002 | 0.989 | -0.002 | 200 |
|  |  |  |  |  |  |  |  |
|  | Number of Respondents | 100 | 100 |  |  |  |  |
|  | Number of villages | 100 | 100 |  |  |  |  |

**Appendix A7. Impact on Intermediate Outcomes – Tanzania**

The intermediate outcomes draw from both the household survey and the health facility survey. Outcomes from the former were analyzed for a sample of 6,008 households from 200 villages. Outcomes from the latter were analyzed for 153 facilities. Further, among the facility survey outcomes, those with the prefix “Facility” were analyzed for 153 sample facilities, while those with the prefix “Village” were analyzed for the 200 sample villages in the catchment areas of these 153 facilities.^[[29]](#footnote-29)^ These outcomes are grouped by the types of healthcare-related activities organized by T4D participants, as listed in Table 5. The definitions of these outcomes are in Appendix A3.

Note that all outcomes presented below were analyzed following Equation (1). In all the following tables, the treatment means are regression adjusted. In the “Impact” column, “***” denotes p<0.01, “**” denotes p<0.05, and “*” denotes p<0.1. The “Effect Size” column reports the coefficient on the treatment dummy divided by the standard deviation of outcome for the control group.

1. Increased awareness, knowledge and improved community attitudes

|  | **Outcome** | **Treatment Mean** | **Control Mean** | **Impact** | **p-value** | **Effect Size** | **Sample Size** |
| --- | --- | --- | --- | --- | --- | --- | --- |
|  | Knowledge of ANC and facility birth | 2.876 | 2.852 | 0.023 | 0.185 | 0.051 | 5984 |
|  | Knowledge of pregnancy problems | 2.401 | 2.364 | 0.037 | 0.689 | 0.020 | 5944 |
|  | Knowledge of birth preparedness | 1.433 | 1.381 | 0.053 | 0.392 | 0.044 | 5944 |
|  | Knowledge of postnatal complications | 1.155 | 1.144 | 0.011 | 0.758 | 0.013 | 6008 |
|  | Knowledge of T4D levers | 3.052 | 3.074 | -0.023 | 0.595 | -0.026 | 6005 |
|  | Attitudes on partner participation | 2.333 | 2.328 | 0.004 | 0.898 | 0.005 | 5981 |
|  |  |  |  |  |  |  |  |
|  | Number of Respondents | 2971 | 3037 |  |  |  |  |
|  | Number of villages | 100 | 100 |  |  |  |  |

1. Improved facility access (transportation, new facility, longer facility hours, outreach services)

|  | **Outcome** | **Treatment Mean** | **Control Mean** | **Impact** | **p-value** | **Effect Size** | **Sample Size** |
| --- | --- | --- | --- | --- | --- | --- | --- |
| **Household survey outcomes** | | | | | | | |
|  | Traveled to facility by means other than on foot | 0.678 | 0.713 | -0.035 | 0.301 | -0.078 | 4036 |
|  | Travel time (hours) | 1.239 | 1.255 | -0.016 | 0.795 | -0.012 | 3830 |
|  | Travel cost (TZS) | 5200.149 | 4963.006 | 237.143 | 0.634 | 0.021 | 3955 |
|  |  |  |  |  |  |  |  |
|  | Number of Respondents | 2971 | 3037 |  |  |  |  |
|  | Number of villages | 100 | 100 |  |  |  |  |
|  |  |  |  |  |  |  |  |
| **Facility survey outcomes** | | | | | | | |
|  | **Outcome** | **Treatment Mean** | **Control Mean** | **Impact** | **p-value** | **Effect Size** | **Sample Size** |
|  | Facility has functional ambulance | 0.043 | 0.065 | -0.022 | 0.557 | -0.089 | 152 |
|  | Facility has access to vehicle for emergencies | 0.910 | 0.909 | 0.001 | 0.981 | 0.004 | 152 |
|  | Village served by mobile clinic | 0.547 | 0.670 | -0.123* | 0.080 | -0.261 | 198 |
|  | Village served by mobile clinic setup within past 3 years | 0.342 | 0.450 | -0.108 | 0.120 | -0.217 | 198 |
|  | Village has health dispensary | 0.680 | 0.674 | 0.006 | 0.922 | 0.013 | 186 |
|  |  |  |  |  |  |  |  |
|  | Number of facilities | 76 | 77 |  |  |  |  |
|  | Number of villages | 100 | 100 |  |  |  |  |

1. Improved information transparency (cost, opening hours, etc.) or complaint mechanisms

|  | **Outcome** | **Treatment Mean** | **Control Mean** | **Impact** | **p-value** | **Effect Size** | **Sample Size** |
| --- | --- | --- | --- | --- | --- | --- | --- |
|  | Facility: Information on cost of delivery services displayed | 0.330 | 0.527 | -0.197** | 0.016 | -0.392 | 141 |
|  | Facility: Information on cost of other services displayed | 0.564 | 0.701 | -0.137* | 0.077 | -0.298 | 152 |
|  | Facility: Information on facility hours displayed | 0.776 | 0.870 | -0.094 | 0.128 | -0.277 | 152 |
|  | Facility: Presence of complaint management system | 0.916 | 0.857 | 0.059 | 0.249 | 0.166 | 152 |
|  | Facility: Number of meetings with community members in the past year | 2.020 | 2.610 | -0.591 | 0.311 | -0.134 | 152 |
|  |  |  |  |  |  |  |  |
|  | Number of facilities | 76 | 77 |  |  |  |  |
|  | Number of villages | 100 | 100 |  |  |  |  |

1. Bylaws, partnerships, or other interventions aimed at health system uptake

|  | **Outcome** | **Treatment Mean** | **Control Mean** | **Impact** | **p-value** | **Effect Size** | **Sample Size** |
| --- | --- | --- | --- | --- | --- | --- | --- |
|  | Facility: Health center/dispensary has by-law on MNH services | 0.201 | 0.156 | 0.045 | 0.468 | 0.124 | 152 |
|  | Facility: Dispensary has partnership with TBAs | 0.818 | 0.662 | 0.155** | 0.019 | 0.326 | 152 |
|  | Facility: Presence of programs/campaigns to educate TBAs | 0.580 | 0.558 | 0.021 | 0.781 | 0.043 | 152 |
|  | Village has by-law on MNH services | 0.148 | 0.170 | -0.022 | 0.628 | -0.059 | 198 |
|  |  |  |  |  |  |  |  |
|  | Number of facilities | 76 | 77 |  |  |  |  |
|  | Number of villages | 100 | 100 |  |  |  |  |

1. Increased ability to pay

|  | **Outcome** | **Treatment Mean** | **Control Mean** | **Impact** | **p-value** | **Effect Size** | **Sample Size** |
| --- | --- | --- | --- | --- | --- | --- | --- |
|  | Cost reported as a barrier to healthcare | 0.018 | 0.022 | -0.004 | 0.410 | -0.030 | 6008 |
|  | Cost of delivery | 5297.209 | 6075.415 | -778.205 | 0.547 | -0.020 | 5839 |
|  | Paid for delivery (binary) | 0.323 | 0.309 | 0.014 | 0.582 | 0.031 | 5839 |
|  | Insurance enrollment and usage | 0.058 | 0.054 | 0.004 | 0.673 | 0.016 | 5889 |
|  | Savings group membership for MNH care | 0.044 | 0.044 | 0.000 | 0.977 | -0.001 | 6006 |
|  | Used community fund for MNH care | 0.011 | 0.010 | 0.001 | 0.655 | 0.013 | 6002 |
|  |  |  |  |  |  |  |  |
|  | Number of Respondents | 2971 | 3037 |  |  |  |  |
|  | Number of villages | 100 | 100 |  |  |  |  |

1. Improved attitude, effort, trust of the provider

|  | **Outcome** | **Treatment Mean** | **Control Mean** | **Impact** | **p-value** | **Effect Size** | **Sample Size** |
| --- | --- | --- | --- | --- | --- | --- | --- |
| **Household survey outcomes** | | | | | | | |
|  | Recent visit: Satisfaction with waiting time (1-4 scale) | 2.679 | 2.626 | 0.053 | 0.282 | 0.063 | 5448 |
|  | Recent visit: Longest waiting time (hours) | 2.351 | 2.445 | -0.094 | 0.451 | -0.047 | 5449 |
|  | Recent visit: Satisfaction with opening hours (1-4 scale) | 2.865 | 2.823 | 0.042 | 0.324 | 0.050 | 5443 |
|  | Recent visit: Health worker not there (1-4 scale) | 3.553 | 3.585 | -0.032 | 0.479 | -0.037 | 5449 |
|  | Recent visit: Respectful treatment (1-4 scale) | 2.868 | 2.839 | 0.029 | 0.415 | 0.040 | 5398 |
|  | Recent visit: Ever refused treatment (1-4 scale) | 3.828 | 3.824 | 0.005 | 0.845 | 0.008 | 5449 |
|  | Recent visit: Trust nurses/midwives/other staff (1-4 scale) | 2.950 | 2.928 | 0.022 | 0.565 | 0.030 | 5440 |
|  | Recent visit: Availability of drugs/supplies/equipment (1-4 scale) | 2.361 | 2.308 | 0.053 | 0.273 | 0.061 | 5442 |
|  | Recent visit: Staff communication skills (1-4 scale) | 2.710 | 2.674 | 0.037 | 0.387 | 0.050 | 5446 |
|  | Recent visit: Quality of physical facility (1-4 scale) | 2.657 | 2.674 | -0.018 | 0.593 | -0.026 | 5373 |
|  | Recent visit: Overall quality of healthcare (1-4 scale) | 2.710 | 2.718 | -0.008 | 0.819 | -0.011 | 5401 |
|  | Recent visit: If people complained, would staff improve | 0.644 | 0.642 | 0.002 | 0.919 | 0.005 | 4847 |
|  | Recent visit: Change in the last year (Better care/Same/Worse) | 2.367 | 2.372 | -0.005 | 0.866 | -0.009 | 5343 |
|  | Recent visit: Greeted promptly | 0.551 | 0.523 | 0.028 | 0.264 | 0.056 | 5449 |
|  | Recent visit: Insufficient seating | 0.254 | 0.260 | -0.006 | 0.787 | -0.013 | 5449 |
|  | Recent visit: Staff understood reason for visit | 0.538 | 0.513 | 0.025 | 0.209 | 0.049 | 5449 |
|  | Recent visit: Staff used bad language | 0.093 | 0.110 | -0.017 | 0.165 | -0.054 | 5449 |
|  | Recent visit: Properly informed | 0.390 | 0.379 | 0.011 | 0.673 | 0.023 | 5449 |
|  | Recent visit: Not given options for treatment | 0.230 | 0.236 | -0.007 | 0.632 | -0.016 | 5449 |
|  | Sample Dispensary: Satisfaction with waiting time (1-4 scale) | 2.672 | 2.643 | 0.029 | 0.596 | 0.034 | 4672 |
|  | Sample Dispensary: Longest waiting time (hours) | 2.343 | 2.384 | -0.041 | 0.770 | -0.020 | 4674 |
|  | Sample Dispensary: Satisfaction with opening hours (1-4 scale) | 2.842 | 2.813 | 0.029 | 0.538 | 0.035 | 4671 |
|  | Sample Dispensary: Health worker not there (1-4 scale) | 3.516 | 3.562 | -0.046 | 0.376 | -0.053 | 4674 |
|  | Sample Dispensary: Respectful treatment (1-4 scale) | 2.849 | 2.824 | 0.025 | 0.538 | 0.034 | 4628 |
|  | Sample Dispensary: Ever refused treatment (1-4 scale) | 3.811 | 3.812 | 0.000 | 0.988 | -0.001 | 4674 |
|  | Sample Dispensary: Trust nurses/midwives/other staff (1-4 scale) | 2.926 | 2.921 | 0.005 | 0.912 | 0.006 | 4667 |
|  | Sample Dispensary: Availability of drugs/supplies/equipment (1-4 scale) | 2.327 | 2.292 | 0.035 | 0.550 | 0.040 | 4667 |
|  | Sample Dispensary: Staff communication skills (1-4 scale) | 2.693 | 2.666 | 0.027 | 0.571 | 0.036 | 4671 |
|  | Sample Dispensary: Quality of physical facility (1-4 scale) | 2.634 | 2.676 | -0.042 | 0.277 | -0.062 | 4674 |
|  | Sample Dispensary: Overall quality of healthcare (1-4 scale) | 2.630 | 2.648 | -0.018 | 0.622 | -0.025 | 4632 |
|  | Sample Dispensary: If people complained, would staff improve | 0.641 | 0.639 | 0.002 | 0.926 | 0.005 | 4183 |
|  | Sample Dispensary: Change in the last year (Better care/Same/Worse) | 2.364 | 2.369 | -0.005 | 0.875 | -0.009 | 4599 |
|  | Sample Dispensary: Greeted promptly | 0.550 | 0.526 | 0.024 | 0.375 | 0.048 | 4674 |
|  | Sample Dispensary: Insufficient seating | 0.257 | 0.247 | 0.010 | 0.667 | 0.024 | 4674 |
|  | Sample Dispensary: Staff understood reason for visit | 0.523 | 0.506 | 0.017 | 0.418 | 0.033 | 4674 |
|  | Sample Dispensary: Staff used bad language | 0.101 | 0.113 | -0.011 | 0.414 | -0.036 | 4674 |
|  | Sample Dispensary: Properly informed | 0.390 | 0.373 | 0.016 | 0.546 | 0.034 | 4674 |
|  | Sample Dispensary: Not given options for treatment | 0.236 | 0.232 | 0.004 | 0.798 | 0.010 | 4674 |
|  | Non-dignified care | 0.121 | 0.101 | 0.020* | 0.063 | 0.067 | 4015 |
|  | Provider neglect | 0.061 | 0.050 | 0.012 | 0.107 | 0.053 | 4012 |
|  | Provider abuse | 0.023 | 0.015 | 0.008 | 0.104 | 0.063 | 4001 |
|  |  |  |  |  |  |  |  |
|  | Number of Respondents | 2971 | 3037 |  |  |  |  |
|  | Number of villages | 100 | 100 |  |  |  |  |
|  |  |  |  |  |  |  |  |
| **Facility survey outcomes** | | | | | | | |
|  | Facility (Healthcare provider perception): Women are well aware of the MNH services offered at [dispensary name] | 3.479 | 3.221 | 0.259 | 0.146 | 0.216 | 151 |
|  | Facility (Healthcare provider perception): Women seeking MNH understand the problems in running [dispensary name] | 2.892 | 2.688 | 0.204 | 0.308 | 0.164 | 151 |
|  | Facility (Healthcare provider perception): Community members consulted when [dispensary] makes a decision on MNH | 3.202 | 2.805 | 0.396** | 0.028 | 0.326 | 151 |
|  | Facility (Healthcare provider perception): In general, maternal health patients do not have meaningful feedback | 2.845 | 3.117 | -0.271 | 0.147 | -0.250 | 151 |
|  | Facility (Healthcare provider perception): Most patients don’t appreciate the effort put in by the staff | 2.555 | 2.857 | -0.302 | 0.119 | -0.264 | 151 |
|  | Facility (Healthcare provider perception): Patients communicate about their feedback on MNH services | 2.851 | 2.844 | 0.007 | 0.971 | 0.006 | 151 |
|  | Village: Whether CHW assigned to sample village | 0.682 | 0.610 | 0.072 | 0.288 | 0.147 | 198 |
|  | Village: Number of CHWs assigned to sample village | 1.387 | 1.444 | -0.057 | 0.810 | -0.026 | 194 |
|  | Village: Whether assigned CHWs live in village | 0.650 | 0.590 | 0.060 | 0.396 | 0.121 | 197 |
|  |  |  |  |  |  |  |  |
|  | Number of facilities | 76 | 77 |  |  |  |  |
|  | Number of villages | 100 | 100 |  |  |  |  |

1. Increased or improved facility staffing

|  | **Outcome** | **Treatment Mean** | **Control Mean** | **Impact** | **p-value** | **Effect Size** | **Sample Size** |
| --- | --- | --- | --- | --- | --- | --- | --- |
|  | Number of paid medical staff supposed to work at facility | 3.645 | 4.273 | -0.628 | 0.112 | -0.226 | 152 |
|  | Number of paid medical staff who actually work at facility | 3.197 | 3.169 | 0.028 | 0.922 | 0.016 | 152 |
|  | Number of volunteers who work at facility | 1.257 | 1.182 | 0.075 | 0.785 | 0.033 | 152 |
|  | Whether there are any staff vacancies | 0.787 | 0.753 | 0.034 | 0.625 | 0.079 | 152 |
|  | Number of staff vacancies | 2.388 | 2.403 | -0.015 | 0.967 | -0.006 | 152 |
|  | Skilled delivery staff present/on call 24*7 | 0.870 | 0.974 | -0.104** | 0.019 | -0.652 | 152 |
|  | Facility staff can be contacted by cellphone | 0.726 | 0.714 | 0.012 | 0.871 | 0.027 | 152 |
|  |  |  |  |  |  |  |  |
|  | Number of facilities | 76 | 77 |  |  |  |  |
|  | Number of villages | 100 | 100 |  |  |  |  |

1. Improved facility infrastructure

|  | **Outcome** | **Treatment Mean** | **Control Mean** | **Impact** | **p-value** | **Effect Size** | **Sample Size** |
| --- | --- | --- | --- | --- | --- | --- | --- |
|  | Facility is connected to the central electricity grid | 0.452 | 0.455 | -0.003 | 0.973 | -0.005 | 152 |
|  | Facility had uninterrupted electricity during opening hours in last 7 days | 0.372 | 0.390 | -0.017 | 0.822 | -0.035 | 152 |
|  | Facility has other sources of electricity besides central grid | 0.793 | 0.818 | -0.025 | 0.700 | -0.065 | 152 |
|  | Facility solar power system is functional | 0.567 | 0.623 | -0.056 | 0.480 | -0.115 | 152 |
|  | Facility has functional telephone | 0.486 | 0.455 | 0.031 | 0.701 | 0.063 | 152 |
|  | Facility has functional radio | 0.029 | 0.000 | 0.029 | 0.148 |  | 152 |
|  | Facility has functional computer | 0.172 | 0.234 | -0.062 | 0.328 | -0.146 | 152 |
|  | Facility has functional email | 0.049 | 0.104 | -0.055 | 0.170 | -0.179 | 152 |
|  | Water outlet available onsite | 0.478 | 0.416 | 0.062 | 0.450 | 0.125 | 152 |
|  | Routine water shortage | 0.505 | 0.481 | 0.024 | 0.770 | 0.048 | 152 |
|  | Duration of water shortage (in days) | 108.292 | 114.324 | -6.033 | 0.798 | -0.056 | 75 |
|  | Facility has specific room for deliveries | 0.987 | 1.000 | -0.013 | 0.325 |  | 152 |
|  | Privacy level of delivery room (1-4 scale) | 2.565 | 2.513 | 0.052 | 0.772 | 0.046 | 148 |
|  | Number of designated delivery beds | 1.741 | 1.805 | -0.065 | 0.691 | -0.054 | 152 |
|  | Facility has maternity home or resting place for women | 1.601 | 1.545 | 0.055 | 0.501 | 0.110 | 152 |
|  | Facility has placenta pit | 0.483 | 0.610 | -0.128 | 0.102 | -0.260 | 152 |
|  | Delivery patient toilet: Flushed to septic tank/through piped system | 0.258 | 0.312 | -0.053 | 0.461 | -0.114 | 152 |
|  | Toilet inside/next to delivery room | 0.341 | 0.532 | -0.191** | 0.017 | -0.381 | 152 |
|  | Delivery patient toilet - Privacy (1-3 scale) | 2.778 | 2.844 | -0.066 | 0.364 | -0.166 | 152 |
|  |  |  |  |  |  |  |  |
|  | Number of facilities | 76 | 77 |  |  |  |  |
|  | Number of villages | 100 | 100 |  |  |  |  |

1. Improved facility cleanliness

|  | **Outcome** | **Treatment Mean** | **Control Mean** | **Impact** | **p-value** | **Effect Size** | **Sample Size** |
| --- | --- | --- | --- | --- | --- | --- | --- |
| **Household survey outcomes** | | | | | | | |
|  | Recent visit: Facility cleanliness (1-4 scale) | 2.873 | 2.871 | 0.003 | 0.925 | 0.004 | 5275 |
|  | Sample Dispensary: Facility cleanliness (1-4 scale) | 2.853 | 2.878 | -0.026 | 0.418 | -0.040 | 4580 |
|  |  |  |  |  |  |  |  |
|  | Number of Respondents | 2971 | 3037 |  |  |  |  |
|  | Number of villages | 100 | 100 |  |  |  |  |
|  |  |  |  |  |  |  |  |
| **Facility survey outcomes** | | | | | | | |
|  | Delivery bed is clean | 0.896 | 0.883 | 0.013 | 0.777 | 0.041 | 151 |
|  | Delivery room floor is clean | 0.975 | 0.870 | 0.105** | 0.014 | 0.311 | 151 |
|  | Delivery room is well ventilated | 0.784 | 0.779 | 0.005 | 0.946 | 0.011 | 151 |
|  | Biological/medical waste disposal available | 0.973 | 0.935 | 0.038 | 0.261 | 0.155 | 151 |
|  | No dust or mold observed in the room | 0.679 | 0.649 | 0.029 | 0.705 | 0.061 | 151 |
|  | Delivery patient toilet - Water is available to flush | 0.496 | 0.675 | -0.180** | 0.023 | -0.381 | 152 |
|  | Delivery patient toilet - Water to wash hands | 0.570 | 0.714 | -0.144* | 0.059 | -0.317 | 152 |
|  | Delivery patient toilet - Soap to wash hands | 0.478 | 0.558 | -0.081 | 0.322 | -0.161 | 152 |
|  | Delivery patient toilet - Cleanliness (1-3 scale) | 2.247 | 2.442 | -0.195* | 0.081 | -0.287 | 152 |
|  |  |  |  |  |  |  |  |
|  | Number of facilities | 76 | 77 |  |  |  |  |
|  | Number of villages | 100 | 100 |  |  |  |  |

1. Increased availability of drugs, supplies and other inputs

|  | **Outcome** | **Treatment Mean** | **Control Mean** | **Impact** | **p-value** | **Effect Size** | **Sample Size** |
| --- | --- | --- | --- | --- | --- | --- | --- |
|  | Number of essential medicines for mothers in stock | 10.258 | 10.104 | 0.154 | 0.709 | 0.056 | 152 |
|  | Number of essential medicines for children in stock | 10.018 | 9.649 | 0.368 | 0.185 | 0.193 | 152 |
|  | Number of essential vaccines in stock | 7.176 | 7.429 | -0.252 | 0.303 | -0.192 | 152 |
|  | Number of essential equipment available & functional | 7.843 | 7.974 | -0.131 | 0.528 | -0.111 | 152 |
|  | Number of essential supplies in stock | 11.374 | 11.468 | -0.093 | 0.758 | -0.048 | 152 |
|  |  |  |  |  |  |  |  |
|  | Number of facilities | 76 | 77 |  |  |  |  |
|  | Number of villages | 100 | 100 |  |  |  |  |

1. Improved provider knowledge

|  | **Outcome** | **Treatment Mean** | **Control Mean** | **Impact** | **p-value** | **Effect Size** | **Sample Size** |
| --- | --- | --- | --- | --- | --- | --- | --- |
|  | Provider responded correctly to question on whether to seek ANC | 0.958 | 0.948 | 0.010 | 0.783 | 0.044 | 151 |
|  | Provider responded correctly to question on ANC timing | 0.947 | 0.909 | 0.038 | 0.362 | 0.133 | 151 |
|  | Provider responded correctly to delivery question | 1.001 | 0.961 | 0.040* | 0.085 | 0.206 | 151 |
|  | Number of reasons given to refer woman to hospital for birth | 3.603 | 3.727 | -0.124 | 0.550 | -0.096 | 151 |
|  | Number of vaccines named that an infant should be given | 5.332 | 5.636 | -0.304** | 0.039 | -0.420 | 151 |
|  |  |  |  |  |  |  |  |
|  | Number of facilities | 76 | 77 |  |  |  |  |
|  | Number of villages | 100 | 100 |  |  |  |  |

**Appendix A8. Sub-group Analyses of Primary Outcomes**

1. Province/region sub-group analysis


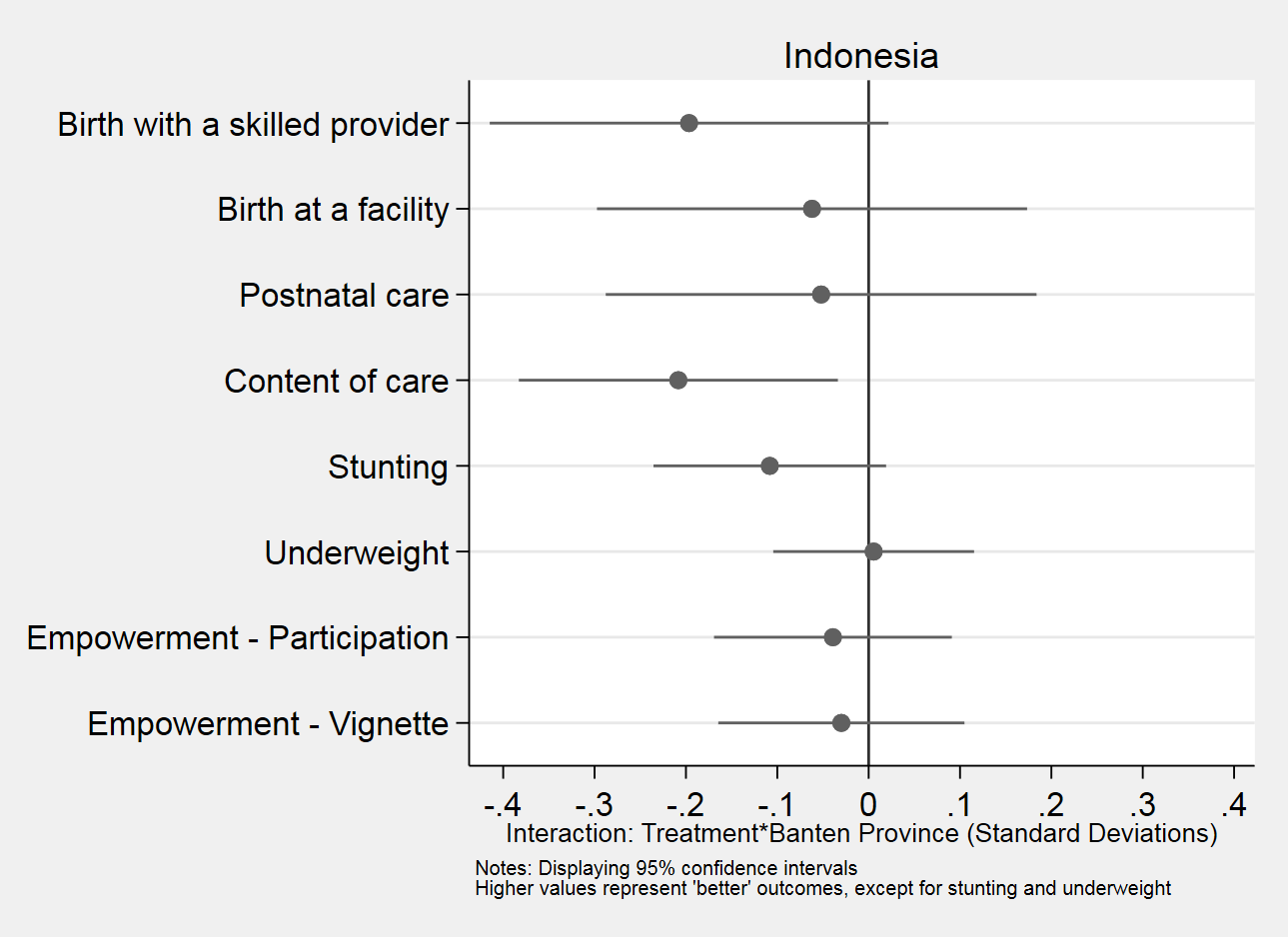


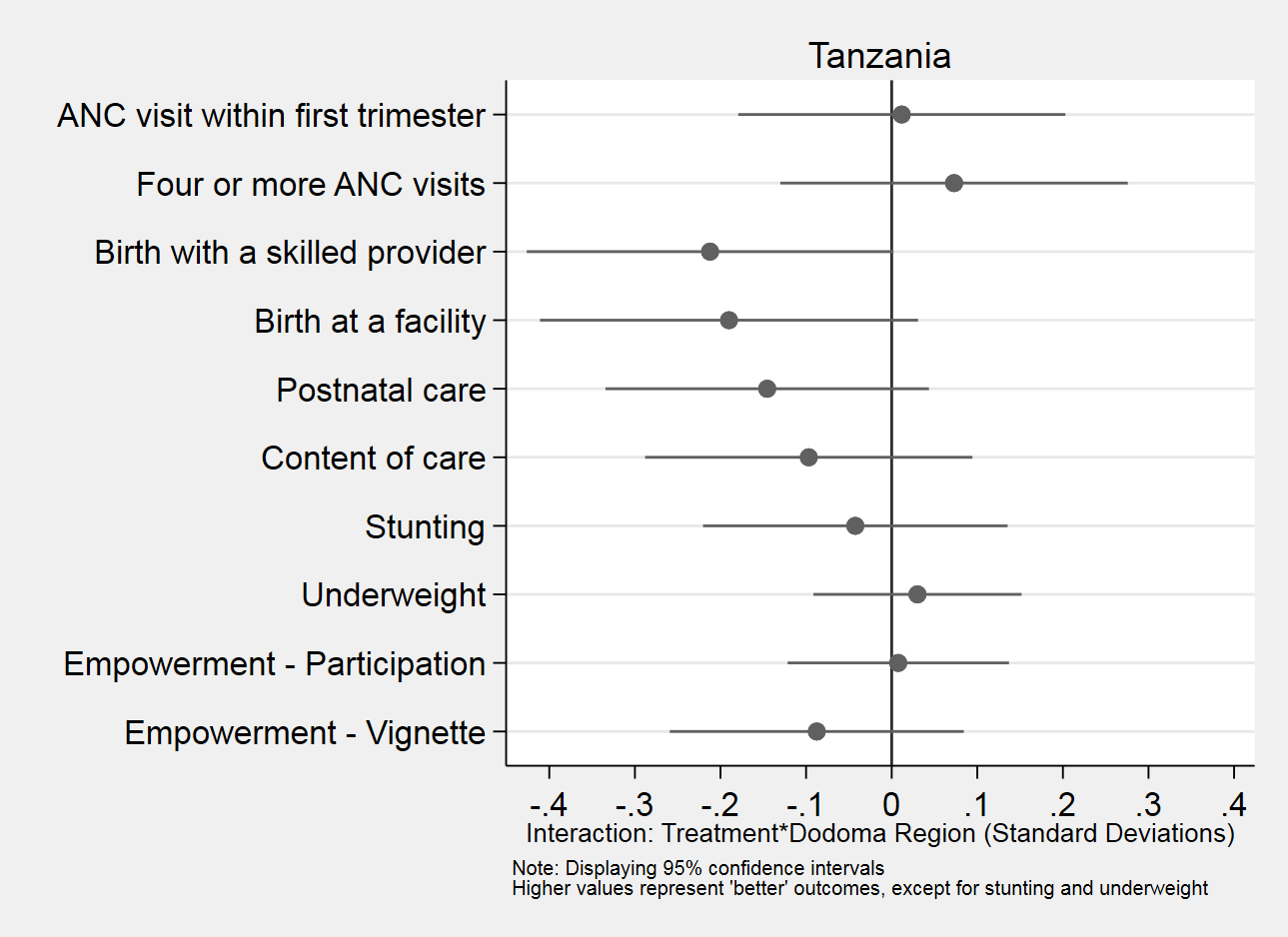


1. Time of birth sub-group analysis


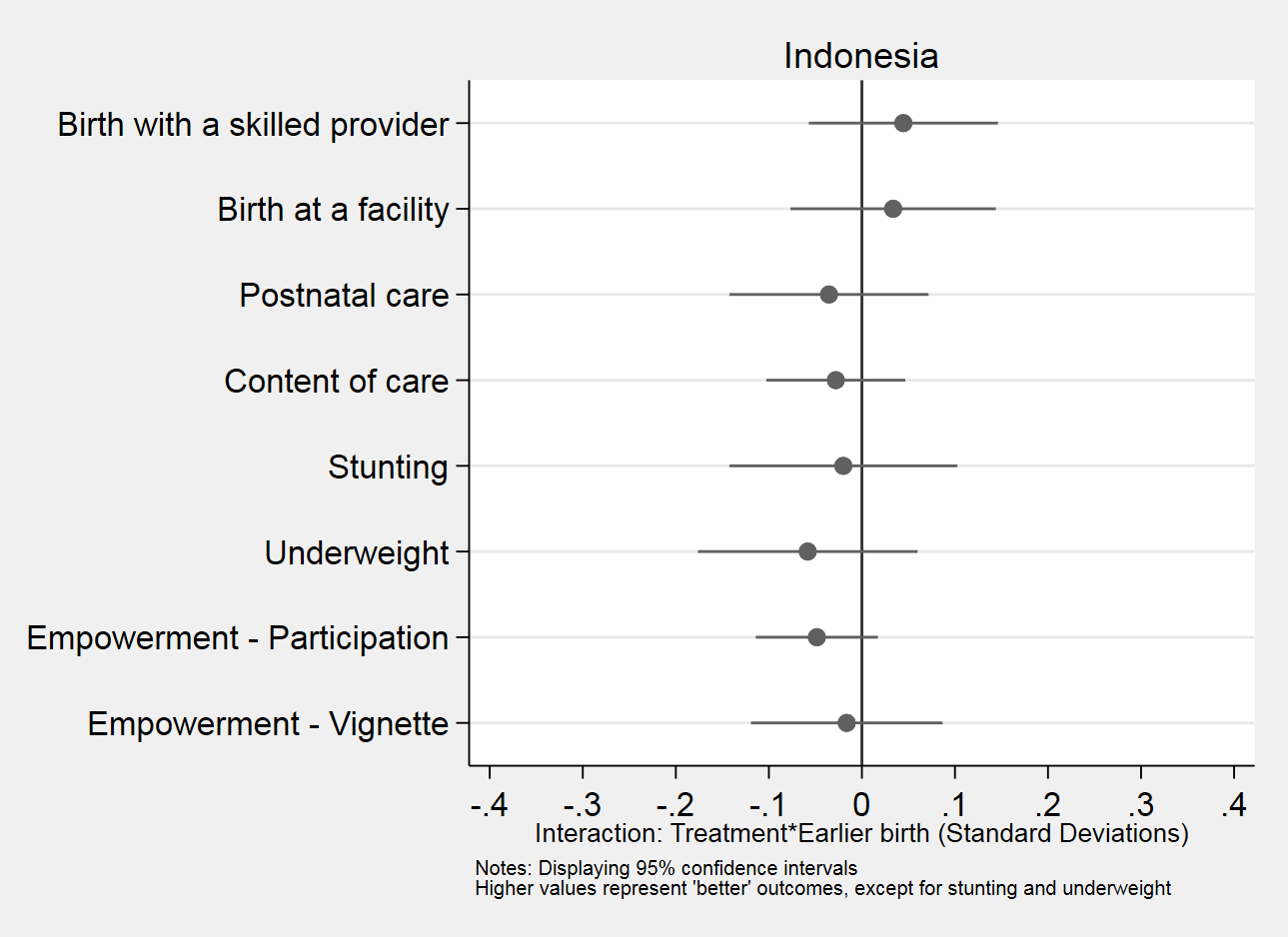


*
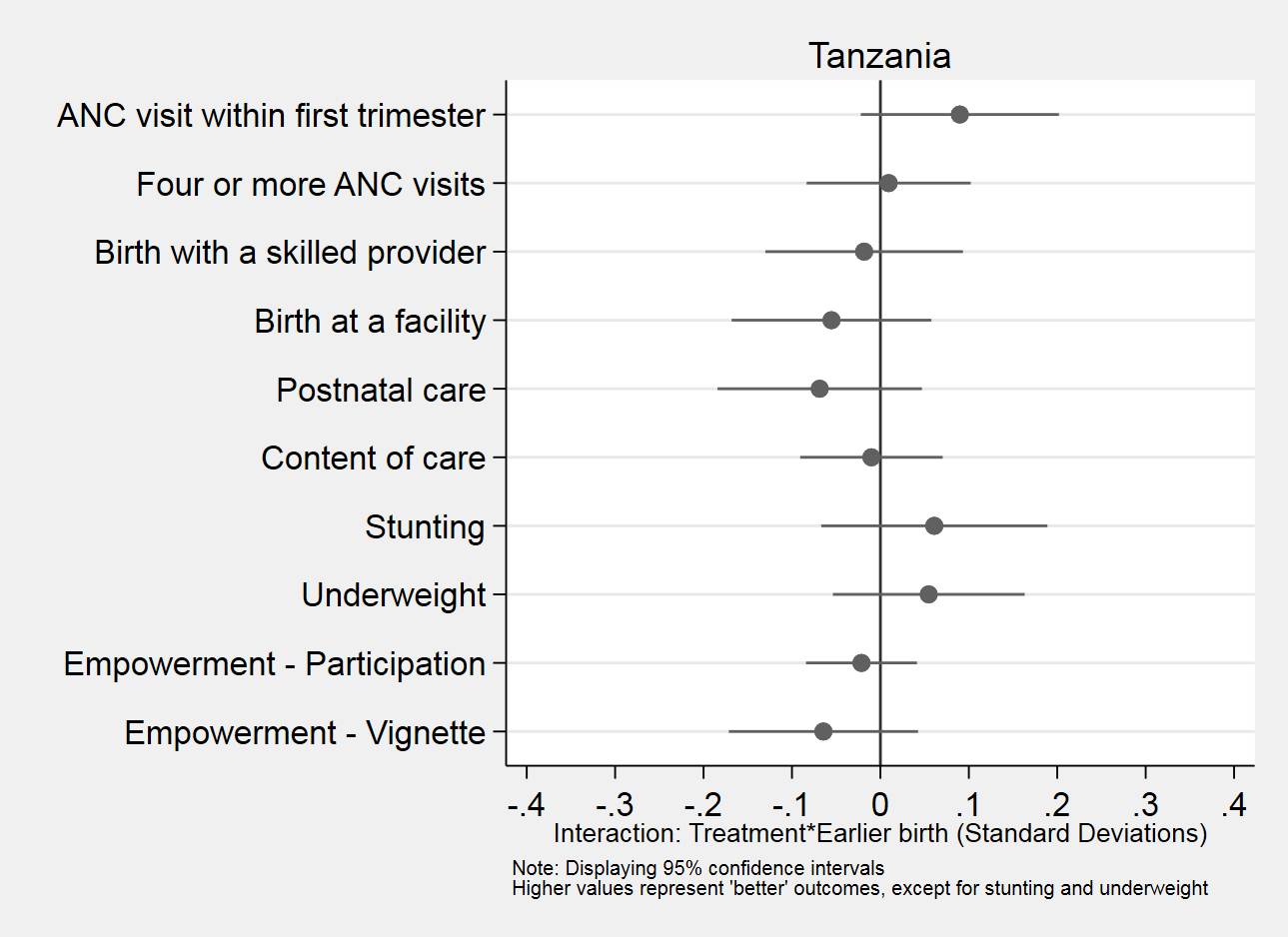
*

1. Facility quality subgroup analysis


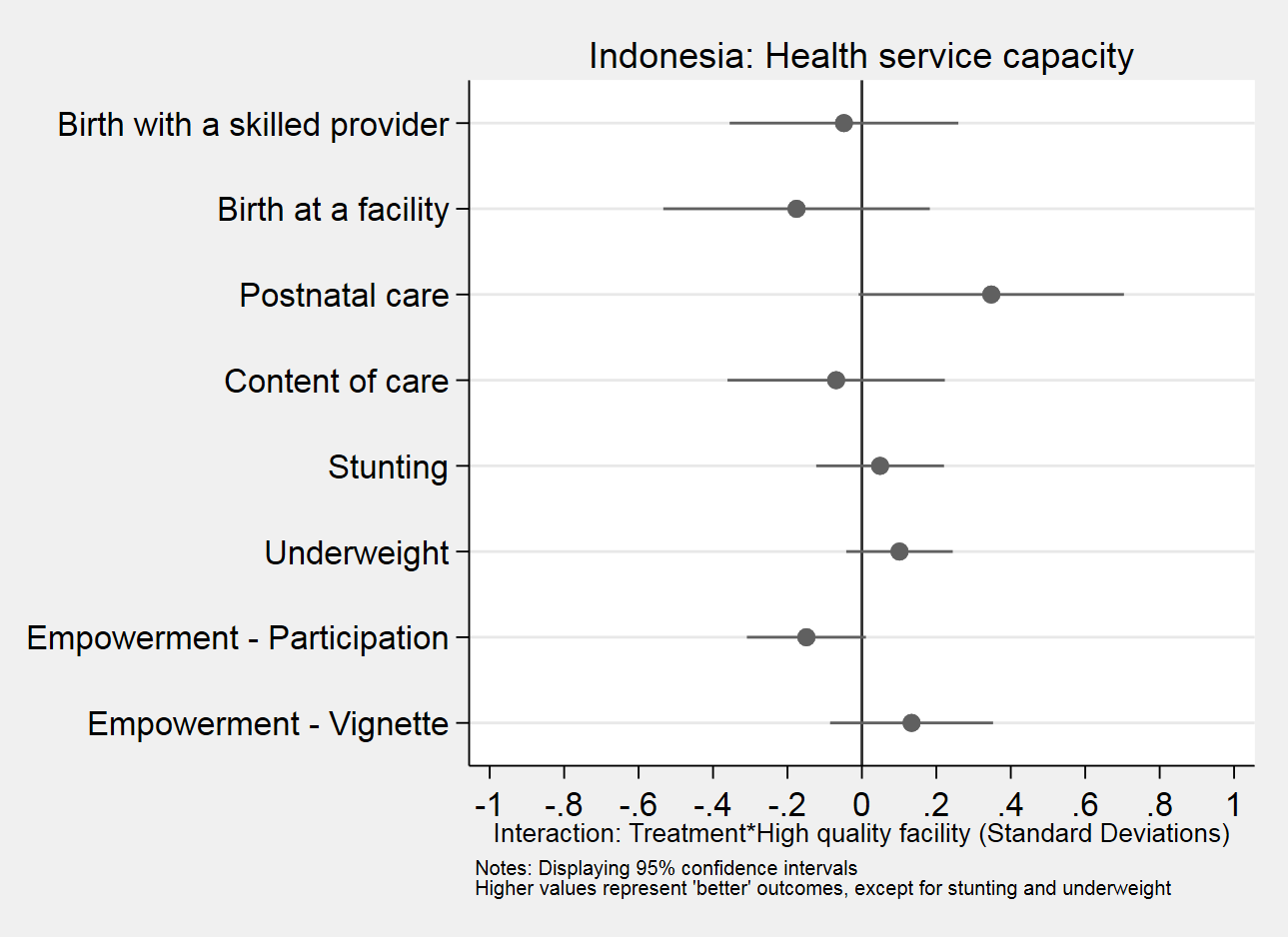


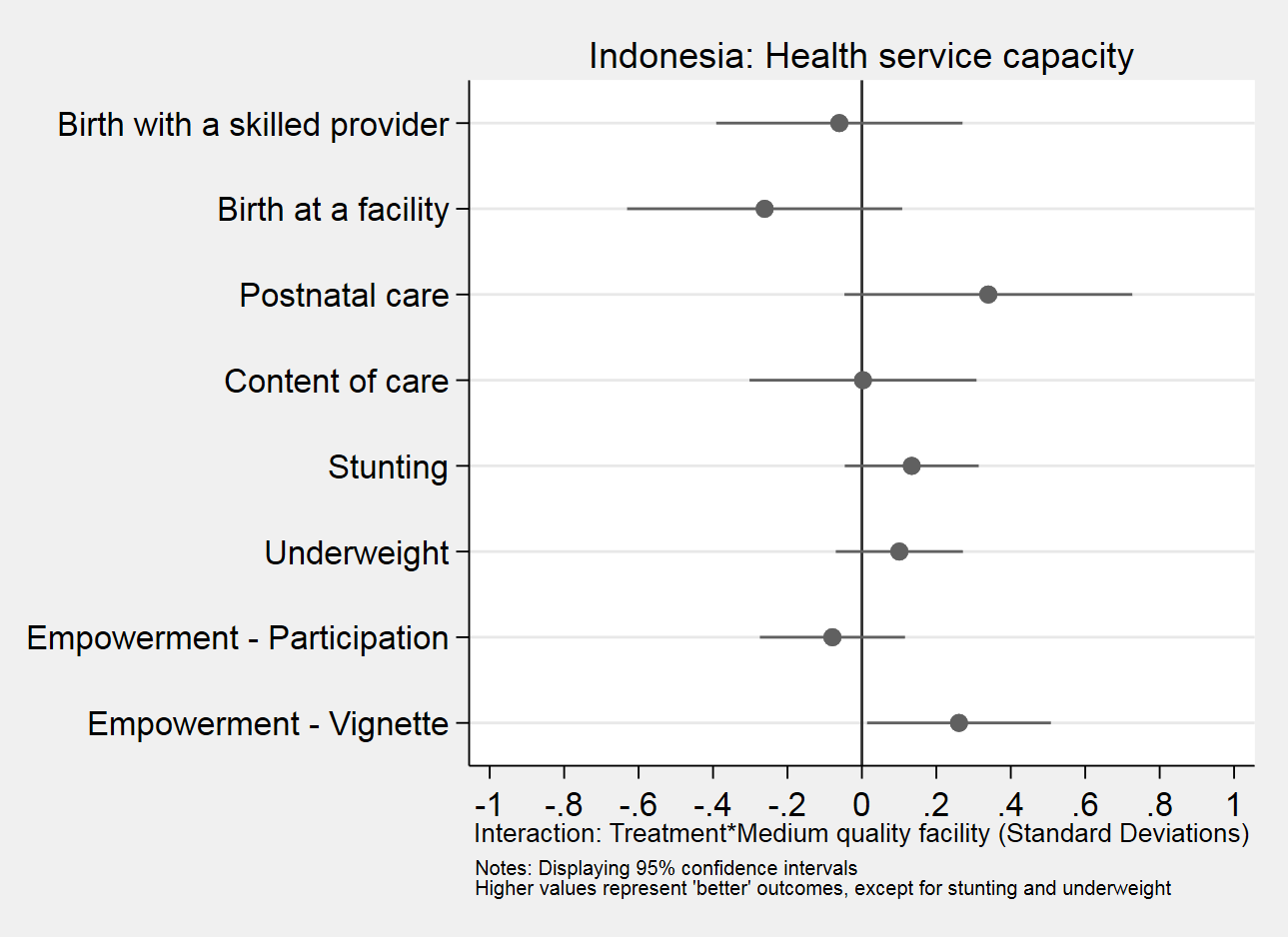


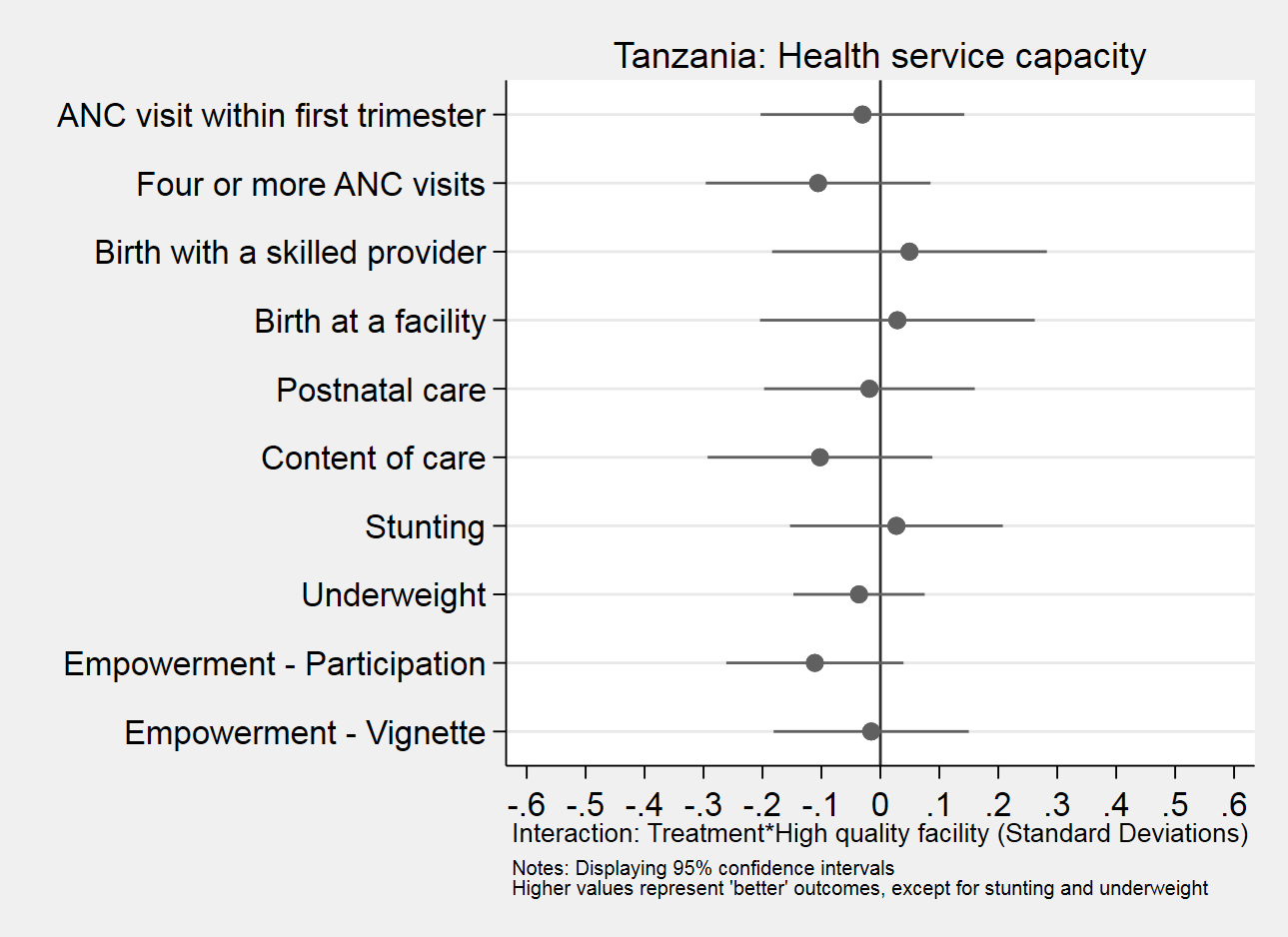


**Appendix A9. Primary Outcomes: Detectable Differences**

**Table A9.1. Indonesia**

| **Outcome** | **Endline Mean** | **Detectable Difference** | **Detectable Difference (Std. Dev.)** |
| --- | --- | --- | --- |
| Birth with a skilled provider | 0.856 | 0.066 | 0.193 |
| Birth at a facility | 0.743 | 0.097 | 0.221 |
| Postnatal care | 0.583 | 0.089 | 0.181 |
| Stunting | 0.163 | 0.040 | 0.110 |
| Underweight | 0.131 | 0.028 | 0.083 |
| Empowerment - Vignette | 1.568 | 0.092 | 0.106 |
| Empowerment - Participation | 0.008 | 0.094 | 0.094 |
| Content of care | 0.023 | 0.141 | 0.141 |

Notes: Detectable difference calculated for the clustered randomization using Endline household survey data. The calculation assumes that power=0.8, alpha=0.05, that the standard deviation is the same in both the treatment and control groups, and that the clusters are of varying sizes.

**Table A9.2. Tanzania**

| **Outcome** | **Endline Mean** | **Detectable Difference** | **Detectable Difference (Std. Dev.)** |
| --- | --- | --- | --- |
| ANC visit within first trimester | 0.227 | 0.054 | 0.131 |
| Four or more ANC visits | 0.523 | 0.064 | 0.128 |
| Birth with a skilled provider | 0.680 | 0.093 | 0.198 |
| Birth at a facility | 0.672 | 0.096 | 0.204 |
| Postnatal care | 0.315 | 0.068 | 0.147 |
| Stunting | 0.287 | 0.056 | 0.126 |
| Underweight | 0.072 | 0.021 | 0.081 |
| Empowerment - Vignette | 3.181 | 0.146 | 0.133 |
| Empowerment - Participation | 0.013 | 0.097 | 0.097 |
| Content of care | -0.007 | 0.140 | 0.140 |

Notes: Detectable difference calculated for the clustered randomization using Endline household survey data. The calculation assumes that power=0.8, alpha=0.05, that the standard deviation is the same in both the treatment and control groups, and that the clusters are of varying sizes.

1. Calculated as within 13 weeks of the pregnancy for Indonesia, where response was given in weeks [↑](#footnote-ref-1)
2. Calculated taking the mother's estimate, or where this conflicted with the card estimate, an average of the mother and the card estimates given [↑](#footnote-ref-2)
3. The DHS for Indonesia defines skilled attendant as including doctors, nurses and midwives, including village midwife [↑](#footnote-ref-3)
4. Received two or more physical checks for danger signs with a skilled attendant within 7 days; received required vaccinations with a skilled attendant within 7 days; received at least one supplement with a skilled attendant within 7 days. [↑](#footnote-ref-4)
5. Each baby from a multiple birth is included individually. [↑](#footnote-ref-5)
6. Ibid. [↑](#footnote-ref-6)
7. Ibid. [↑](#footnote-ref-7)
8. Each baby from a multiple birth is included individually. Weight-for-age is measured as proportion of infants (for whom T4D has valid weight measurements) who are below 2 standard deviations from the median WHO Child Growth Standards. [↑](#footnote-ref-8)
9. Each baby from a multiple birth is included individually. Length-for-age is measured as proportion of infants (for whom T4D has valid length measurements) who are below 2 standard deviations from the median WHO Child Growth Standards. [↑](#footnote-ref-9)
10. Calculated taking the mother's estimate, or where this conflicted with the card estimate, an average of the mother and the card estimates given [↑](#footnote-ref-10)
11. At least 3 of following preparations: planned where would have the baby before birth; made arrangements for transport before birth; decided who would assist delivery before birth; decided how to pay for delivery before birth; identified potential blood donors before birth; identified childcare options before birth; prepared materials [↑](#footnote-ref-11)
12. Tanzania - required vaccinations BCG, Polio and DPT-HB. Indonesia required vaccinations HB0; In Tanzania this was asked about all babies (separately for multiple births) but only the first child listed is included here (i.e. one per birth) [↑](#footnote-ref-12)
13. In Tanzania this was asked about all babies (separately for multiple births) but only the first child listed is included here (i.e. one per birth) [↑](#footnote-ref-13)
14. Received any postnatal care (skilled or unskilled attendant); received any postnatal care with a skilled attendant within 7 days; received two or more physical checks for danger signs with a skilled attendant within 7 days; received required vaccinations with a skilled attendant within 7 days, received at least one supplement with a skilled attendant within 7 days. In Tanzania this was asked about all babies (separately for multiple births) but only the first child listed is included here (i.e. one per birth) [↑](#footnote-ref-14)
15. Ibid. [↑](#footnote-ref-15)
16. For these ratings the team did not ask separately about deliveries, so most recent visits here are included even if the most recent visit was for delivery. [↑](#footnote-ref-16)
17. Each baby from a multiple birth is included individually. [↑](#footnote-ref-17)
18. Ibid. [↑](#footnote-ref-18)
19. Ibid. [↑](#footnote-ref-19)
20. Each baby from a multiple birth is included individually. Weight-for-age is measured as proportion of infants (for whom T4D has valid weight measurements) who are below 2 standard deviations from the median WHO Child Growth Standards. [↑](#footnote-ref-20)
21. Each baby from a multiple birth is included individually. Length-for-age is measured as proportion of infants (for whom T4D has valid length measurements) who are below 2 standard deviations from the median WHO Child Growth Standards. [↑](#footnote-ref-21)
22. Includes answers expressed in days, weeks or months that fall within the first trimester and answers "if her menstrual cycle is late" and "as soon as she knows she is pregnant." [↑](#footnote-ref-22)
23. As reported in the T4D Baseline Community Survey [↑](#footnote-ref-23)
24. This is a summary of the protocol followed by the data collection staff of SurveyMeter in Indonesia. For details or clarifications, contact the corresponding authors. [↑](#footnote-ref-24)
25. This is a summary of the protocol followed by the data collection staff of Innovations for Poverty Action in Tanzania. For details or clarifications, contact the corresponding authors. [↑](#footnote-ref-25)
26. Key informants who for the listing were generally not compensated, unless they also assisted the mobilization team as “field guides”, i.e. helping mobilizers navigate the village and find respondents. In each sample village, there were typically 3-4 field guides. Each field guide was given 5000TSH as compensation. [↑](#footnote-ref-26)
27. Interview and observation protocols are available at t4d.ash.harvard.edu. [↑](#footnote-ref-27)
28. We thank Jane Mansbridge for suggesting this approach to systematically understanding individuals’ participation. [↑](#footnote-ref-28)
29. Although the sample in Tanzania included 153 facilities (with 200 catchment-area villages), one of the sample facilities in Dodoma was found to be closed on multiple visits over the course of the survey. Hence, the analysis is based data on facility survey data from 152 facilities, corresponding to 198 villages. [↑](#footnote-ref-29)
